# Triethylamine-Promoted Oxidative Cyclodimerization of 2*H*-Azirine-2-carboxylates to Pyrimidine-4,6-dicarboxylates: Experimental and DFT Study

**DOI:** 10.3390/molecules28114315

**Published:** 2023-05-24

**Authors:** Timofei N. Zakharov, Pavel A. Sakharov, Mikhail S. Novikov, Alexander F. Khlebnikov, Nikolai V. Rostovskii

**Affiliations:** Institute of Chemistry, St. Petersburg State University, 7/9 Universitetskaya Nab., 199034 St. Petersburg, Russia

**Keywords:** azirines, pyrimidines, aziridines, azomethine ylides, cycloaddition, cyclodimerization, triethylamine, hydroxylamines, hippurates, DFT calculations

## Abstract

An unprecedented oxidative cyclodimerization reaction of 2*H*-azirine-2-carboxylates to pyrimidine-4,6-dicarboxylates under heating with triethylamine in air is described. In this reaction, one azirine molecule undergoes formal cleavage across the C-C bond and another across the C=N bond. According to the experimental study and DFT calculations, the key steps of the reaction mechanism include nucleophilic addition of *N*,*N*-diethylhydroxylamine to an azirine to form an (aminooxy)aziridine, generation of an azomethine ylide, and its 1,3-dipolar cycloaddition to the second azirine molecule. The crucial condition for the synthesis of pyrimidines is generation of *N*,*N*-diethylhydroxylamine in the reaction mixture in a very low concentration, which is ensured by the slow oxidation of triethylamine with air oxygen. Addition of a radical initiator accelerated the reaction and resulted in higher yields of the pyrimidines. Under these conditions, the scope of the pyrimidine formation was elucidated, and a series of pyrimidines was synthesized.

## 1. Introduction

**Scheme 1:**
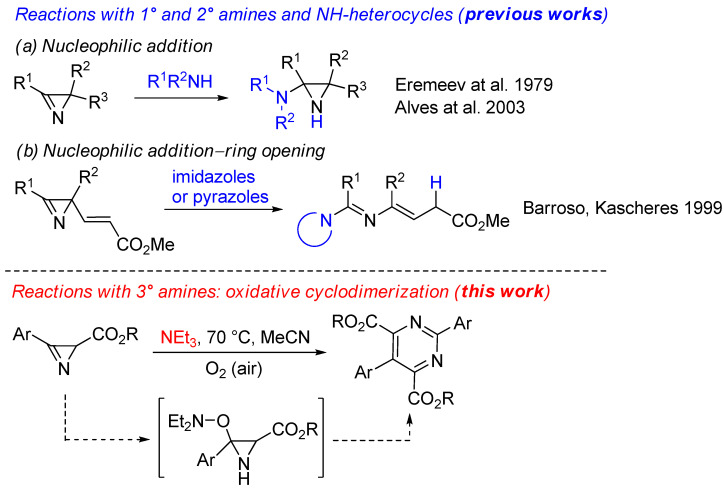
Reactions of 2*H*-azirines with N-nucleophiles [8,9,20].

The 2*H*-azirines are the simplest unsaturated nitrogen heterocycles, possessing great potential in organic synthesis [1,2,3,4,5,6,7,8,9,10]. Mainly, this is due to the presence of an electrophilic C=N bond in a highly strained azirine ring, which can readily undergo addition of various nucleophiles. The reactions of azirines with heteroatomic nucleophiles (alcohols, thiols, phosphites, primary and secondary amines, NH-heterocycles) and C-nucleophiles (some cyclic enols, Grignard reagents) stop at the formation of three-membered heterocycles–aziridines (Scheme 1, reaction 1) [11,12,13,14,15,16,17]. The reactions of azirines with acyclic enols proceed via transient aziridines to form finally, after cyclization, pyrrole derivatives [18,19]. In some cases, addition of O- and N-nucleophiles to azirines also leads to unstable aziridines that readily undergo ring opening across the C-C bond. Such a scenario is realized for 3-(azirinyl)acrylates affording 2-azabutadienes as final products [20] (Scheme 1, reaction 2). Reactions of azirines with tertiary amines have not been reported to date.

In this work, we report an unprecedented dimerization reaction of 2*H*-azirine-2-carboxylates to pyrimidine-4,6-dicarboxylates under heating in air with tertiary amine –triethylamine (Scheme 1, reaction 3). In this reaction, one azirine molecule undergoes formal cleavage across the C-C bond and the other one across the C=N bond. The detailed experimental and theoretical (DFT calculations) study of the reaction mechanism was carried out which allowed identification of the key reaction intermediates—*N*,*N*-diethylhydroxylamine and aminooxyaziridine derivative.

Interestingly, several types of azirine dimerization have been reported previously [21,22,23,24], some of which afforded pyrimidine derivatives. The palladium-catalyzed reaction of 2,3-diphenylazirine gave two types of dimerization products—dihydropyrimidine and diazabicyclohexene [23]. A one-pot procedure for the synthesis of pyrimidine derivatives from α-azidocinnamates has been developed via in situ photochemical formation of 1,3-diazabicyclo[3.1.0]hex-3-enes [24]. It should be noted that this reaction provides pyrimidine-4,5-dicarboxylates that are isomers of the pyrimidines obtained in the current work. In addition, several examples of the formation of a pyrimidine ring from azirines without dimerization are known. In particular, the reaction of methyl 3-(2-methyl-3-phenyl-2*H*-azirin-2-yl)acrylates with formamidine gave pyrimidines [25]. Heating ethyl 3-phenylaziridine-2-carboxylate and 2-bromo-2*H*-azirine in toluene afforded pyrimidine in low yield via 1,3-dipolar cycloaddition of the azomethine ylide, generated from the aziridine, and subsequent transformation of the cycloadduct [26]. The synthesis of pyrimidines by the Cu(I)-catalyzed reaction of 2-methoxy-2*H*-azirines with oxime acetates has recently been reported [27].

## 2. Results and Discussion

### 2.1. Synthesis of Pyrimidines

**Scheme 2:**
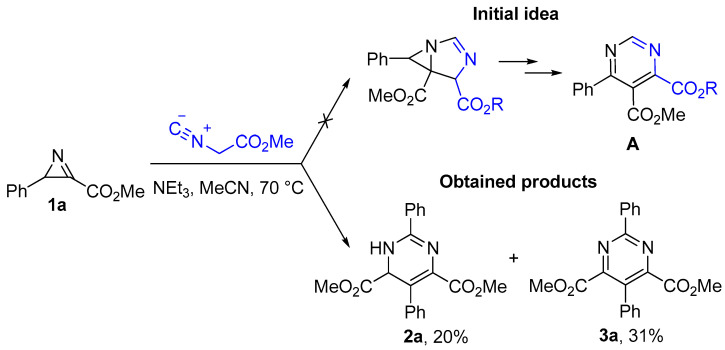
Initial discovery of azirine cyclodimerization.

In search of effective applications of 2*H*-azirines for the synthesis of new pyrimidine derivatives, we carried out a reaction of azirine **1a** with ethyl isocyanoacetate in the presence of bases (Scheme 2). We assumed formation of pyrimidine **A** via a base-catalyzed (3+2)-cycloaddition of the isocyanide to the azirine followed by the ring expansion. To our surprise, after prolonged heating in the presence of triethylamine, dihydropyrimidine **2a** and pyrimidine **3a** were found in the reaction mixture instead of the expected pyrimidine **A**. Compounds **2a** and **3a** result from the dimerization of the starting azirine and do not comprise structural fragments of the ethyl isocyanoacetate. The same results were obtained when an analogous reaction was carried out in the absence of ethyl isocyanoacetate.

We tested various conditions to increase the yield of pyrimidine **3a** and to get data for understanding the reaction mechanism (Table 1). The reactions were carried out under air in closed vials. To simplify the analysis, after complete conversion of the azirine, the reaction mixtures were treated with acetic acid (2 eqv.) and bubbled with air to oxidize dihydropyrimidine **2a** into aromatic pyrimidine **3a**. First, the reaction in the presence of NEt_3_ at 70 °C, but without the isocyanide, was carried out, and pyrimidine **3a** was formed in 48% yield, along with methyl hippurate **4a** (21%) (entry 1). The reaction was found to be temperature-sensitive. The reaction at 100 °C provided pyrimidine **3a** in just 22% yield (entry 2), while at 40 °C it did not occur at all (entry 3). Noteworthily, the formation of pyrimidine **3a** was not observed in the absence of the base, and the azirine was completely recovered (entry 4). The decrease in the NEt_3_ amount led to a slight decrease in yield of pyrimidine **3a** (entry 5). With a stronger nitrogen base such as 1,8-diazabicyclo[5.4.0]undec-7-ene (DBU), the reaction gave lower yield (entry 6), but proceeded much more rapidly than with NEt_3_. When pyridine, DABCO, DIPEA, dimethylaminopyridine (DMAP), morpholine, 4-methylpiperidine, *N*-methylpiperidine, pyrrolidine, imidazole, piperazine, or hexamethyldisilazane (HMDS) were used as a base, the reaction did not proceed at all. Since there was no correlation between the conversion of the azirine and the base strength, the role of Et_3_N did not lie in a simple basic catalysis. The use of *^t^*BuOK caused rapid unselective decomposition of **1a** (entry 7). The reaction with NEt_3_ proceeded much more slowly in low-polar or non-polar solvents such as toluene, acetone, DCE, or 1,4-dioxane (entries 8–11). To check the formation of radical intermediates in the reaction of **1a** with NEt_3_, experiments with such additives as (2,2,6,6-tetramethylpiperidin-1-yl)oxyl (TEMPO), 2,2′-azobis(2-methylpropionitrile) (AIBN), and 1,1′-azobis(cyclohexanecarbonitrile) (ACHN) were conducted (entries 12–14). The former fully suppressed the formation of pyrimidine, while the latter accelerated the reaction and resulted in higher yield of pyrimidine **3a**. It was also found that azirine **1a** did not react with AIBN in the absence of NEt_3_ (entry 15). The use of another radical initiator, benzoyl peroxide, did not give the pyrimidine (entry 16). Finally, no reaction took place under an argon atmosphere, which definitely indicates the participation of oxygen in the reaction (entry 17). Thus, the optimal conditions for the synthesis of pyrimidine **3a** were found to be heating azirine **1a** with 2.6 eqv. of triethylamine at 70 °C in acetonitrile in the presence of 1 eqv. of AIBN or ACHN under air.

**Scheme 3:**
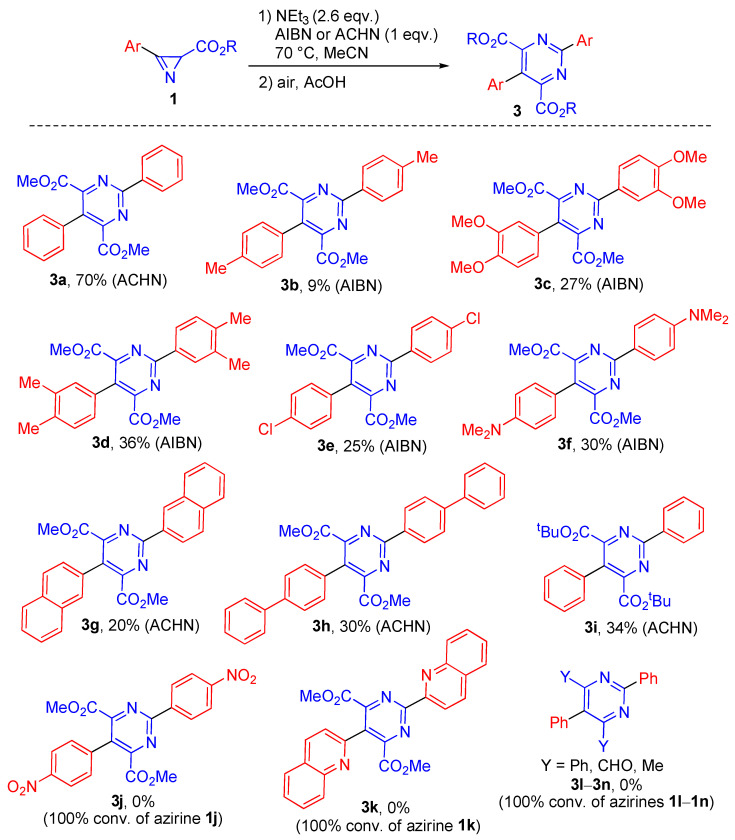
The scope of pyrimidines.

Then, under the optimized conditions, the reaction scope was evaluated (Scheme 3). Pyrimidines bearing electron-donating or weak electron-withdrawing groups in the phenyl ring were obtained, but in lower yields than the parent pyrimidine **3a**. These results are likely due to a complex reaction mechanism (see below). The pyrimidines substituted by the 2-naphthyl or biphenyl group were also prepared by this method in 20% and 30% yield, respectively. The pyrimidine with *tert*-butyl ester groups was prepared in 34% yield. Despite the complete consumption of starting azirines **1j**–**1n**, the pyrimidines possessing strong electron-withdrawing groups in the phenyl ring as well as pyrimidines with Ph, Me, CHO groups at the C4 and C6 of the ring could not be obtained by this method.

### 2.2. Study of the Reaction Mechanism

**Scheme 4:**
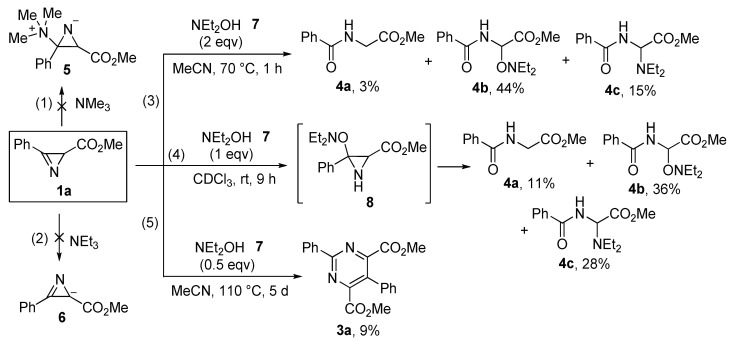
Control experiments.

Originally, we assumed that the reaction starts with a nucleophilic addition of triethylamine to the C=N bond of the azirine (Scheme 4, reaction 1). However, quantum chemical calculations by the DFT method (rwb97xd/6-311+g(d,p)) showed that the adduct of azirine **1a** with trimethylamine (compound **5**) is extremely unstable and must decompose without a barrier into the starting compounds. The formation of pyrimidine **3a** was also observed in the presence of DBU (Table 1, entry 6). In contrast to bulky triethylamine, DBU can undergo nucleophilic addition to multiple bonds [28], and its reaction with azirine **1a** could, in principle, start with the addition to the C=N bond. Another possible first step, the elimination of a proton from the C2 of azirine **1a**, should lead to the formation of the antiaromatic azirinyl anion **6** (Scheme 4, reaction 2). Taking into account the complete inactivity of most tested nitrogen bases in the reaction, the deprotonation stage seems unlikely.

**Figure 1:**
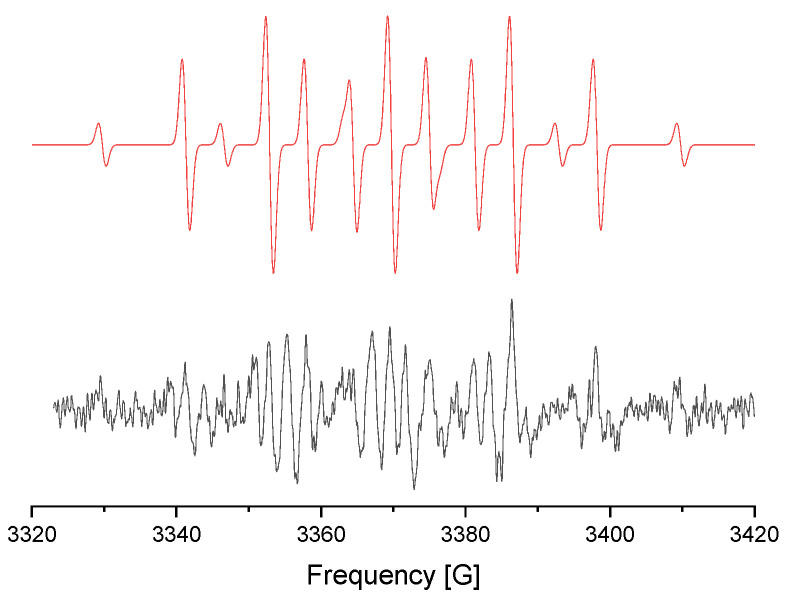
EPR spectrum of nitroxyl radical Et_2_N-O· found in the reaction mixture (bottom—initial spectrum, top—processed spectrum).

Since the addition of a radical initiator resulted in an increase in the reaction rate and product yield (see Table 1, entries 13, 14), we studied the reaction mixture containing azirine **1a**, triethylamine, and acetonitrile by electron paramagnetic resonance spectroscopy (EPR). The experiment showed the presence of triplet of quintets, which corresponds to the nitroxyl radical Et_2_N-O· (Figure 1). This fact indicates that (a) radical processes can take place in the formation of pyrimidines, and (b) not only triethylamine itself but also its oxidation products can be involved in the azirine dimerization process. The formation of the nitroxyl radical Et_2_N-O· from triethylamine in acetonitrile has been described in the literature [29,30].

**Figure 2:**
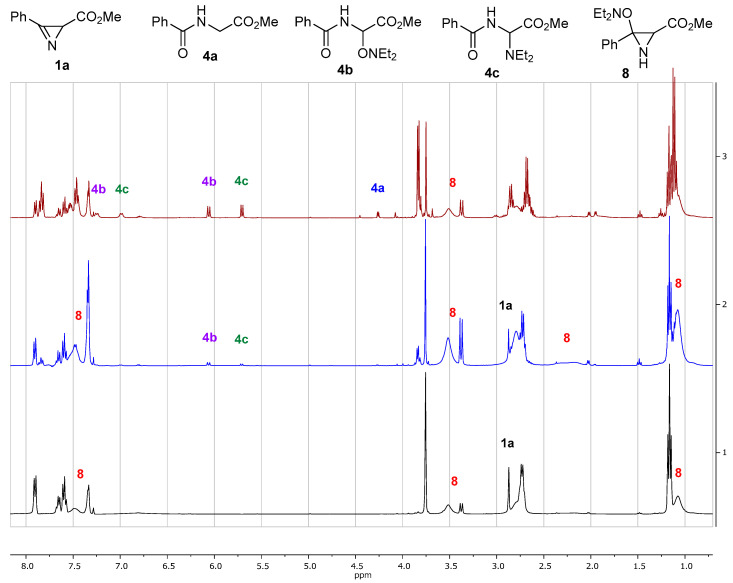
^1^H NMR monitoring of the reaction of azirine **1a** with *N*,*N*-diethylhydroxylamine (**7**) at rt in CDCl_3_ (No. 1, black—1 min; No. 2, blue—9 min; No. 3, brown—60 min).

Encouraged by this finding, we tested *N*,*N*-diethylhydroxylamine (**7**) as a closer potential precursor of the nitroxyl radical Et_2_N-O· (Scheme 4). To our surprise, the reaction of azirine **1a** with 2 eqv. of hydroxylamine **7** at 70 °C was completed in 1 h. Pyrimidine **3a** was not formed, but three other products were isolated: hippuric acid derivatives **4a**–**4c** (reaction 3). The reaction proceeded similarly at room temperature in CDCl_3_ (reaction 4). The detailed ^1^H NMR monitoring of the reaction of azirine **1a** with hydroxylamine **7** showed that an unstable intermediate was formed initially, within 10 min, and then transformed slowly to the final hippurates **4a**–**4c** (Figure 2). The NMR spectra at–40 °C of the crude reaction mixture (see Supporting information for details) was used to assign a structure of (aminooxy)aziridine **8** to the observed intermediate (a mixture of two diastereomers) (Scheme 4, reaction 4). To the best of our knowledge, aziridines with such a substituent have not been reported in the literature.

We hypothesized that unstable aziridine **8** was one of the two components that form the final pyrimidine. Another component, according to our assumption, is the starting azirine. The absence of pyrimidine derivatives **2a** and **3a** among the products of the reactions 3 and 4 in Scheme 4 can be explained by the rapid reaction of hydroxylamine **7** with azirine **1a**, and as a result, removing the latter from the sphere of the reaction. To test this assumption, we reacted azirine **1a** with 0.5 eqv. of hydroxylamine **7** (Scheme 4, reaction 5), which ensured the simultaneous presence of aziridine **8** and azirine **1a** in the reaction mixture. In this case, pyrimidine **3a** indeed was formed, confirming our assumption.

**Scheme 5:**
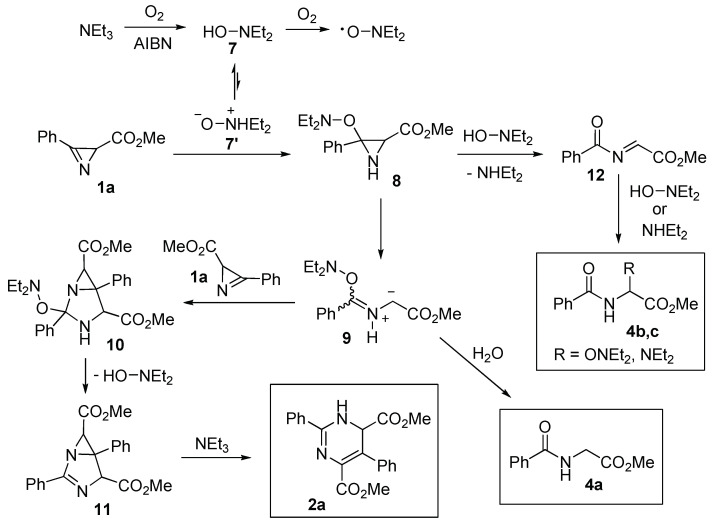
Plausible reaction mechanism.

Based on the above observations, the following mechanism for the formation of dihydropyrimidine **2a** was proposed (Scheme 5). The first stage is a radical oxidation of triethylamine by air oxygen facilitated by addition of a radical initiator and resulted finally in the formation of *N*,*N*-diethylhydroxylamine (**7**). The latter can add to azirine **1a** as is or in the form of amine oxide **7′** to produce aziridine **8**. Then, aziridine **8** can undergo ring opening across the C-C bond to form azomethine ylide **9**. The latter can be evidenced by the presence of hippurate **4a** in the reaction mixture (Table 1, entry 1), which can be interpreted as a hydrolysis product of azomethine ylide **9**. The 1,3-dipolar cycloaddition of azomethine ylide **9** to azirine **1a** affords bicyclic intermediate **10**. The elimination of hydroxylamine **7** from compound **10** followed by the base-catalyzed aziridine ring expansion gives rise to dihydropyrimidine **2a**. The last stages of this mechanistic scheme are partly supported by the known literature data on the synthesis of pyrimidines through the (3+2)-cycloaddition of azomethine ylide to azirine followed by aziridine ring opening [24,26]. The hippurates **4b** and **4c** are likely formed via the HONEt_2_-promoted elimination of NHEt_2_ from aziridine **8** to form imine **12** followed by the nucleophilic addition of *N*,*N*-diethylhydroxylamine or diethylamine to the C=N bond. We assume that the success in synthesis of pyrimidines **2a/3a** is based on suitable reaction conditions that provide a slow generation of *N*,*N*-diethylhydroxylamine and accordingly ensure low concentrations of aziridine **8** and azomethine ylide **9**. The latter can trap the azirine **1a** that is present in large excess.

**Figure 3:**
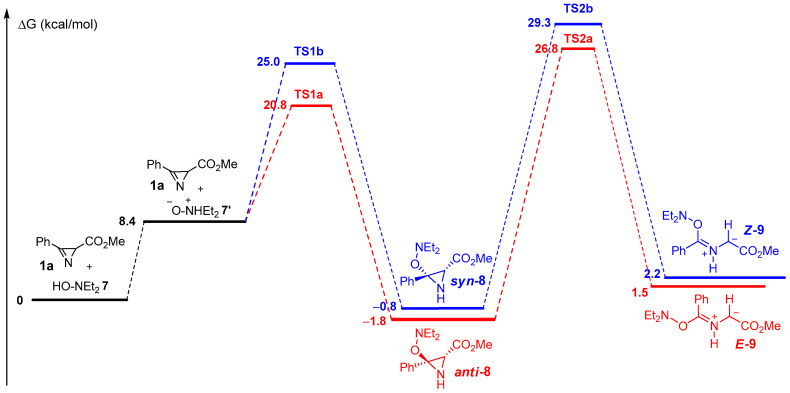
Calculated energy profiles for the transformation of azirine **1a** and *N*,*N*-diethylhydroxylamine **7** to ylides **9**.

**Figure 4:**
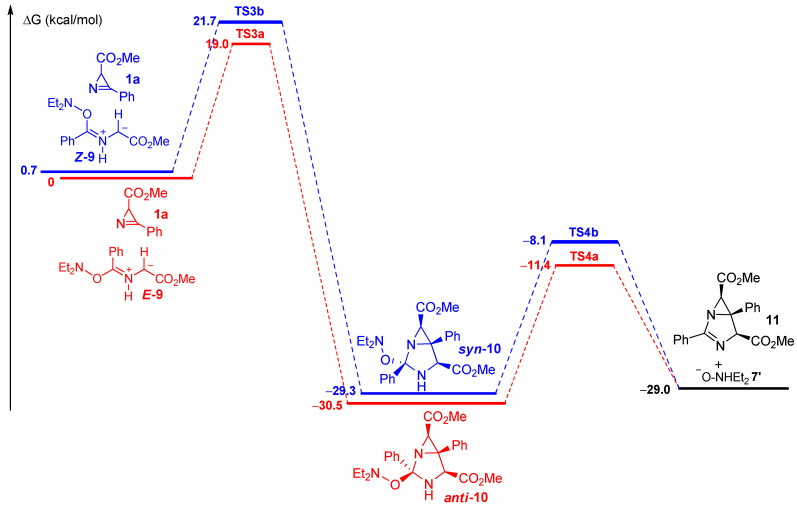
Calculated energy profiles for the transformation of azomethine ylides **9** and azirine **1a** to azirinopyrroline **11**.

The key stages of the pyrimidine formation mechanism were calculated by the density functional theory (DFT) method (rwb97xd/6-311+g(d,p) with PCM solvent model for acetonitrile at 343 K) (Figure 3 and Figure 4). The formation of aziridine **8** theoretically can occur in several alternative ways: the addition of *N*,*N*-diethylhydroxylamine **7**, its tautomer, amine oxide **7′**, or the radical species Et_2_N-O·, observed in the EPR spectrum. It was found that the nucleophilic addition of the hydroxylamine tautomer **7** from a less hindered side of the azirine ring is less favorable (TS1, ∆G^≠^ 50.6 kcal/mol, see Supporting Information for details) than the addition of the isomeric amine oxide **7′**. The attack of the latter at a less hindered side of the azirine to form aziridine ***anti*-8** (TS1a, 12.4 kcal/mol) has a lower barrier than the formation of aziridine ***syn*-8** (TS1b, 16.6 kcal/mol). If calculated from hydroxylamine tautomer **7**, these barriers are 20.8 and 25.0 kcal/mol, respectively. Both aziridines ***syn*-8** and ***anti*-8** are slightly more stable than starting azirine **1a** and hydroxylamine **7** and can be formed reversibly. The attack of the radical Et_2_N-O· to form N-centered aziridine radical takes place with the activation barrier of 44.7 kcal/mol (ub3lyp/6-31g(d) with PCM solvent model for acetonitrile at 343 K) and is hardly possible (see Table S2 in Supporting Information for details). Conrotatory ring opening of aziridines ***anti*-8** (TS2a) and ***syn*-8** (TS2b) across the C–C bond to form azomethine ylides ***E*-9** and ***Z*-9** has barriers of 28.6 and 30.1 kcal/mol, respectively, which can be overcome under heating. Azomethine ylides **9** turned out to be slightly less stable than aziridines **8**. The 1,3-dipolar cycloaddition of azomethine ylides ***E*-9** and ***Z*-9** to azirine **1a** to form azirinopyrrolidines ***anti*-10** (TS3a) and ***syn*-10** (TS3b) has rather low barriers, 19.0 and 21.0 kcal/mol, respectively, and both are thermodynamically favorable reactions. Subsequent elimination of amine oxide **7′** from ***anti*-10** (TS4a) and ***syn*-10** (TS4b) to give azirinopyrroline **11** has barriers of 19.1 and 21.2 kcal/mol correspondingly. Azirinopyrroline **11** should be formed reversibly; further deprotonation with a base shifts the equilibrium to the right. Thus, the proposed mechanism for the formation of dihydropyrimidines is well confirmed by the DFT calculations.

## 3. Materials and Methods

### 3.1. General Instrumentation

Melting points were determined on a melting-point apparatus and were uncorrected. NMR spectra were recorded on Bruker Avance 400 and Bruker Avance 500 spectrometers in CDCl_3_. ^1^H and ^13^C{^1^H} NMR spectra were calibrated according to the residual signal of CDCl_3_ (δ = 7.26 ppm) and the carbon atom signal of CDCl_3_ (δ = 77.0 ppm), respectively. The following abbreviations were used: s—singlet, d—doublet, t—triplet, q—quartet, br.s—broad singlet, m—multiplet. EPR experiment was carried out on a Bruker Elexsys E580 with a modulation amplitude of 1 G and at 323 K. High-resolution mass spectra were recorded with a Bruker maXis HRMS-QTOF, electrospray ionization. Thin-layer chromatography (TLC) was conducted on aluminum sheets precoated with SiO_2_ ALUGRAM SIL G/UV254. Column chromatography was performed on silica gel 60 M (0.04–0.063 mm). Acetonitrile was distilled from phosphorus pentoxide and redistilled from potassium carbonate, and 2,2′-azobis(2-methylpropionitrile) (AIBN) and 1,1′-azobis(cyclohexanecarbonitrile) (ACHN) were purchased and used as received. Azirines **1a**,**b**,**d**,**i**,**j** are known compounds, which were prepared by using the reported procedure [31].

### 3.2. Synthesis and Characterization of 5-Methoxyisoxazoles

General procedure. To a stirred suspension of an isoxazol-5(4*H*)-one (4.6 mmol) in dry diethyl ether (40 mL), a solution of diazomethane in diethyl ether (50 mL), prepared from *N*-methyl-*N*-nitrosourea (9.2 mmol) and potassium hydroxide pellets (42 mmol), was added dropwise at 0 °C. The resulting mixture was stirred at room temperature for 2 h and then concentrated in vacuo. The residue was subjected to column chromatography on silica gel (eluent: hexane–ethylacetate, 3:1) to give a 5-methoxyisoxazole.

*5-Methoxy-3-(naphthalen-2-yl)isoxazole* [32]. Obtained as a pink solid (320 mg, 31%) according to the general procedure. Mp: 129–130 °C (lit. 128–129 °C [32]). ^1^H NMR (400 MHz, CDCl_3_), δ, ppm: 8.21 (s, 1H), 8.00–7.84 (m, 4H), 7.63–7.50 (m, 2H), 5.69 (s, 1H), 4.09 (s, 3H). ^13^C{^1^H} NMR (100 MHz, CDCl_3_), δ, ppm: 174.6, 164.3, 134.1, 133.2, 128.6, 128.5, 127.8, 127.0 (2C), 126.6, 126.3, 123.5, 75.5, 58.9.
*3-(Biphenyl-4-yl)-5-methoxyisoxazole.* Obtained as a colorless solid (740 mg, 64%) according to the general procedure. Mp: 147–148 °C. ^1^H NMR (400 MHz, CDCl_3_), δ, ppm: 7.88–7.83 (m, 2H), 7.73–7.68 (m, 2H), 7.67–7.63 (m, 2H), 7.52–7.46 (m, 2H), 7.43–7.38 (m, 1H), 5.59 (s, 1H), 4.09 (s, 3H). ^13^C{^1^H} NMR (100 MHz, CDCl_3_), δ, ppm: 174.5, 163.9, 142.9, 140.3, 128.9, 128.5, 127.8, 127.5, 127.1, 126.9, 75.4, 58.8. HRMS (ESI-TOF) calculated for C_16_H_13_NO_2_ [M + Na]^+^ 274.0838; found 274.0843.
*5-Methoxy-3-(quinolin-2-yl)isoxazole.* Obtained as a colorless solid (730 mg, 70%) according to the general procedure. Mp: 109–110 °C. ^1^H NMR (400 MHz, CDCl_3_), δ, ppm: 8.25 (d, *J* = 8.6 Hz, 1H), 8.20–8.04 (m, 2H), 7.87 (d, *J* = 8.1, 1H), 7.83–7.71 (m, 1H), 7.66–7.54 (m, 1H), 6.13 (s, 1H), 4.12 (s, 3H). ^13^C{^1^H} NMR (100 MHz, CDCl_3_), δ, ppm: 174.9, 165.3, 148.9, 148.0, 136.8, 129.9, 129.7, 128.4, 127.7, 127.3, 118.5, 76.3, 59.2. HRMS (ESI-TOF) calculated for C_13_H_10_N_2_NaO_2_ [M + Na]^+^ 249.0634; found 249.0638.

### 3.3. Synthesis and Characterization of 2H-Azirines 1

General procedure. FeCl_2_·4H_2_O (10–40 mol%) was added to a solution of a 3-aryl-5-alkoxyisoxazole (1 eqv.) in acetonitrile (2.3–7.8 mL/mmol of 3-aryl-5-alkoxyisoxazole), and the resulting mixture was stirred at room temperature for 2 h (24 h for azirines **1g,h**) until full consumption of the isoxazole (monitored by TLC, eluent: hexane–ethylacetate, 4:1). After completion of the reaction, the solution was filtered through a pad of Celite, concentrated in vacuo, and the product was purified by flash column chromatography on silica gel (eluent: hexane–ethylacetate, 4:1) to give azirines **1a**–**k**.

*Methyl 3-(3,4-dimethoxyphenyl)-2H-azirine-2-carboxylate* (**1c**). Obtained as a colorless solid (97 mg, yield 97%) from 3-(3,4-dimethoxyphenyl)-5-methoxyisoxazole [33] according to the general procedure (40 mol% FeCl_2_·4H_2_O, 1.5 mL of acetonitrile). Mp: 96–97 °C. ^1^H NMR (400 MHz, CDCl_3_), δ, ppm: 7.46–7.42 (m, 2H), 7.02 (d, *J* = 8.8 Hz, 1H), 3.98 (s, 3H), 3.97 (s, 3H), 3.75 (s, 3H), 2.84 (s, 1H). ^13^C{^1^H} NMR (100 MHz, CDCl_3_), δ, ppm: 172.3, 157.5, 153.8, 149.7, 125.4, 114.6, 111.6, 111.1, 56.18, 56.17, 52.2, 29.6. HRMS (ESI-TOF) calculated for C_12_H_13_NNaO_4_ [M + Na]^+^ 258.0737; found 258.0743.
*Methyl 3-(4-chlorophenyl)-2H-azirine-2-carboxylate* (**1e**) [34]. Obtained as a colorless solid (49 mg, yield 98%) from 3-(4-chlorophenyl)-5-methoxyisoxazole [35] according to the general procedure (10 mol% FeCl_2_·4H_2_O, 1.0 mL of acetonitrile). Mp: 66–67 °C (lit. 63.4–64.2 °C [34]). ^1^H NMR (400 MHz, CDCl_3_), δ, ppm: 7.88–7.82 (m, 2H), 7.61–7.56 (m, 2H), 3.77 (s, 3H), 2.89 (s, 1H).
*Methyl 3-(4-(dimethylamino)phenyl)-2H-azirine-2-carboxylate* (**1f**). Obtained as a yellow solid (256 mg, yield 80%) from 3-(4-(dimethylamino)phenyl)-5-methoxyisoxazole [36] according to the general procedure (10 mol% FeCl_2_·4H_2_O, 5 mL of acetonitrile). Mp: 120–121 °C. ^1^H NMR (400 MHz, CDCl_3_), δ, ppm: 7.76–7.71 (m, 2H), 6.81–6.76 (m, 2H), 3.74 (s, 3H), 3.11 (s, 6H), 2.76 (s, 1H). ^13^C{^1^H} NMR (100 MHz, CDCl_3_), δ, ppm: 173.1, 156.2, 153.8, 132.4, 111.6, 108.4, 52.1, 40.1, 28.9. HRMS (ESI-TOF) calculated for C_12_H_14_N_2_NaO_2_ [M + Na]^+^ 241.0947; found 241.0951.
*Methyl 3-(naphthalen-2-yl)-2H-azirine-2-carboxylate* (**1g**) [37]. Obtained as a colorless solid (600 mg, yield 90%) from 5-methoxy-3-(naphthalen-2-yl)isoxazole according to the general procedure (40 mol% FeCl_2_·4H_2_O, 15 mL of acetonitrile). Mp: 67–68 °C (lit. 69–70 °C [37]). ^1^H NMR (400 MHz, CDCl_3_), δ, ppm: 8.33 (s, 1H), 8.04–7.93 (m, 4H), 7.69–7.60 (m, 2H), 3.79 (s, 3H), 2.97 (s, 1H). ^13^C{^1^H} NMR (100 MHz, CDCl_3_), δ, ppm: 172.1, 158.5, 135.8, 132.9, 132.6, 129.4, 129.12, 129.08, 128.1, 127.3, 124.7, 119.5, 52.3, 29.7. HRMS (ESI-TOF) calculated for C_14_H_11_NNaO_2_ [M + Na]^+^ 248.0682; found 248.0685.
*Methyl 3-(biphenyl-4-yl)-2H-azirine-2-carboxylate* (**1h**). Obtained as a colorless solid (195 mg, yield 99%) from 3-(biphenyl-4-yl)-5-methoxyisoxazole according to the general procedure (40 mol% FeCl_2_·4H_2_O, 6 mL of acetonitrile). Mp: 104–105 °C. ^1^H NMR (400 MHz, CDCl_3_), δ, ppm: 8.03–7.93 (m, 2H), 7.87–7.79 (m, 2H), 7.72–7.62 (m, 2H), 7.55–7.49 (m, 2H), 7.49–7.39 (m, 1H), 3.78 (s, 3H), 2.91 (s, 1H). ^13^C{^1^H} NMR (100 MHz, CDCl_3_), δ, ppm: 172.1, 158.2, 146.8, 139.5, 131.0, 129.1, 128.6, 128.0, 127.3, 120.9, 52.3, 29.5. HRMS (ESI-TOF) calculated for C_16_H_13_NNaO_2_ [M + Na]^+^ 274.0838; found 274.0839.
*Methyl 3-(quinolin-2-yl)-2H-azirine-2-carboxylate* (**1k**). Obtained as a brown solid (50 mg, yield 40%) from 5-methoxy-3-(quinolin-2-yl)isoxazole according to the general procedure (10 mol% FeCl_2_·4H_2_O, 3 mL of acetonitrile). Mp: 110–111 °C. ^1^H NMR (400 MHz, CDCl_3_), δ, ppm: 8.40 (d, *J* = 8.4 Hz, 1H), 8.28 (d, *J* = 8.5 Hz, 1H), 8.16 (d, *J* = 8.4 Hz, 1H), 7.94 (d, *J* = 8.1 Hz, 1H), 7.87–7.83 (m, 1H), 7.74–7.70 (m, 1H), 3.80 (s, 3H), 3.17 (s, 1H). ^13^C{^1^H} NMR (100 MHz, CDCl_3_), δ, ppm: 171.6, 160.9, 148.5, 142.7, 137.5, 130.8, 130.6, 129.4, 129.3, 127.8, 121.7, 52.5, 31.5. HRMS (ESI-TOF) calculated for C_13_H_10_N_2_NaO_2_ [M + Na]^+^ 249.0634; found 249.0632.

### 3.4. Reaction of Azirine ***1a*** with Triethylamine

To a solution of azirine **1a** (60 mg, 0.34 mmol) in acetonitrile (1.5 mL), triethylamine (95 mg, 0.94 mmol, 2.6 eqv.) was added. The reaction mixture was heated at 70 °C under stirring for 5 days until full consumption of the azirine (control by TLC, eluent: benzene–ethylacetate, 5:1). The solvent was removed in vacuo, and the residue was subjected to column chromatography on silica gel (eluent: benzene–ethylacetate, 5:1) to give compounds **2a** (12 mg, yield 20%) and **3a** (18 mg, yield 31%).

*Dimethyl 2,5-diphenyl-1,6-dihydropyrimidine-4,6-dicarboxylate* (**2a**). Colorless oil. ^1^H NMR (400 MHz, CDCl_3_), δ, ppm: 7.90–7.86 (m, 2H), 7.76 (br.s, 1H), 7.54–7.46 (m, 3H), 7.40–7.28 (m, 5H), 5.29 (br.s, 1H), 3.71 (s, 3H), 3.63 (s, 3H). ^13^C{^1^H} NMR (100 MHz, CDCl_3_), δ, ppm: 171.0, 163.6, 153.9, 137.2, 133.6, 131.1, 128.8 (2C), 128.0, 127.8, 126.8, 124.3, 118.9, 65.8, 52.41, 52.36. HRMS (ESI-TOF) calculated for C_20_H_19_N_2_O_4_ [M + H]^+^ 351.1339; found 351.1350.
*Dimethyl 2,5-diphenylpyrimidine-4,6-dicarboxylate* (**3a**) [38]. Mp: 160–161 °C (lit. 161–163 °C [38]). ^1^H NMR (400 MHz, CDCl_3_), δ, ppm: 8.58–8.52 (m, 2H), 7.55–7.50 (m, 3H), 7.49–7.42 (m, 3H), 7.37–7.32 (m, 2H), 3.76 (s, 6H). ^13^C{^1^H} NMR (100 MHz, CDCl_3_), δ, ppm: 165.4, 163.5, 158.6, 135.8, 132.8, 131.6, 128.9, 128.7, 128.61, 128.58, 128.4, 127.8, 52.8.

### 3.5. Synthesis and Characterization of Pyrimidines ***3***

General procedure. A solution of an azirine (0.34 mmol, 1 eqv.), triethylamine (95 mg, 0.94 mmol, 2.6 eqv.), and AIBN or ACHN (0.34 mmol, 1 eqv.) in acetonitrile (1.5 mL) was stirred in a screw-cap tube at 70 °C for 3–7 days (monitored by TLC, eluent: ethylacetate–benzene, 1:1). Then the reaction mixture was bubbled with air until complete conversion of dihydropyrimidine to pyrimidine (about 20 min, monitored by TLC, eluent: ethylacetate–benzene, 1:1). After that, the reaction mixture was concentrated in vacuo, and the residue was subjected to column chromatography on silica gel to give pyrimidine.

*Dimethyl 2,5-diphenylpyrimidine-4,6-dicarboxylate* (**3a**) [38]. Obtained as a colorless solid (42 mg, yield 70%) according to the general procedure (initiator: ACHN, 3 days, eluent: benzene–ethylacetate, 5:1).
*Dimethyl 2,5-di(p-tolyl)pyrimidine-4,6-dicarboxylate* (**3b**) [38]. Obtained as a colorless solid (6 mg, yield 9%) according to the general procedure (initiator: AIBN, 7 days, eluent: hexane–ethylacetate, 2:1). Mp: 145–146 °C (lit. 144–146 °C [38]). ^1^H NMR (400 MHz, CDCl_3_), δ, ppm: 8.47–8.40 (m, 2H), 7.36–7.30 (m, 2H), 7.27–7.19 (m, 4H), 3.78 (s, 6H), 2.46 (s, 3H), 2.43 (s, 3H). ^13^C{^1^H} NMR (100 MHz, CDCl_3_), δ, ppm: 165.7, 163.4, 158.6, 142.0, 138.8, 133.2, 129.8, 129.4, 129.2, 128.7, 128.5, 127.5, 52.8, 21.6, 21.4. HRMS (ESI-TOF) calculated for C_22_H_20_N_2_NaO_4_ [M + Na]^+^ 399.1315; found 399.1318.
*Dimethyl 2,5-di(3,4-dimethoxyphenyl)pyrimidine-4,6-dicarboxylate* (**3c**) [38]. Obtained as a pale yellow solid (20 mg, yield 27%) according to the general procedure (initiator: AIBN, 7 days, eluent: hexane–ethylacetate, 3:1). Mp: 163–165 °C (lit. 164–166 °C [38]). ^1^H NMR (400 MHz, CDCl_3_), δ, ppm: 8.18 (dd, *J* = 8.5, 2.0 Hz, 1H), 8.05 (d, *J* = 2.0 Hz, 1H), 6.98 (d, *J* = 8.5 Hz, 1H), 6.95–6.86 (m, 3H), 4.03 (s, 3H), 3.99 (s, 3H), 3.95 (s, 3H), 3.90 (s, 3H), 3.79 (s, 6H). ^13^C{^1^H} NMR (100 MHz, CDCl_3_), δ, ppm: 165.8, 162.9, 158.7, 152.2, 149.5, 149.1, 148.9, 128.7, 126.3, 125.0, 122.6, 121.5, 111.8, 111.1, 111.0, 110.8, 56.1, 56.00, 55.96, 55.8, 52.9.
*Dimethyl 2,5-di(3,4-dimethylphenyl)pyrimidine-4,6-dicarboxylate* (**3d**). Obtained as a colorless solid (25 mg, yield 36%) according to the general procedure (initiator: AIBN, 7 days, eluent: benzene–ethylacetate, 5:1). Mp: 173–174 °C. ^1^H NMR (400 MHz, CDCl_3_), δ, ppm: 8.30 (s, 1H), 8.29–8.24 (m, 1H), 7.31–7.25 (m, 1H), 7.19 (d, *J* = 7.7 Hz, 1H), 7.10 (s, 1H), 7.09–7.04 (m, 1H), 3.79 (s, 6H), 2.39 (s, 3H), 2.36 (s, 3H), 2.33 (s, 3H), 2.31 (s, 3H). ^13^C{^1^H} NMR (125 MHz, CDCl_3_), δ, ppm: 165.8, 163.4, 158.6, 140.7, 137.4, 136.9, 136.8, 133.5, 130.2, 130.0, 129.8, 129.7, 129.6, 127.3, 126.3, 126.0, 52.8, 19.9, 19.80, 19.76, 19.7. HRMS (ESI-TOF) calculated for C_24_H_24_N_2_NaO_4_ [M + Na]^+^ 427.1628; found 427.1631.
*Dimethyl 2,5-di(4-chlorophenyl)pyrimidine-4,6-dicarboxylate* (**3e**) [38]. Obtained as a bright-yellow solid (18 mg, yield 25%) according to the general procedure (initiator: AIBN, 4 days, eluent: hexane–ethylacetate, 3:1). Mp: 137–138 °C (lit. 139–140 °C [38]). ^1^H NMR (400 MHz, CDCl_3_), δ, ppm: 8.52–8.49 (m, 2H), 7.51–7.49 (m, 2H), 7.46–7.44 (m, 2H), 7.28–7.26 (m, 2H), 3.80 (s, 6H).
*Dimethyl 2,5-di(4-dimethylaminophenyl)pyrimidine-4,6-dicarboxylate* (**3f**). Obtained as a bright-yellow solid (22 mg, yield 30%) according to the general procedure (initiator: AIBN, 7 days, eluent: hexane–ethylacetate, 2:1). Mp: 216–217 °C. ^1^H NMR (400 MHz, CDCl_3_), δ, ppm: 8.41–8.38 (m, 2H), 7.19–7.17 (m, 2H), 6.77–6.73 (m, 4H), 3.79 (s, 6H), 3.08 (s, 6H), 3.02 (s, 6H). ^13^C{^1^H} NMR (125 MHz, CDCl_3_), δ, ppm: 166.4, 162.9, 158.5, 152.5, 150.3, 130.1, 129.5, 125.8, 123.7, 120.2, 112.0, 111.4, 52.7, 40.24, 40.29. HRMS (ESI-TOF) calculated for C_24_H_26_N_4_NaO_4_ [M + Na]^+^ 457.1846; found 457.1852.
*Dimethyl 2,5-di(naphthalen-2-yl)pyrimidine-4,6-dicarboxylate* (**3g**) [38]. Obtained as a pale-yellow solid (15 mg, yield 20%) according to the general procedure (initiator: ACHN, 6 days, eluent: benzene–ethylacetate, 5:1). Mp: 196–197 °C (lit. 197–198 °C [38]). ^1^H NMR (400 MHz, CDCl_3_), δ, ppm: 9.15 (s, 1H), 8.66 (dd, *J* = 8.6, 1.8 Hz, 1H), 8.10–8.03 (m, 1H), 8.00 (d, *J* = 8.6 Hz, 1H), 7.97–7.88 (m, 4H), 7.84 (s, 1H), 7.63–7.53 (m, 4H), 7.48 (dd, *J* = 8.6, 1.8 Hz, 1H), 3.74 (s, 6H). ^13^C{^1^H} NMR (125 MHz, CDCl_3_), δ, ppm: 165.6, 163.6, 158.9, 135.2, 133.2, 133.14, 133.09, 133.0, 130.4, 129.7, 129.5, 128.5, 128.3 (2C), 128.0, 127.89, 127.87, 127.8, 127.7, 127.0, 126.8, 126.5, 126.3, 125.2, 53.0. HRMS (ESI-TOF) calculated for C_28_H_20_N_2_NaO_4_ [M + Na]^+^ 471.1315; found 471.1307.
*Dimethyl 2,5-di(4-biphenyl)pyrimidine-4,6-dicarboxylate* (**3h**). Obtained as a colorless solid (30 mg, yield 36%) according to the general procedure (initiator: ACHN, 6 days, eluent: benzene–ethylacetate, 1:1). Mp: 222–223 °C. ^1^H NMR (400 MHz, CDCl_3_), δ, ppm: 8.67–8.61 (m, 2H), 7.80–7.75 (m, 2H), 7.74–7.66 (m, 6H), 7.54–7.48 (m, 4H), 7.46–7.39 (m, 4H), 3.81 (s, 6H). ^13^C{^1^H} NMR (125 MHz, CDCl_3_), δ, ppm: 165.6, 163.3, 158.7, 144.3, 141.6, 140.3, 140.0, 134.7, 131.7, 129.3, 129.1, 128.92, 128.88, 127.9, 127.8, 127.5, 127.4, 127.2, 127.13, 127.09, 53.0. HRMS (ESI-TOF) calculated for C_32_H_24_N_2_NaO_4_ [M + Na]^+^ 523.1628; found 523.1616.
*Di-tert-butyl 2,5-diphenylpyrimidine-4,6-dicarboxylate* (**3i**) [38]. Obtained as a colorless solid (25 mg, yield 34%) according to the general procedure (initiator: ACHN, 3 days, eluent: benzene–ethylacetate, 5:1). Mp: 128–129 °C (lit. 124–125 °C [38]). ^1^H NMR (400 MHz, CDCl_3_), δ, ppm: 8.61–8.57 (m, 2H), 7.56–7.49 (m, 3H), 7.49–7.43 (m, 3H), 7.41–7.34 (m, 2H), 1.28 (s, 18H). ^13^C{^1^H} NMR (100 MHz, CDCl_3_), δ, ppm: 164.3, 163.6, 159.5, 136.2, 133.7, 131.3, 129.5, 128.8, 128.53, 128.49, 128.2, 126.6, 83.8, 27.6.

### 3.6. Reaction of Azirine ***1a*** with N,N-Diethylhydroxylamine

To a solution of azirine **1a** (60 mg, 0.34 mmol) in acetonitrile (1.5 mL), *N*,*N*-diethylhydroxylamine (70 mg, 0.75 mmol) was added. The reaction mixture was heated at 70 °C under stirring for 1 h until full consumption of the azirine (control by TLC, eluent: benzene–ethylacetate, 5:1). The solvent was removed in vacuo, and the residue was subjected to column chromatography on silica gel (eluent: benzene–ethylacetate, 5:1) to give compounds **4a** (2 mg, yield 3%), **4b** (42 mg, yield 44%) and **4c** (13 mg, yield 15%).

*Methyl 2-benzamidoacetate* (**4a**) [39]. ^1^H NMR (400 MHz, CDCl_3_), δ, ppm: 7.86–7.82 (m, 2H), 7.58–7.52 (m, 1H), 7.50–7.44 (m, 2H), 6.69 (br.s, 1H), 4.28 (d, *J* = 5.1 Hz, 2H), 3.83 (s, 3H).
*Methyl 2-benzamido-2-((diethylamino)oxy)acetate* (**4b**). Yellow oil. ^1^H NMR (400 MHz, CDCl_3_), δ, ppm: 7.93–7.82 (m, 2H), 7.59–7.53 (m, 1H), 7.51–7.44 (m, 2H), 7.23 (br.d, *J* = 9.3 Hz, 1H), 6.07 (d, *J* = 9.3 Hz, 1H), 3.85 (s, 3H), 2.86 (m, 4H), 1.12 (t, *J* = 7.1 Hz, 6H). ^13^C{^1^H} NMR (100 MHz, CDCl_3_), δ, ppm: 168.8 (C(O)O), 166.6 (C(O)N), 133.5 (*ipso*-C), 132.2 (*para*-C), 128.7 (*meta*-C), 127.2 (*ortho*-C), 79.6 (CH), 52.6 (CH_3_O), 52.2 (CH_2_N), 11.7 (CH_3_). HRMS (ESI-TOF) calculated for C_14_H_20_N_2_NaO_4_ [M + Na]^+^ 303.1315; found 303.1311.
*Methyl 2-benzamido-2-(diethylamino)acetate* (**4c**). Yellow oil. ^1^H NMR (400 MHz, CDCl_3_), δ, ppm: 7.85–7.80 (m, 2H), 7.58–7.51 (m, 1H), 7.50–7.42 (m, 2H), 6.99 (br.d, *J* = 8.3 Hz, 1H), 5.71 (d, *J* = 8.3 Hz, 1H), 3.83 (s, 3H), 2.76–2.60 (m, 4H), 1.18 (t, *J* = 7.1 Hz, 6H). ^13^C{^1^H} NMR (125 MHz, CDCl_3_), δ, ppm: 171.2 (C(O)O), 167.7 (C(O)N), 133.9 (*ipso*-C), 131.8 (*para*-C), 128.6 (*meta*-C), 127.1 (*ortho*-C), 66.5 (CH), 52.8 (CH_3_O), 44.1 (CH_2_N), 13.4 (CH_3_). HRMS (ESI-TOF) calculated for C_14_H_20_N_2_NaO_3_ [M + Na]^+^ 287.1366; found 287.1371.

### 3.7. NMR Detection of Aziridine ***8***

To a solution of azirine **1a** (20 mg, 0.11 mmol) in CDCl_3_ (0.5 mL) in standard NMR tube, *N*,*N*-diethylhydroxylamine (11 mg, 0.11 mmol) was added. The reaction mixture was kept at room temperature for 10 min and then immediately cooled with hexane–liquid nitrogen mixture. NMR spectra of this reaction mixture at –40 °C showed the presence of two diastereomers of aziridine **8** in a 1.2:1 ratio along with unreacted starting compounds.

*Methyl 3-(diethylaminooxy)-3-phenylaziridine-2-carboxylate (**8**).* ^1^H NMR (500 MHz, CDCl_3_,_–_40 °C), δ, ppm: 7.58–7.53 (m, 2.5H, *ortho*-H, dia-2), 7.45–7.33 (m, 8.5H), 3.61 (s, 3H, CH_3_O, dia-1), 3.45 (s, 3.5H, CH_3_O, dia-2), 3.43 (d, *J* = 8.9 Hz, 1H, CH, dia-1), 3.36 (d, *J* = 10.2 Hz, 1.2H, CH, dia-2), 2.85–2.56 (m), 2.44 (d, *J* = 10.2 Hz, 1.2H, NH, dia-2), 2.19 (d, *J* = 8.9 Hz, 1H, NH, dia-1), 1.24–1.07 (m), 0.99 (t, *J* = 7.1 Hz, 3H, CH_3_, dia-1). ^13^C{^1^H} NMR (125 MHz, CDCl_3_,–40 °C), δ, ppm: aziridine carbons 78.0 (C, dia-1), 77.8 (C, dia-2), 38.7 (CH, dia-1), 37.2 (CH, dia-2).

### 3.8. EPR Detection of Nitroxyl Radical Et_2_N-O·

The reaction mixture of azirine **1a** (60 mg, 0.34 mmol) and triethylamine (90 mg, 0.88 mmol) in acetonitrile (1.5 mL) was stirred at 70 °C for 12 h. After cooling, a few drops of water were added to this reaction mixture to increase the signal resolution. The mixture was transferred into an EPR vial. EPR spectrum of this reaction mixture at 50 °C showed the presence of nitroxyl radical Et_2_N-O·.

## 4. Conclusions

An unprecedented dimerization reaction of 2*H*-azirine-2-carboxylates to pyrimidine-4,6-dicarboxylates under heating with triethylamine in air has been described. In this reaction, one azirine molecule undergoes cleavage across the C-C bond and another across the C=N bond. According to the experimental study and DFT calculations, the key stages of the reaction mechanism includes oxidation of triethylamine by atmospheric oxygen into *N*,*N*-diethylhydroxylamine, formation of (aminooxy)aziridine, generation of azomethine ylide and its further 1,3-dipolar cycloaddition to the second azirine molecule. Success in the synthesis of pyrimidines is based on suitable reaction conditions that provide slow generation of *N*,*N*-diethylhydroxylamine. Addition of a radical initiator accelerated the reaction and resulted in higher yield of the pyrimidines. Under these conditions, the scope of the pyrimidine formation was elucidated, and a series of pyrimidines was synthesized.

## Figures and Tables

**Table 1:** Optimization of the pyrimidine synthesis ^a^.

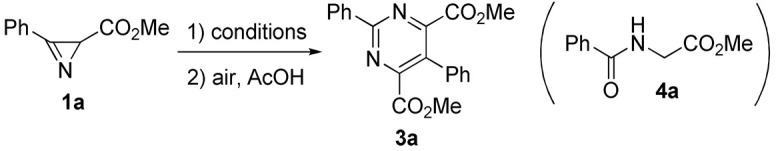						
Entry	Solvent	Base	Additive	T, °C	Time ^b^	Yield of 3a, %/ Conversion of 1a, %
1	MeCN	NEt_3_ (2 eqv)	-	70	4.5 days	48/100
2	MeCN	NEt_3_ (2 eqv)	-	100	34 h	22/100
3	MeCN	NEt_3_ (2 eqv)	-	40	5 days	0/0
4	MeCN	-	-	70	4.5 days	0/0
5	MeCN	NEt_3_ (1 eqv)	-	70	3 weeks	41/100
6	MeCN	DBU (1 eqv)	-	70	18 h	27/100
7	MeCN	*^t^*BuOK (1 eqv)	-	rt	1 h	0/100
8	PhMe	NEt_3_ (2 eqv)	-	70	6 days	0/85 (^1^H NMR)
9	Acetone	NEt_3_ (2 eqv)	-	70	1 week	0/19 (^1^H NMR)
10	DCE	NEt_3_ (2 eqv)	-	70	8 days	0/66 (^1^H NMR)
11	1,4-Dioxane	NEt_3_ (2 eqv)	-	70	5 days	0/0
12	MeCN	NEt_3_ (2.6 eqv)	TEMPO (1 eqv)	70	5 days	0/0
13	MeCN	NEt_3_ (2.6 eqv)	AIBN (1 eqv)	70	5 days	70/100
14	MeCN	NEt_3_ (2.6 eqv)	ACHN (1 eqv)	70	3 days	70/100
15	MeCN	-	AIBN (1 eqv)	70	24 h	0/0
16	MeCN	NEt_3_ (2.6 eqv)	(BzO)_2_ (1 eqv)	70	3 days	0/100
17	MeCN	NEt_3_ (2 eqv)	under Ar	70	5 days	0/0

^a^ Isolated yields unless otherwise noted. ^b^ Time until full consumption of **1a**.

## Data Availability

Data are contained within the article.

## Supplementary Materials

The following supporting information can be downloaded at https://www.mdpi.com/article/10.3390/molecules28114315/s1. NMR spectra of compounds **1**, **2a**, **3**, **4**, **8**; EPR spectroscopy details; quantum chemical calculation details. Table S1: Energies (au) and cartesian coordinates of stationary points for compounds **1a**, **7**, **7′**, ***anti*-8**, ***syn*-8**, ***E*-9**, ***Z*-9**, ***anti*-10**, ***syn*-10**, **11** and transition states TS1, TS1a, TS1b, TS2a, TS2b, TS3a, TS3b, TS4a, TS4b (calculations in rwb97xd/6-311+g(d,p)); Table S2: Energies (au) and cartesian coordinates of stationary points for compounds **1a** and Et_2_N-O· and transition state TS1^rad^ (calculations in ub3lyp/6-31g(d)). Reference [40] is cited in the supplementary materials.

## Sample Availability

Samples of compounds **1**, **3**, **4** are available from the authors.
