# Supplementary material for: Triethylamine-Promoted Oxidative Cyclodimerization of 2H-Azirine-2-carboxylates to Pyrimidine-4,6-dicarboxylates: Experimental and DFT Study
Source: Molecules. 2023 May 24;28(11):4315. doi: 10.3390/molecules28114315 (PMC10254727; doi:10.3390/molecules28114315)
Supplement: Supplementary file 1 [file molecules-28-04315-s001.zip › molecules-2407675-supplementary.pdf]

Supporting information for

Triethylamine-Promoted Oxidative Cyclodimerization of 2H-Azirine-2-carboxylates to Pyrimidine-4,6-dicarboxylates: Experimental and DFT Study

Timofei N. Zakharov, Pavel A. Sakharov, Mikhail S. Novikov, Alexander F. Khlebnikov and Nikolai V. Rostovskii *

Institute of Chemistry, St. Petersburg State University, 7/9 Universitetskaya Nab., 199034 St. Petersburg, Russia
* Correspondence: n.rostovskiy@spbu.ru

Table of contents

I. NMR spectroscopy....................S2
II. EPR spectroscopy S30
III. Calculations details S31

I. NMR spectroscopy

^{1}H NMR (400 MHz, $CDCl_3$) spectrum of 5-methoxy-3-(naphthalen-2-yl)isoxazole

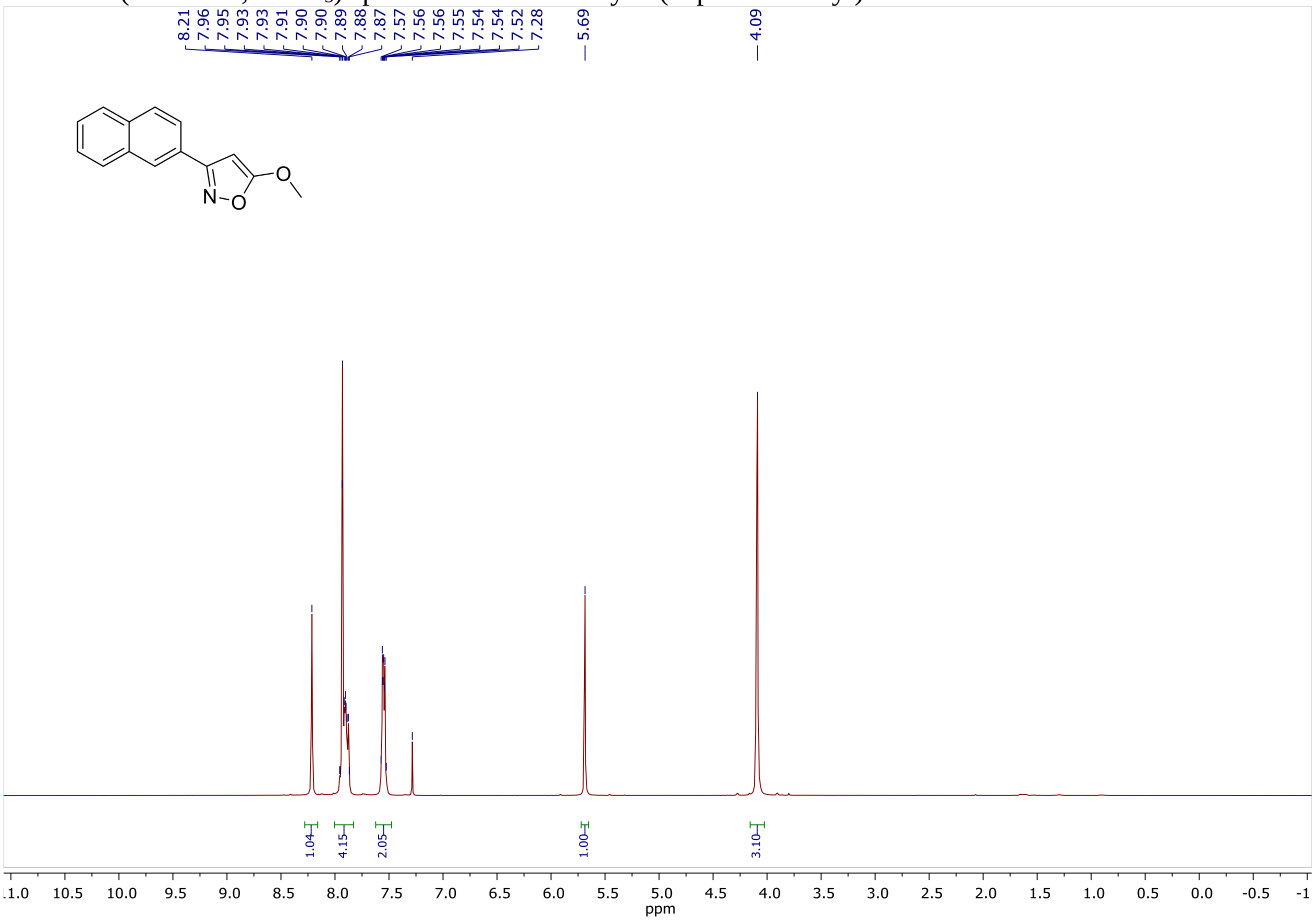

^{13}C{^{1}H} NMR (100 MHz, $CDCl_3$) spectrum of 5-methoxy-3-(naphthalen-2-yl)isoxazole

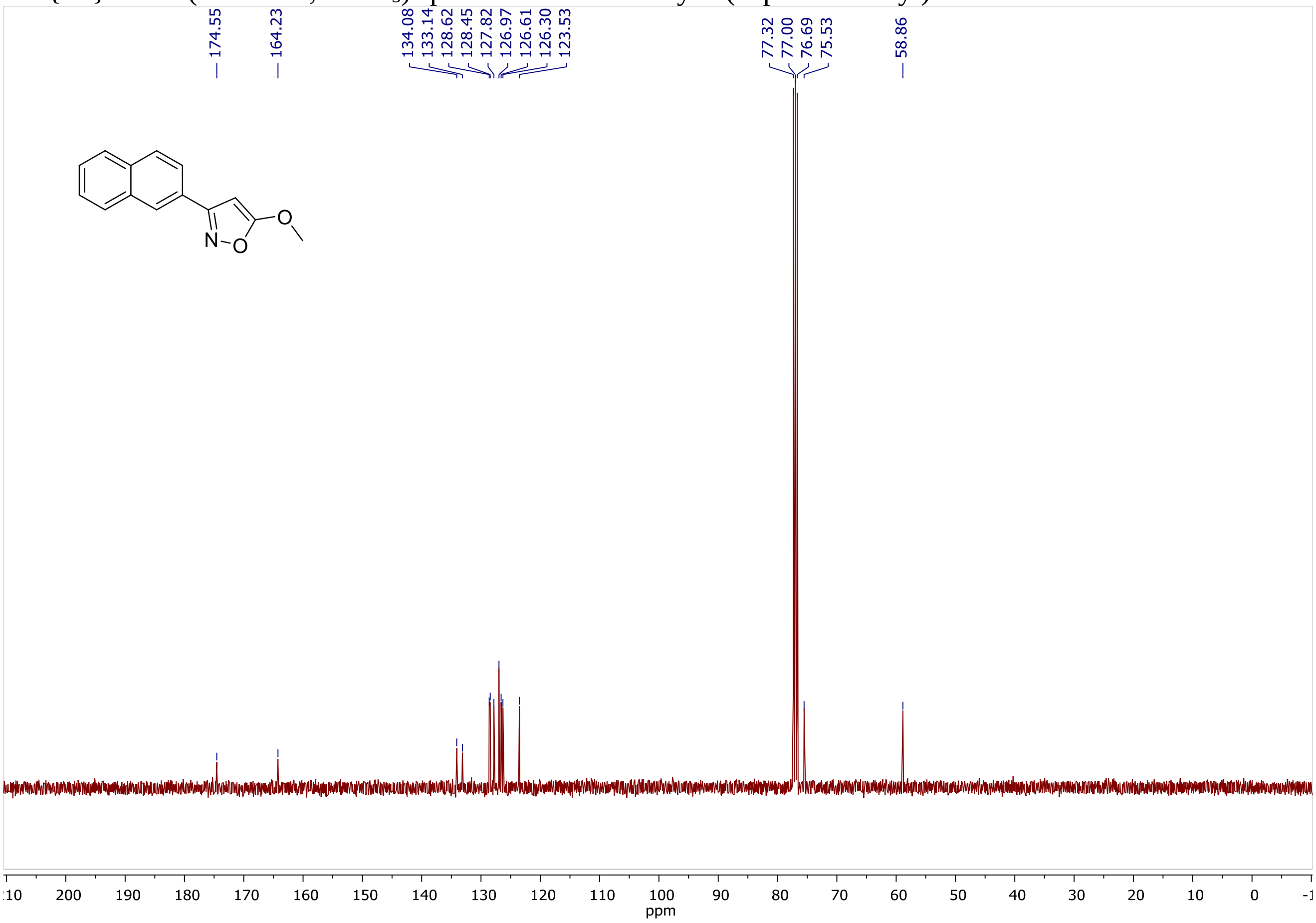

^{1}H NMR (400 MHz, $CDCl_3$) spectrum of 3-(biphenyl-4-yl)-5-methoxyisoxazole

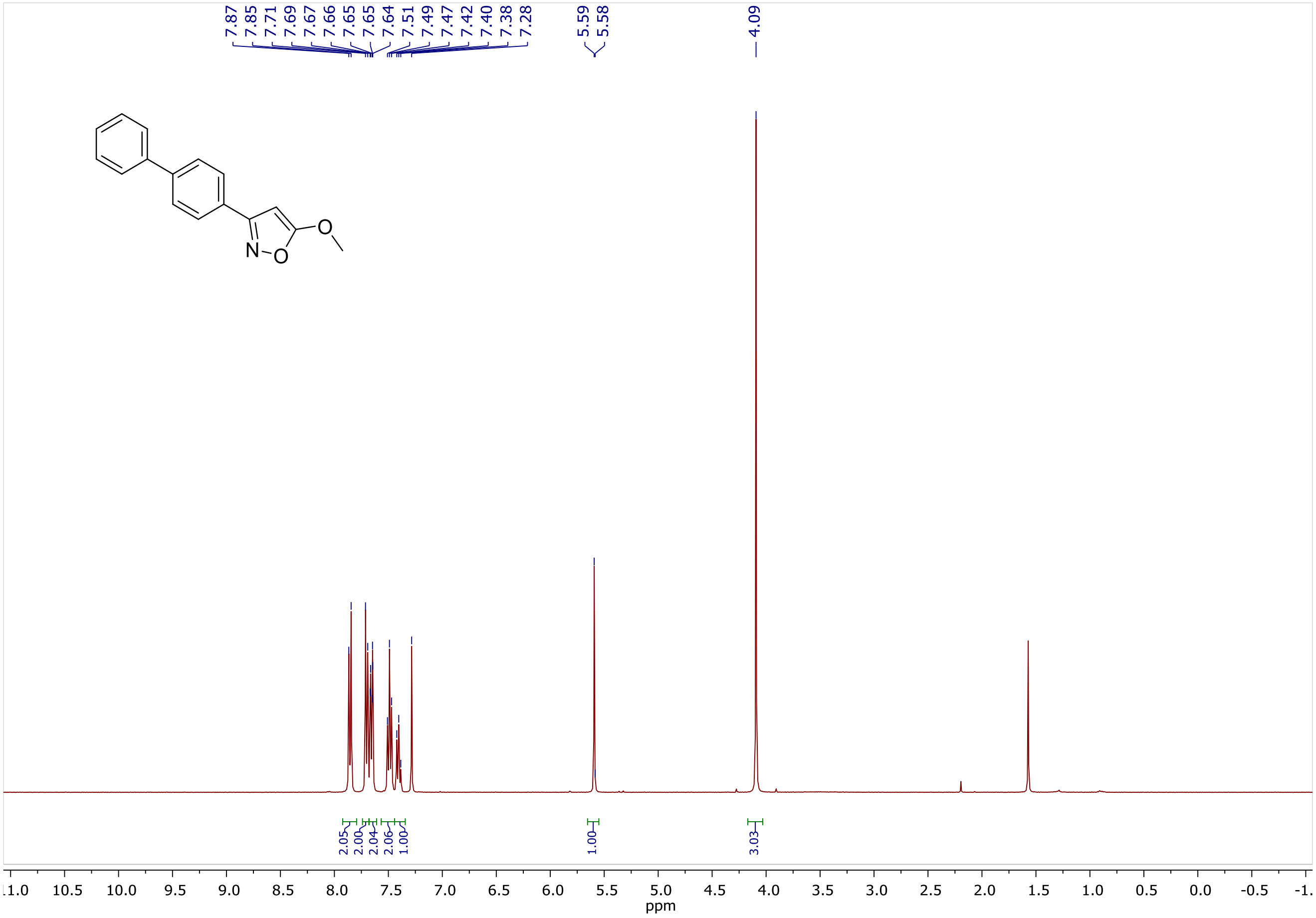

$^{13}C\{^{1}H\}$ NMR (100 MHz, $CDCl_3$) spectrum of 3-(biphenyl-4-yl)-5-methoxyisoxazole

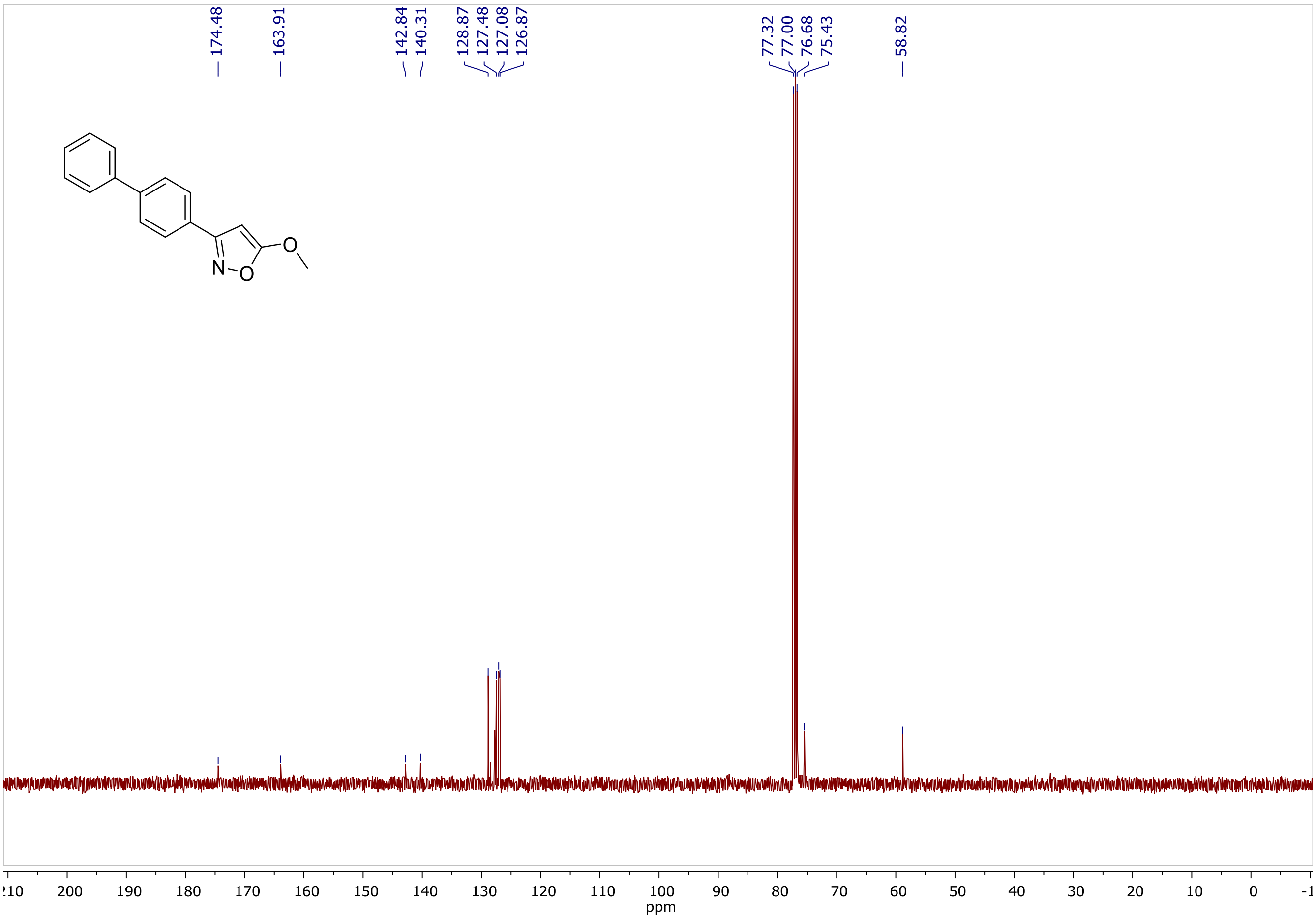

^{1}H NMR (400 MHz, $CDCl_3$) spectrum of 5-methoxy-3-(quinolin-2-yl)isoxazole

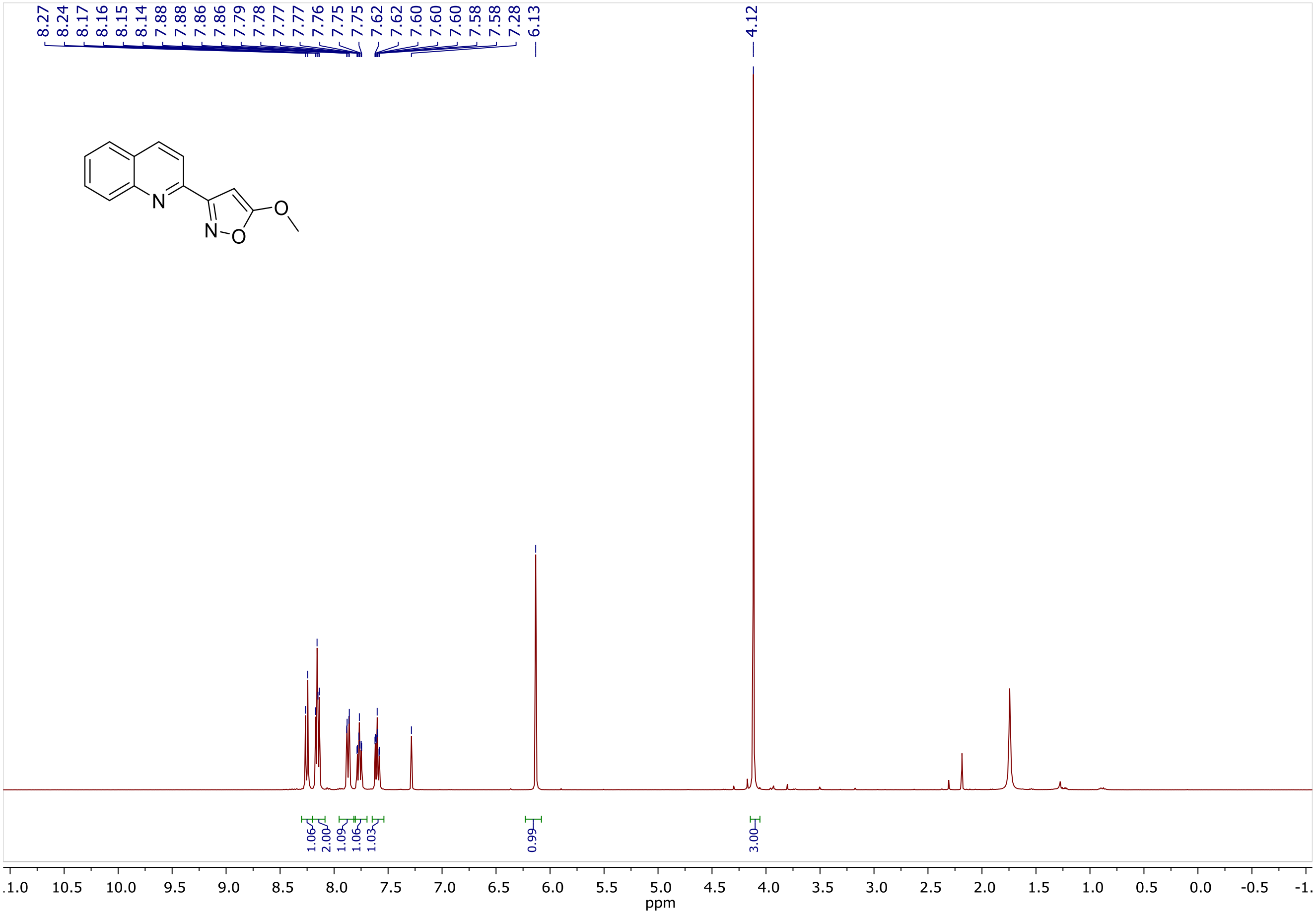

$^{13}C\{^{1}H\}$ NMR (100 MHz, $CDCl_3$) spectrum of 5-methoxy-3-(quinolin-2-yl)isoxazole

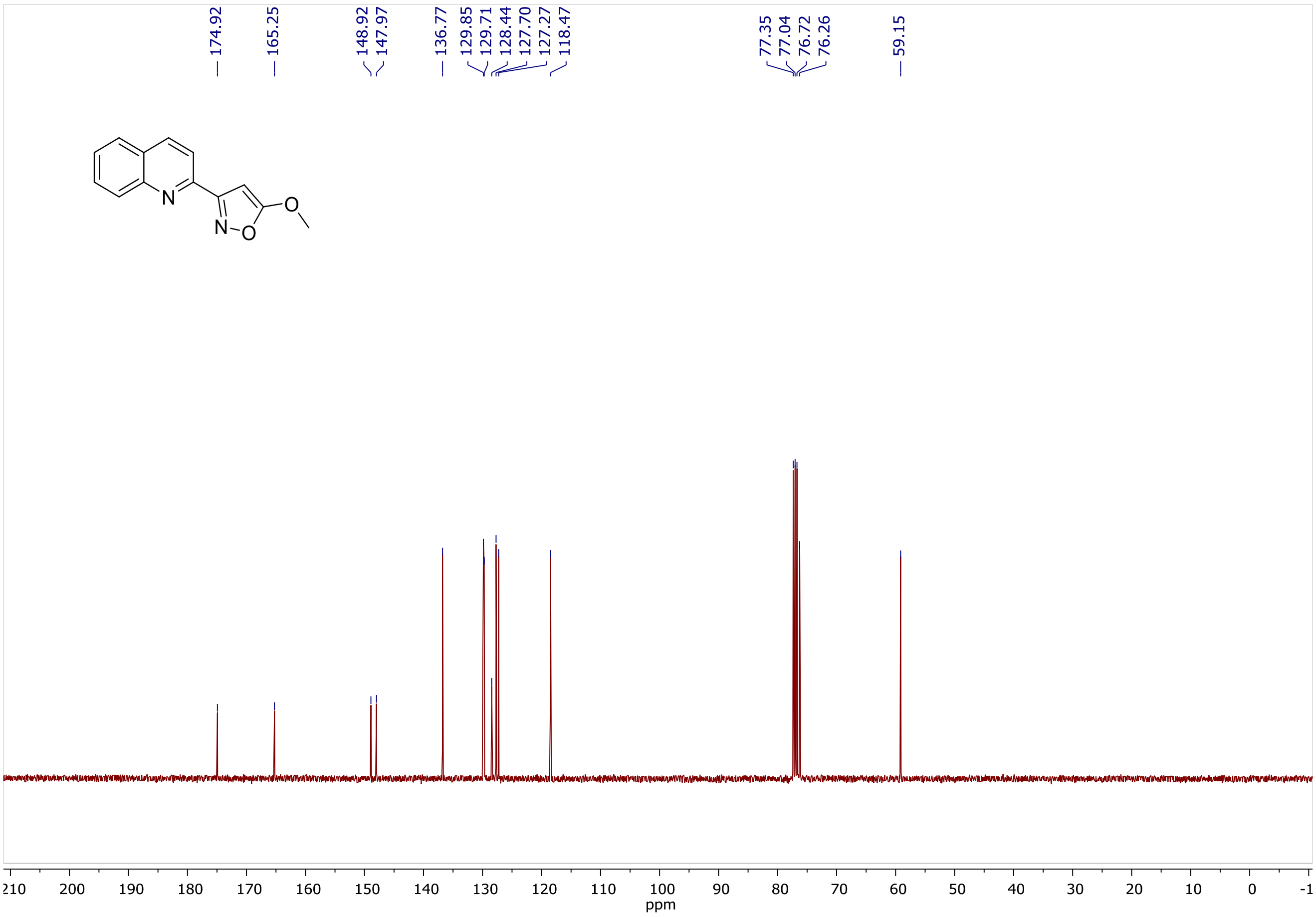

^{1}H NMR (400 MHz, $CDCl_3$) spectrum of methyl 3-(3,4-dimethoxyphenyl)-*2H*-azirine-2-carboxylate (**1c**)

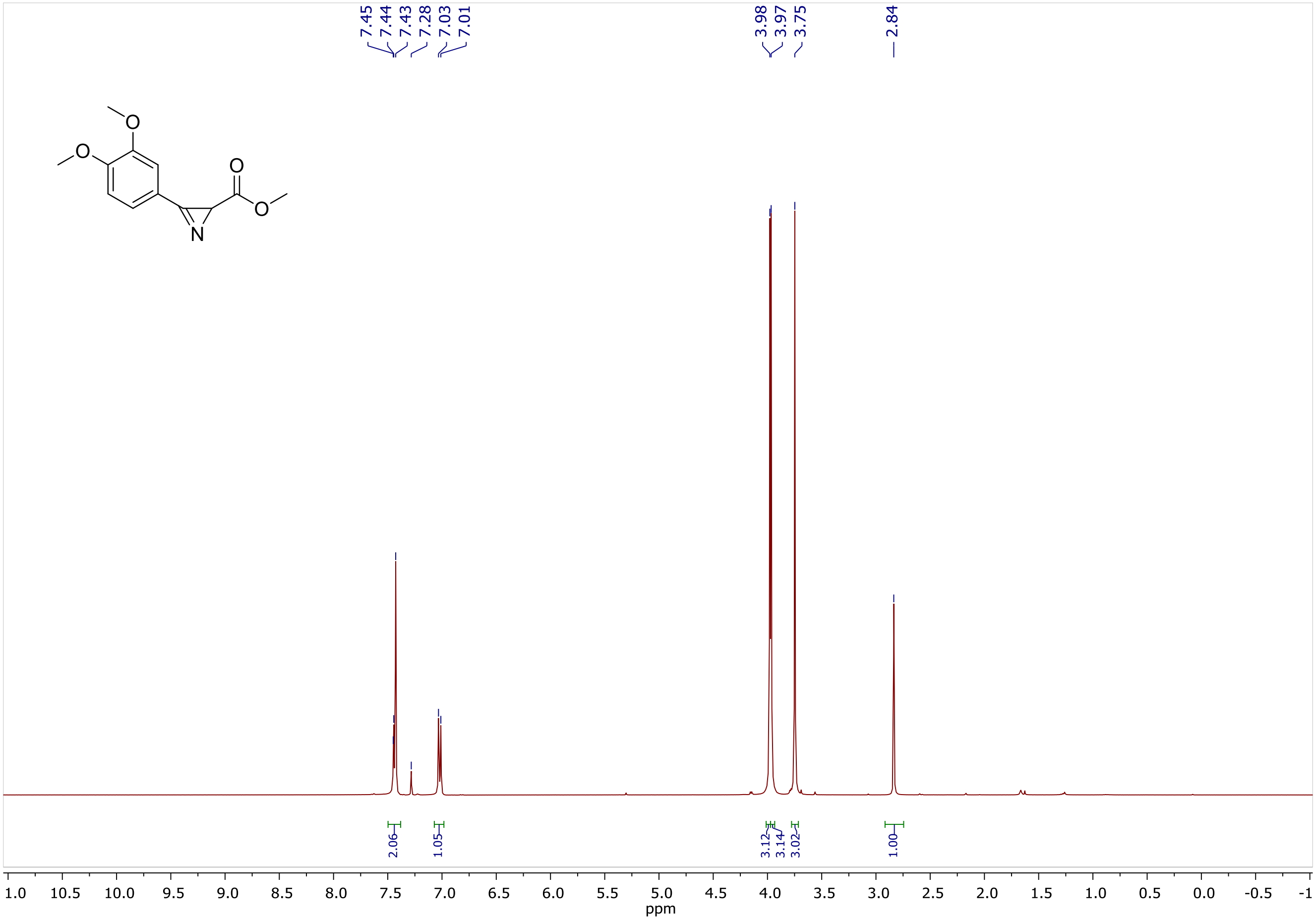

$^{13}C\{^{1}H\}$ NMR (100 MHz, $CDCl_3$) spectrum of methyl 3-(3,4-dimethoxyphenyl)-*2H*-azirine-2-carboxylate (**1c**)

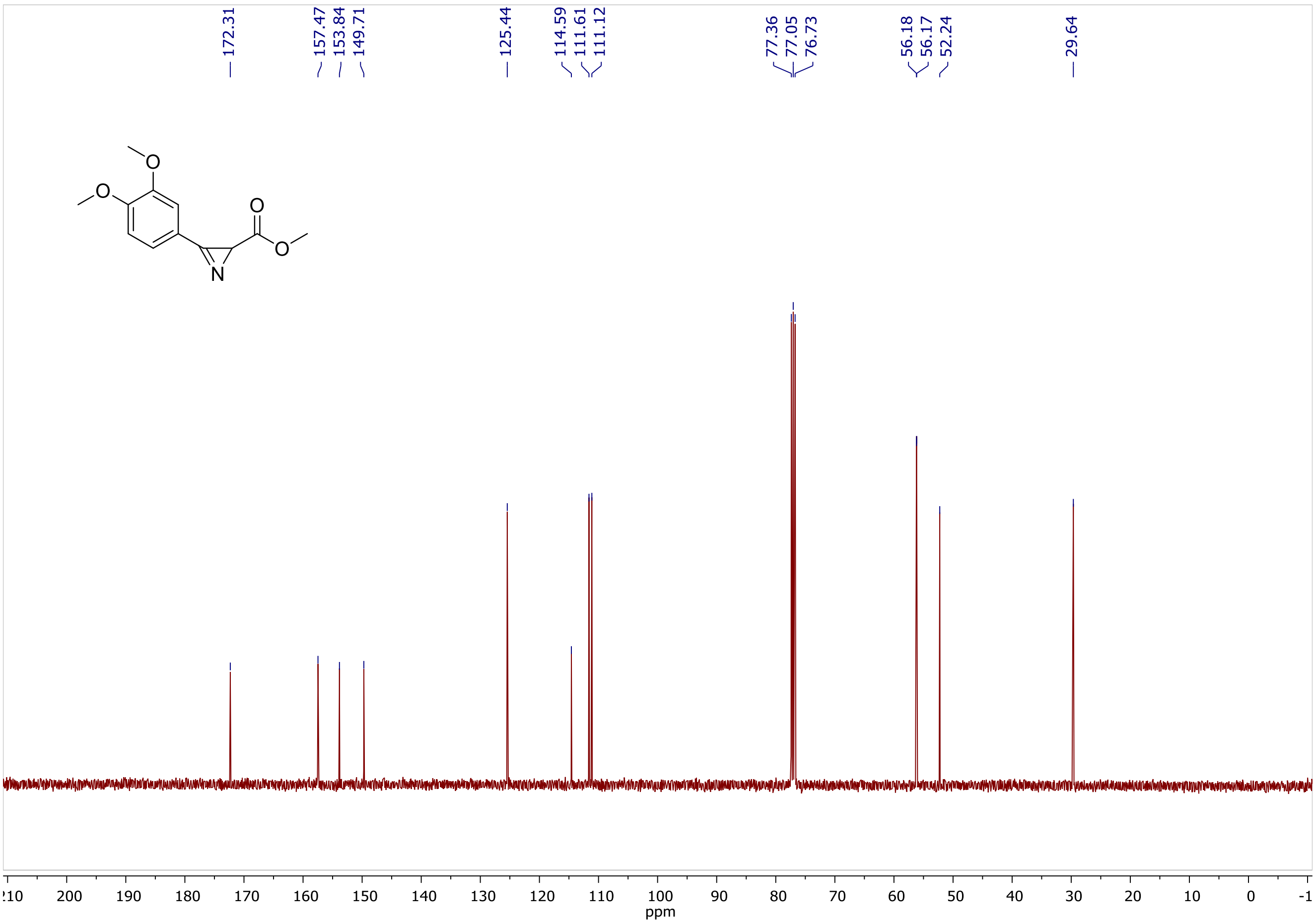

^{1}H NMR (400 MHz, $CDCl_3$) spectrum of methyl 3-(4-chlorophenyl)-*2H*-azirine-2-carboxylate (**1e**)

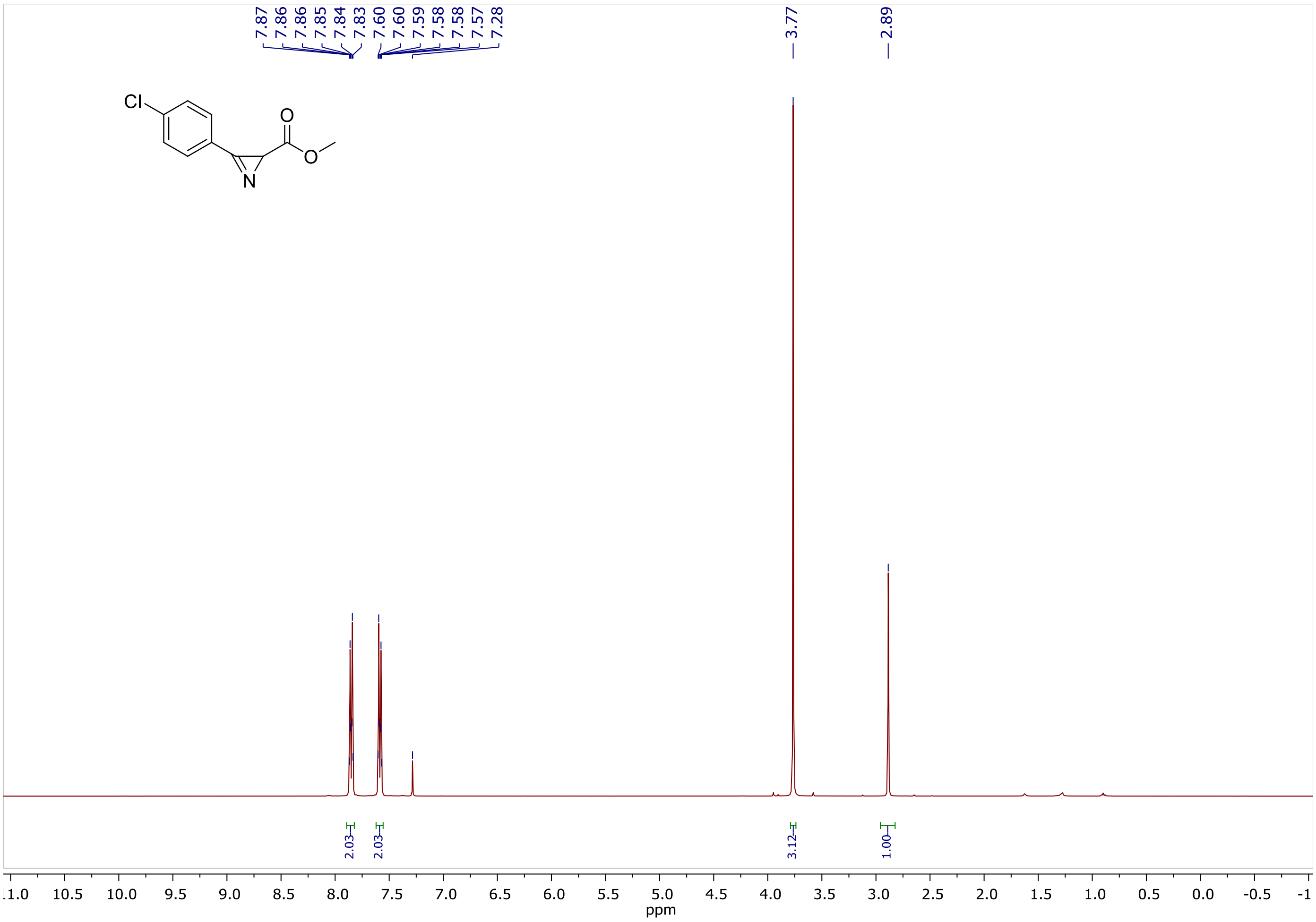

^{1}H NMR (400 MHz, $CDCl_3$) spectrum of methyl 3-(4-(dimethylamino)phenyl)-*2H*-azirine-2-carboxylate (**1f**)

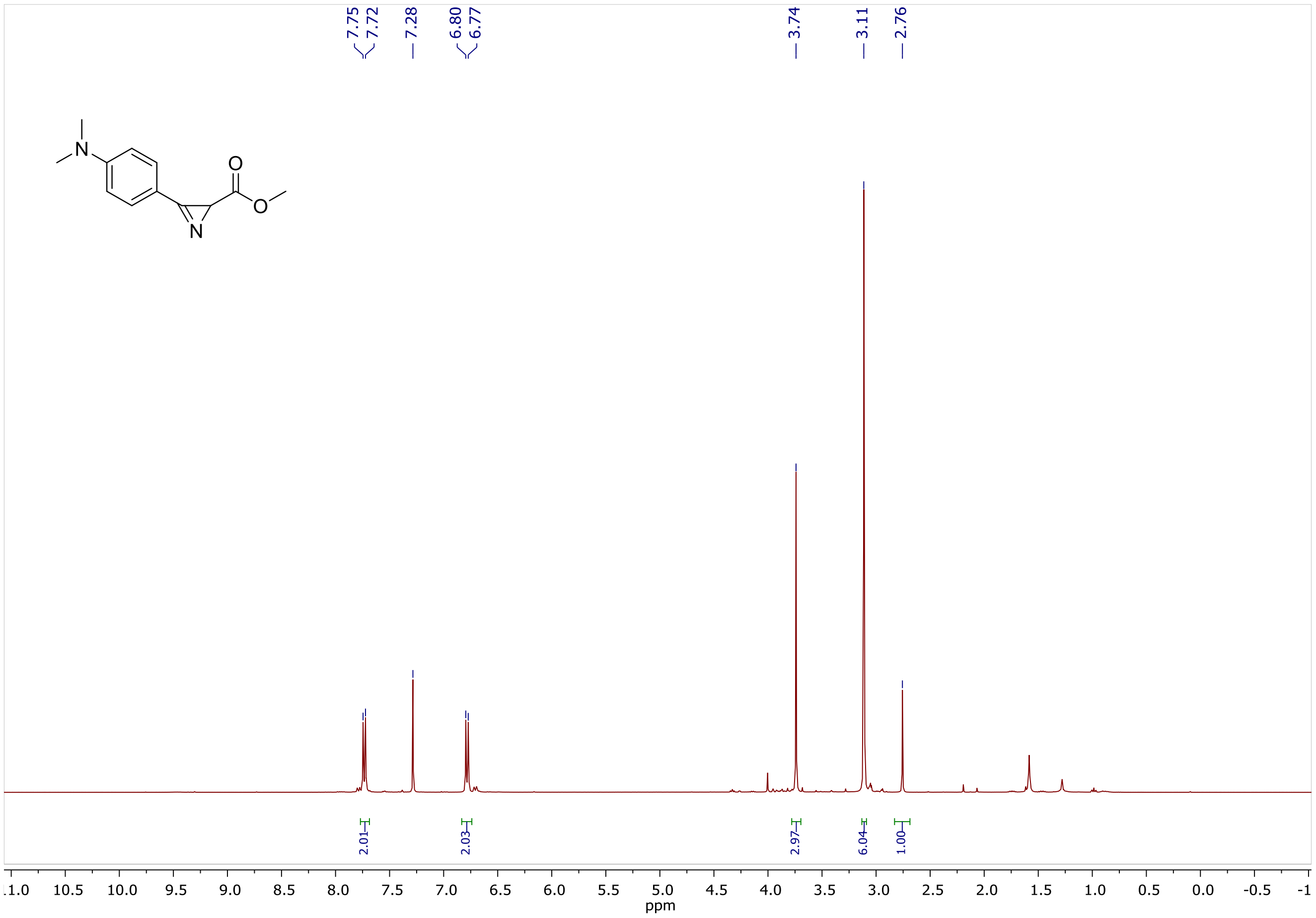

$^{13}C\{^{1}H\}$ NMR (100 MHz, $CDCl_3$) spectrum of methyl 3-(4-(dimethylamino)phenyl)-*2H*-azirine-2-carboxylate (**1f**)

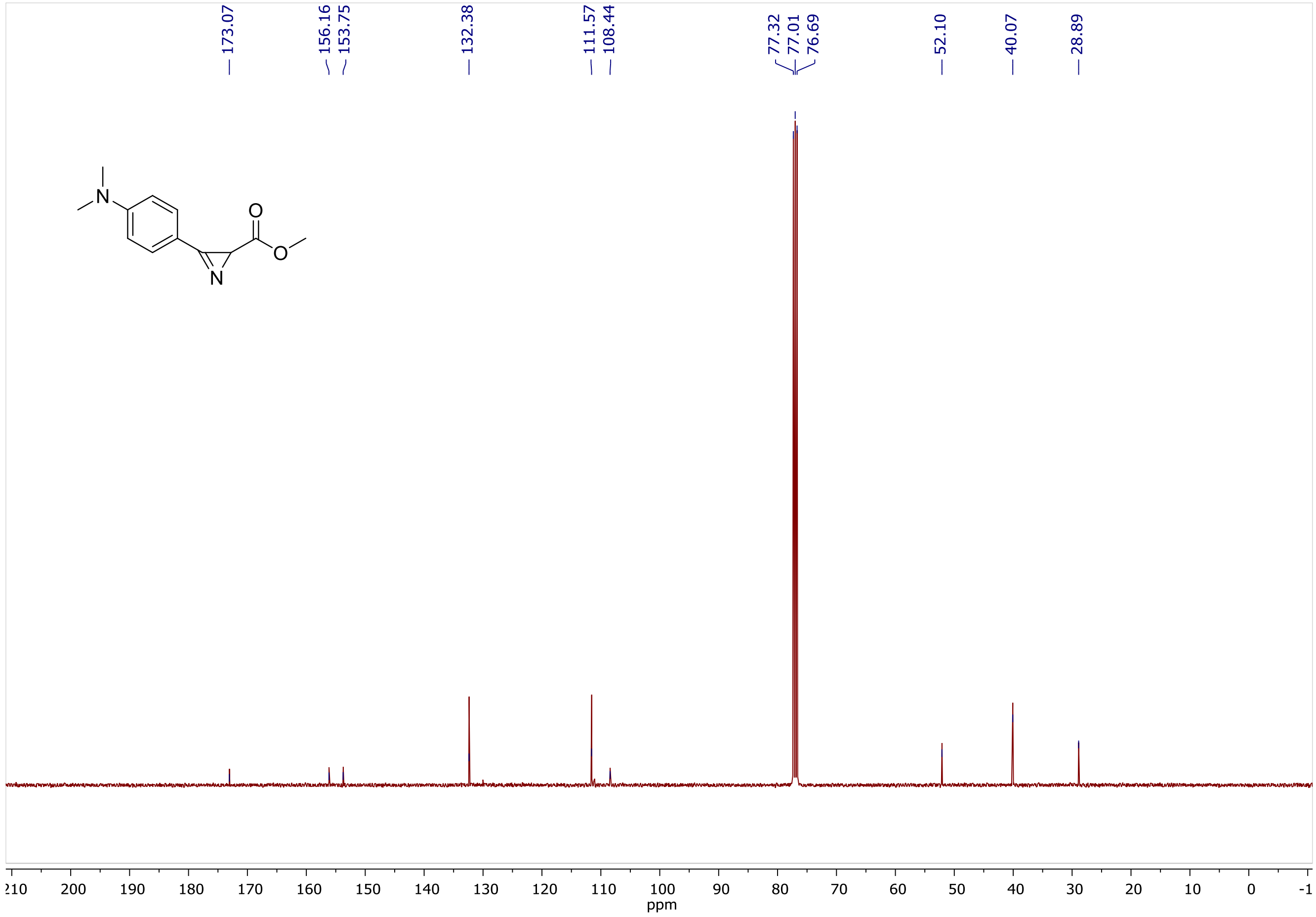

^{1}H NMR (400 MHz, $CDCl_3$) spectrum of methyl 3-(naphthalen-2-yl)-*2H*-azirine-2-carboxylate (**1g**)

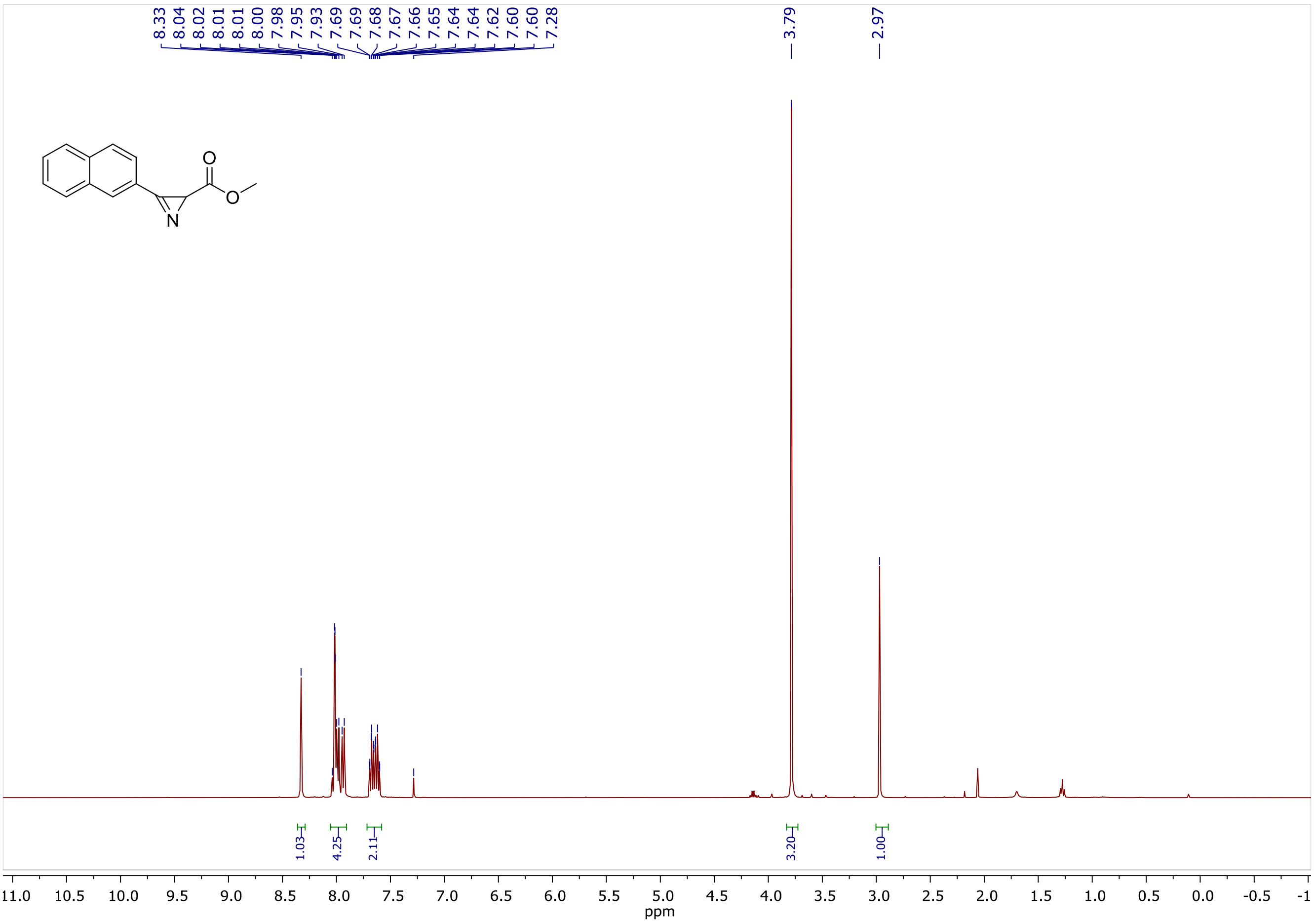

^{13}C{^{1}H} NMR (100 MHz, $CDCl_3$) spectrum of methyl 3-(naphthalen-2-yl)-*2H*-azirine-2-carboxylate (**1g**)

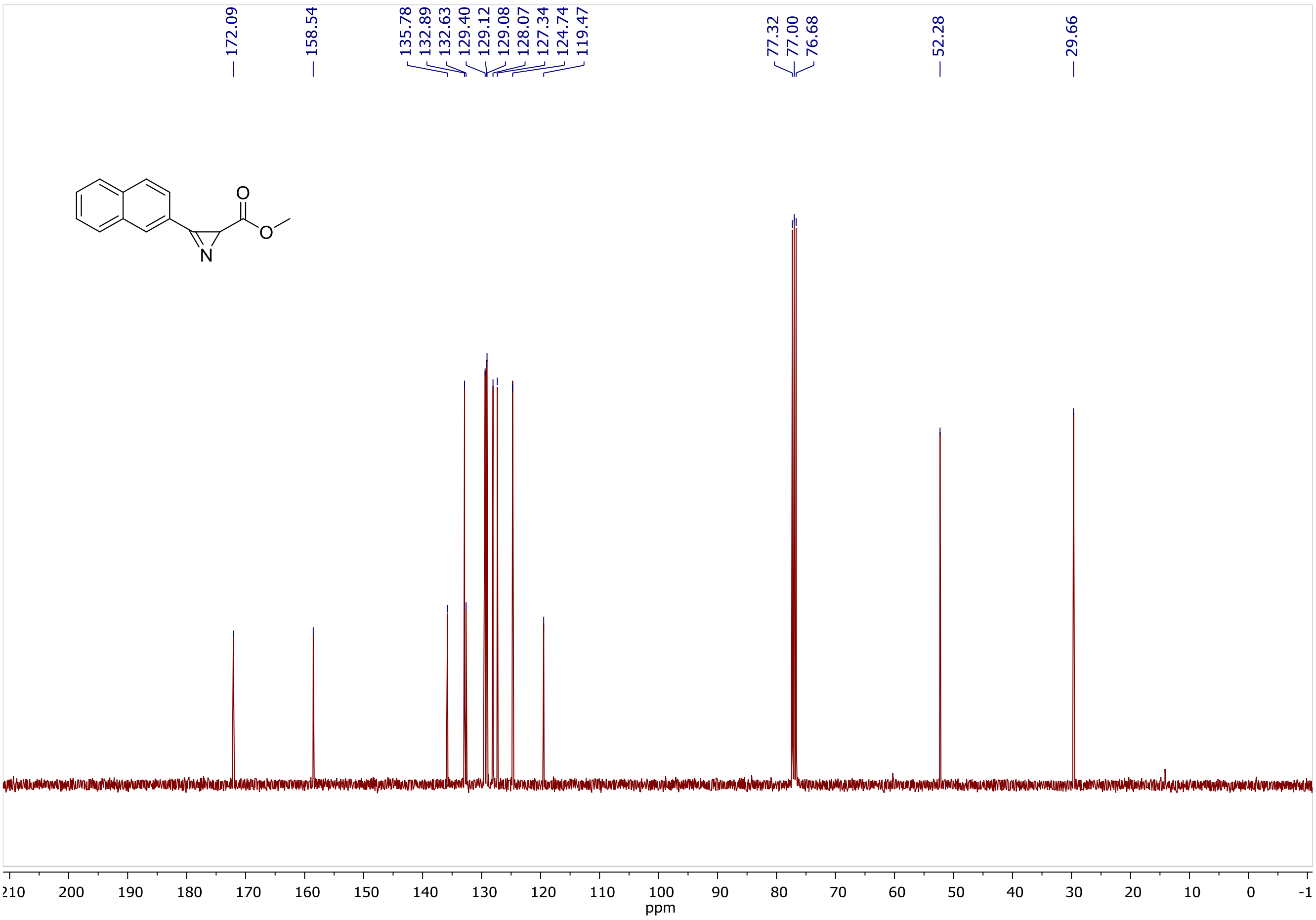

^{1}H NMR (400 MHz, $CDCl_3$) spectrum of methyl 3-(biphenyl-4-yl)-*2H*-azirine-2-carboxylate (**1h**)

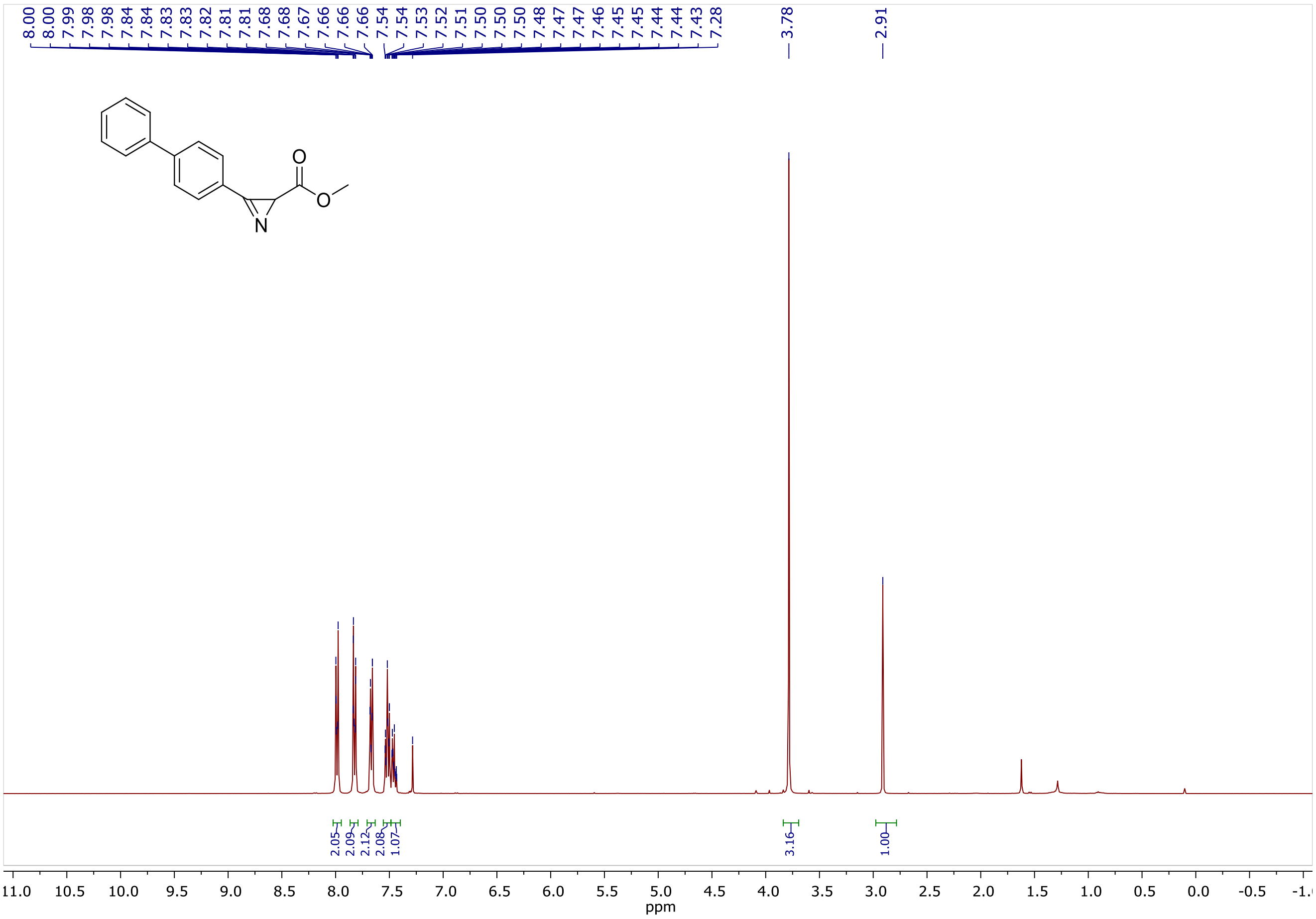

$^{13}C\{^{1}H\}$ NMR (100 MHz, $CDCl_3$) spectrum of methyl 3-(biphenyl-4-yl)-*2H*-azirine-2-carboxylate (**1h**)

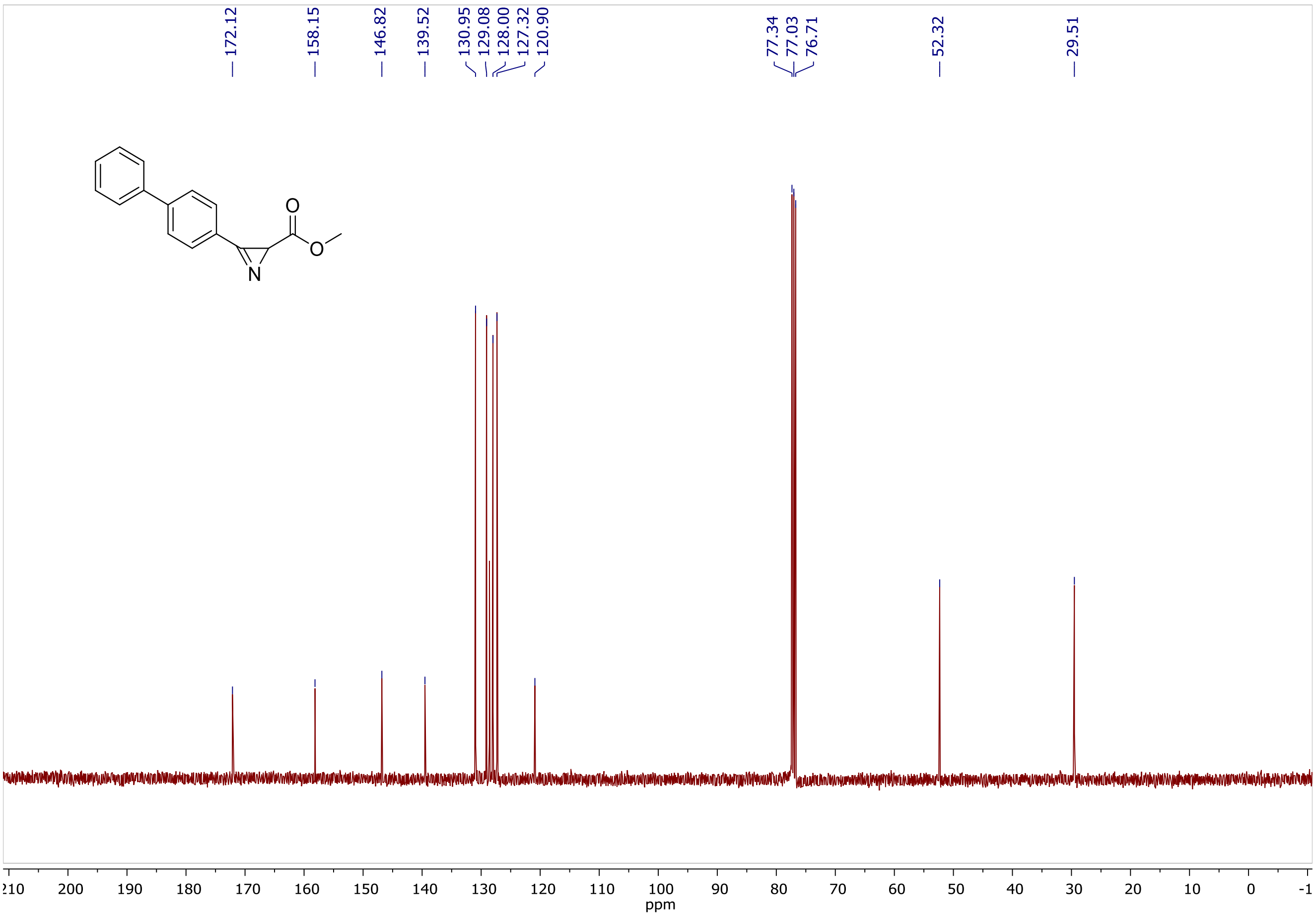

^{1}H NMR (400 MHz, $CDCl_3$) spectrum of methyl 3-(quinolin-2-yl)-*2H*-azirine-2-carboxylate (**1k**)

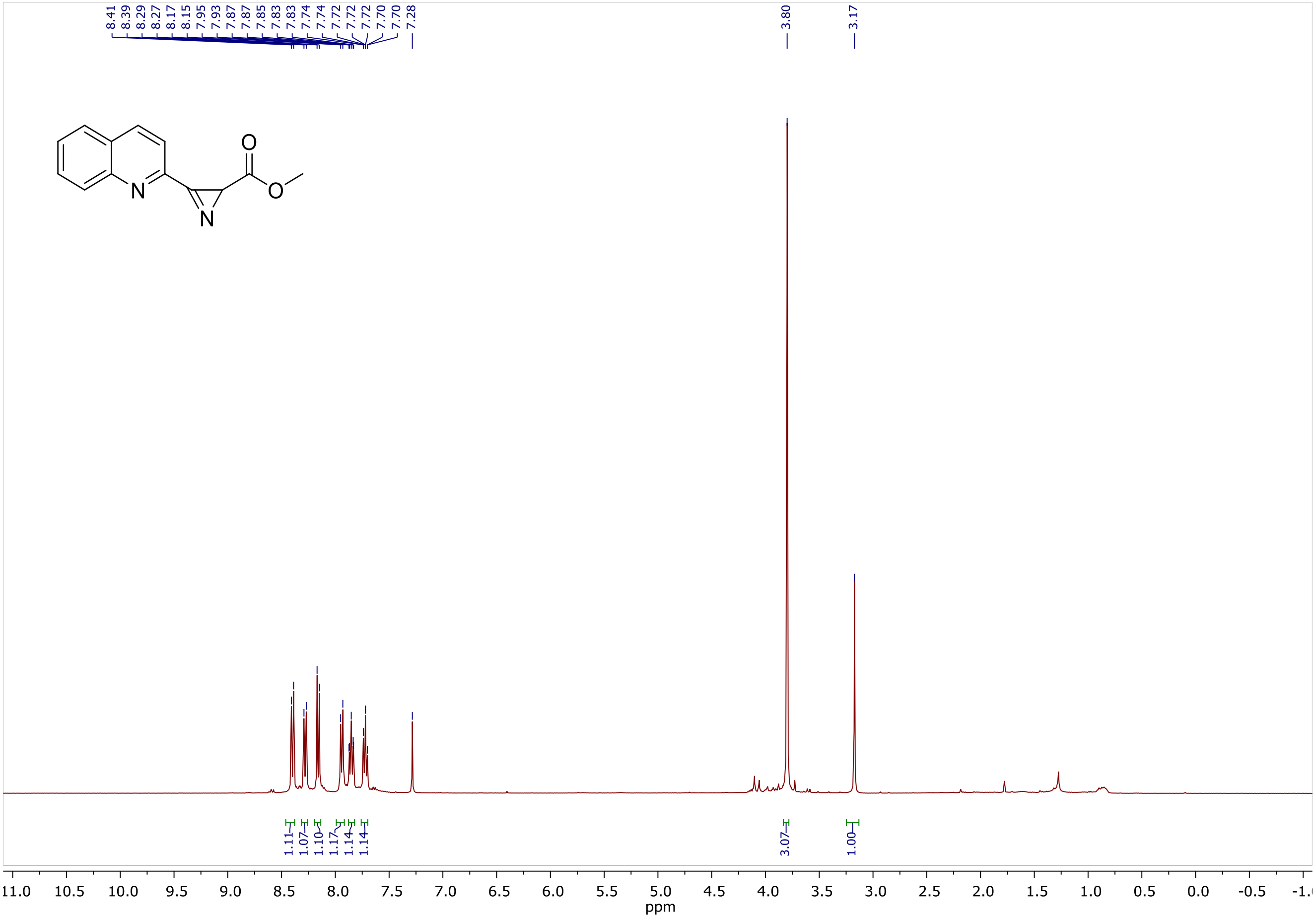

$^{13}C\{^{1}H\}$ NMR (100 MHz, $CDCl_3$) spectrum of methyl 3-(quinolin-2-yl)-*2H*-azirine-2-carboxylate (**1k**)

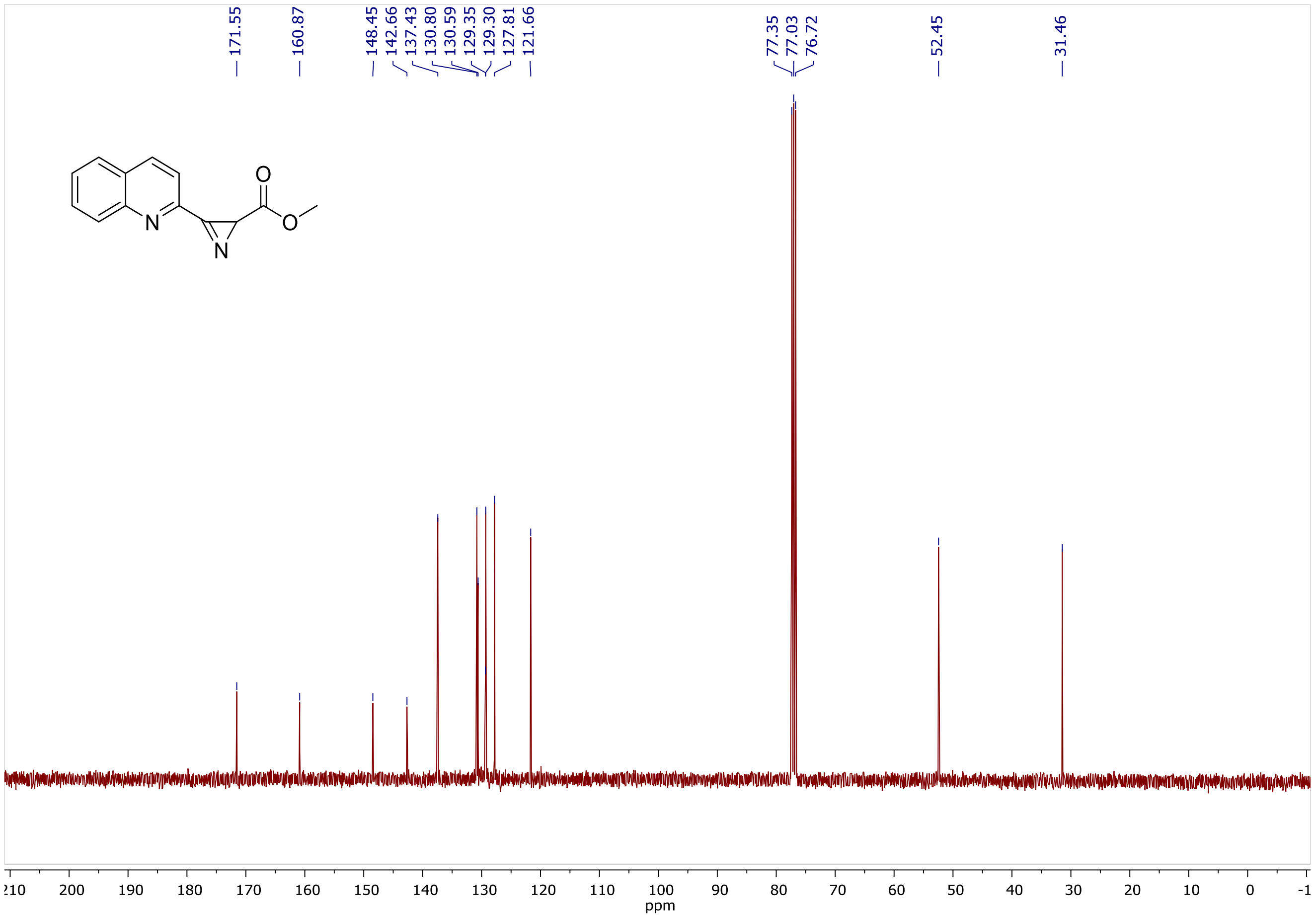

^{1}H NMR (400 MHz, $CDCl_3$) spectrum of dimethyl 2,5-diphenyl-1,6-dihydropyrimidine-4,6-dicarboxylate (**2a**)

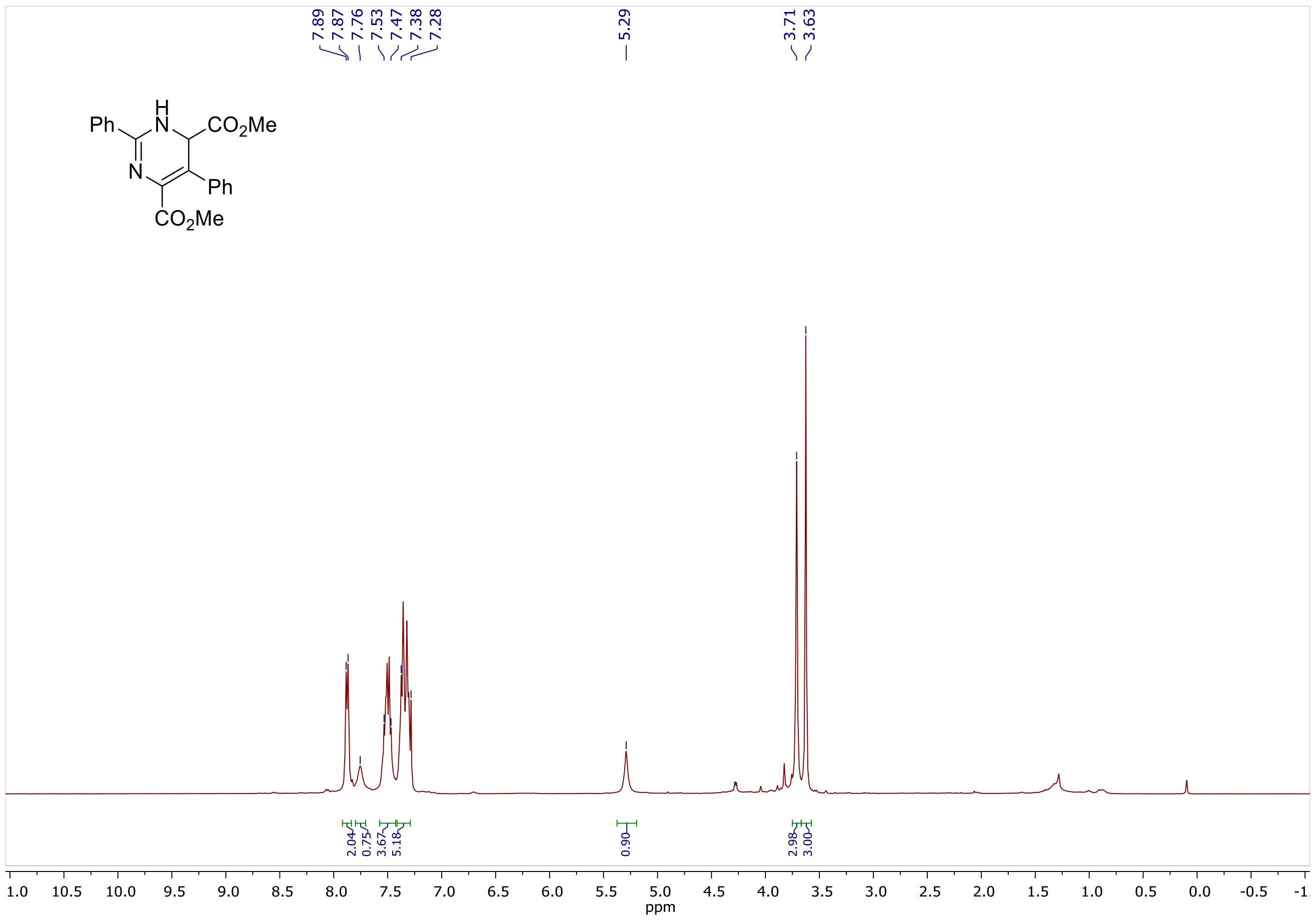

$^{13}C\{^{1}H\}$ NMR (100 MHz, $CDCl_3$) spectrum of dimethyl 2,5-diphenyl-1,6-dihydropyrimidine-4,6-dicarboxylate (**2a**)

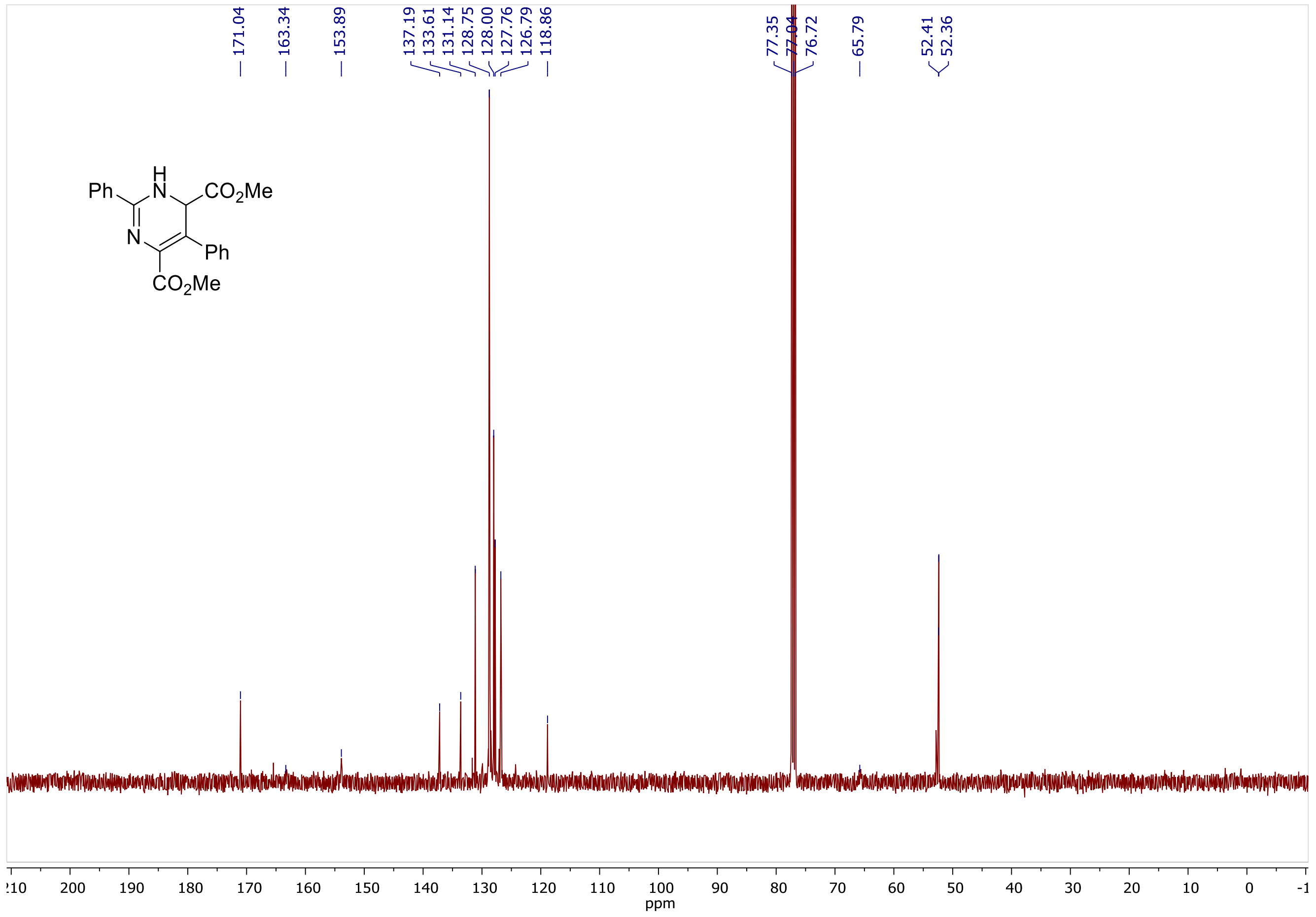

^1H–^{13}C HSQC spectrum (400 MHz, $CDCl_3$) of dimethyl 2,5-diphenyl-1,6-dihydropyrimidine-4,6-dicarboxylate (**2a**)

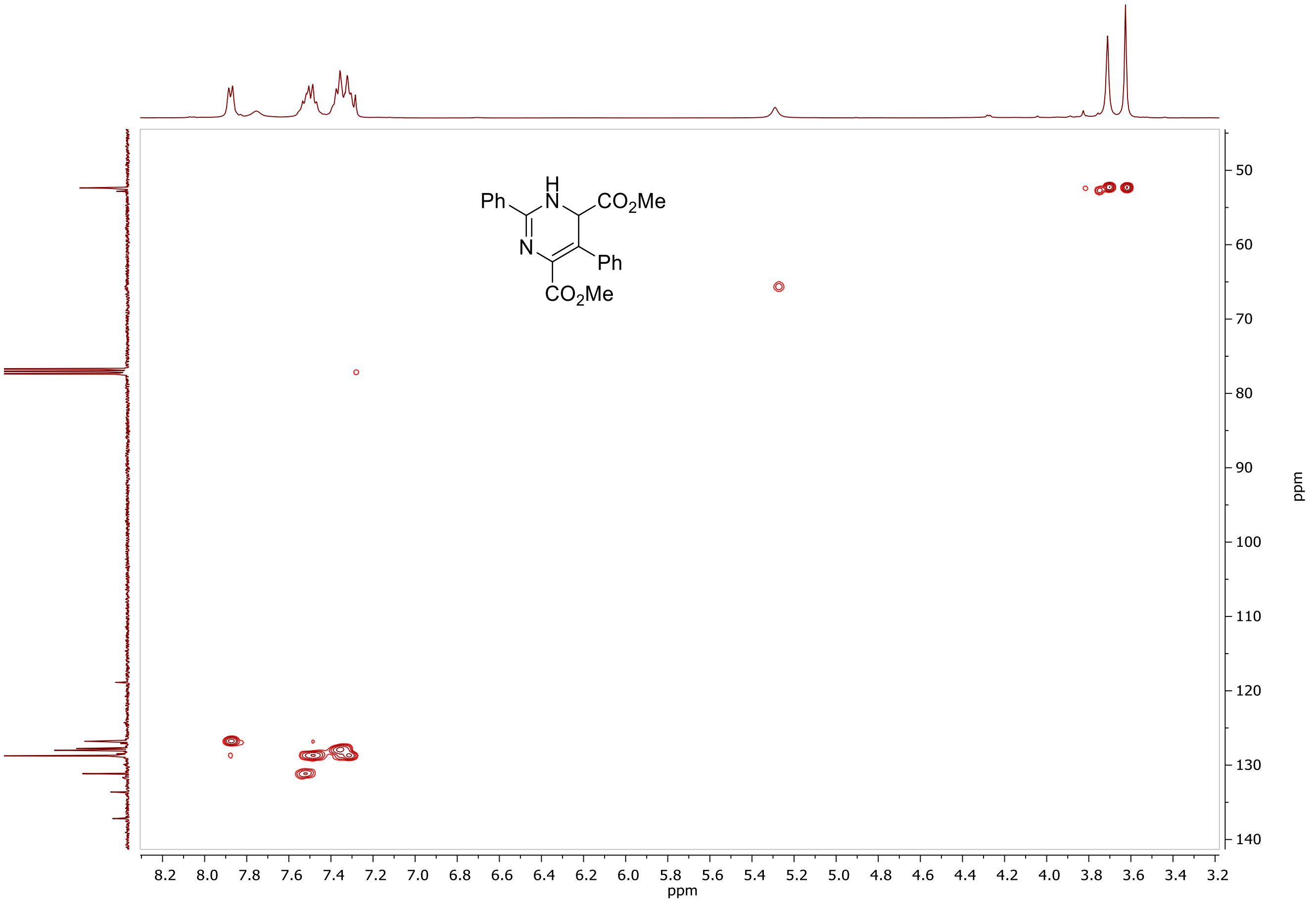

^1H–^{13}C HMBC spectrum (400 MHz, $CDCl_3$) of dimethyl 2,5-diphenyl-1,6-dihydropyrimidine-4,6-dicarboxylate (**2a**)

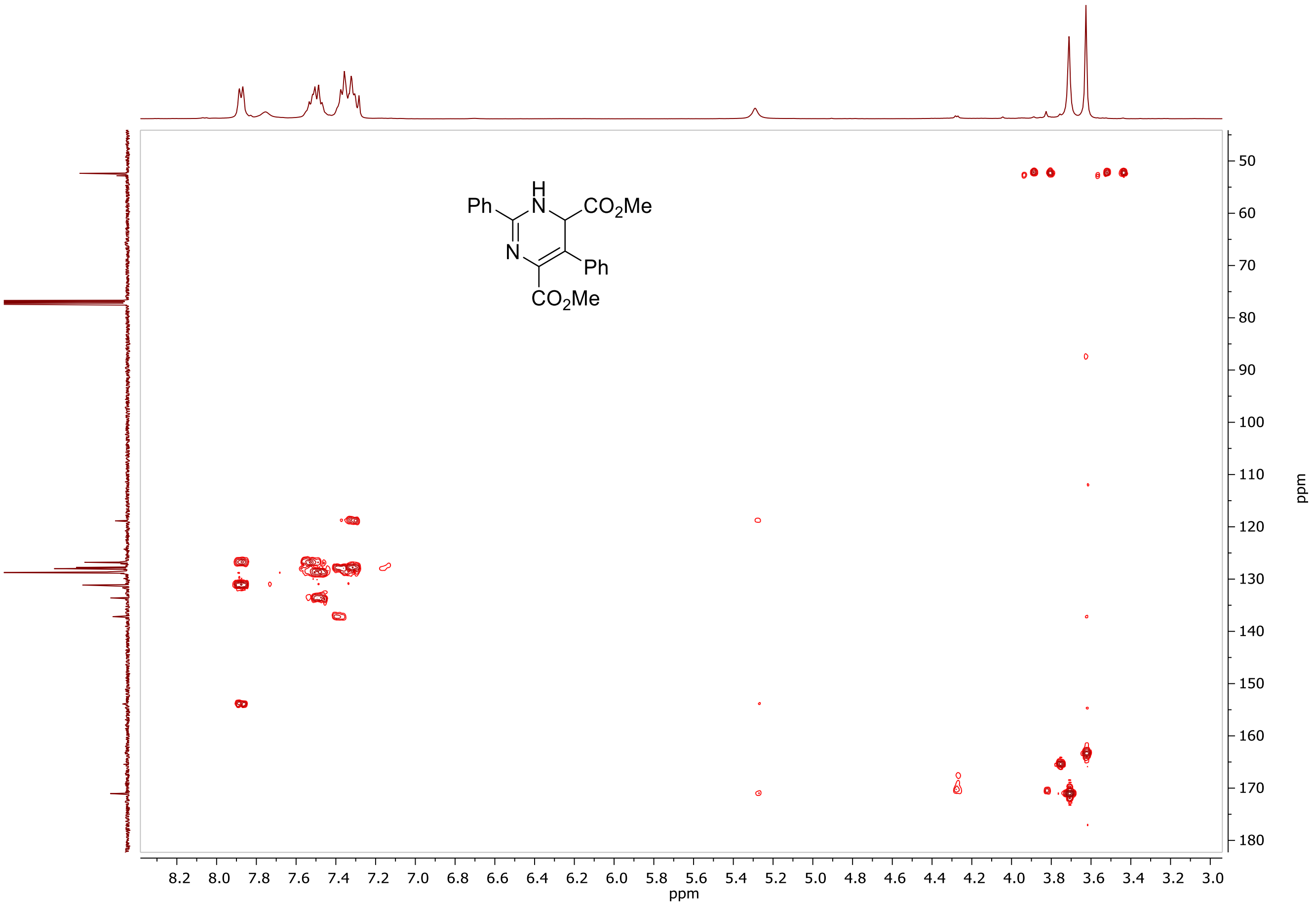

^{1}H NMR (400 MHz, $CDCl_3$) spectrum of dimethyl 2,5-diphenylpyrimidine-4,6-dicarboxylate (**3a**)

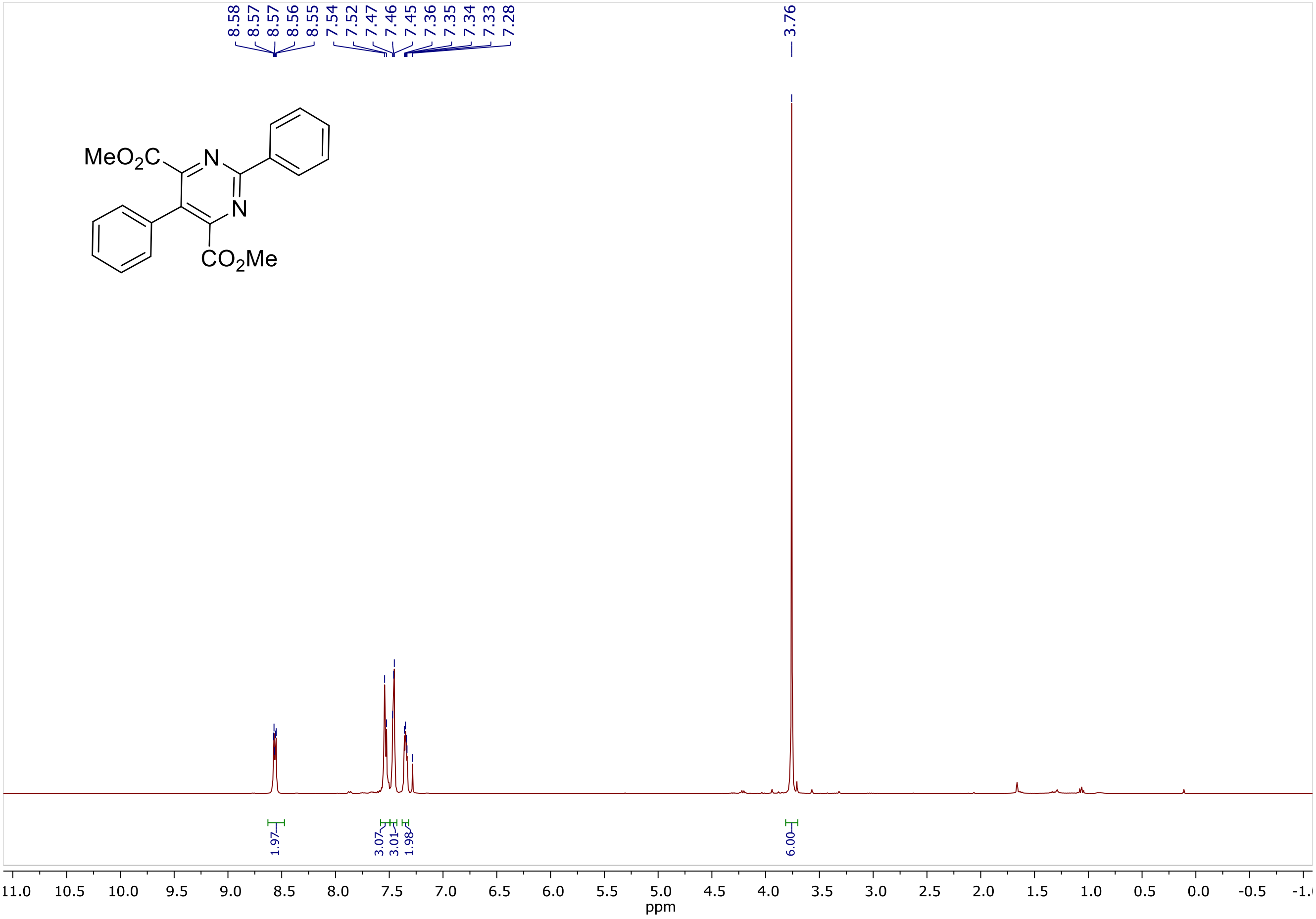

^{13}C{^{1}H} NMR (100 MHz, $CDCl_3$) spectrum of dimethyl 2,5-diphenylpyrimidine-4,6-dicarboxylate (**3a**)

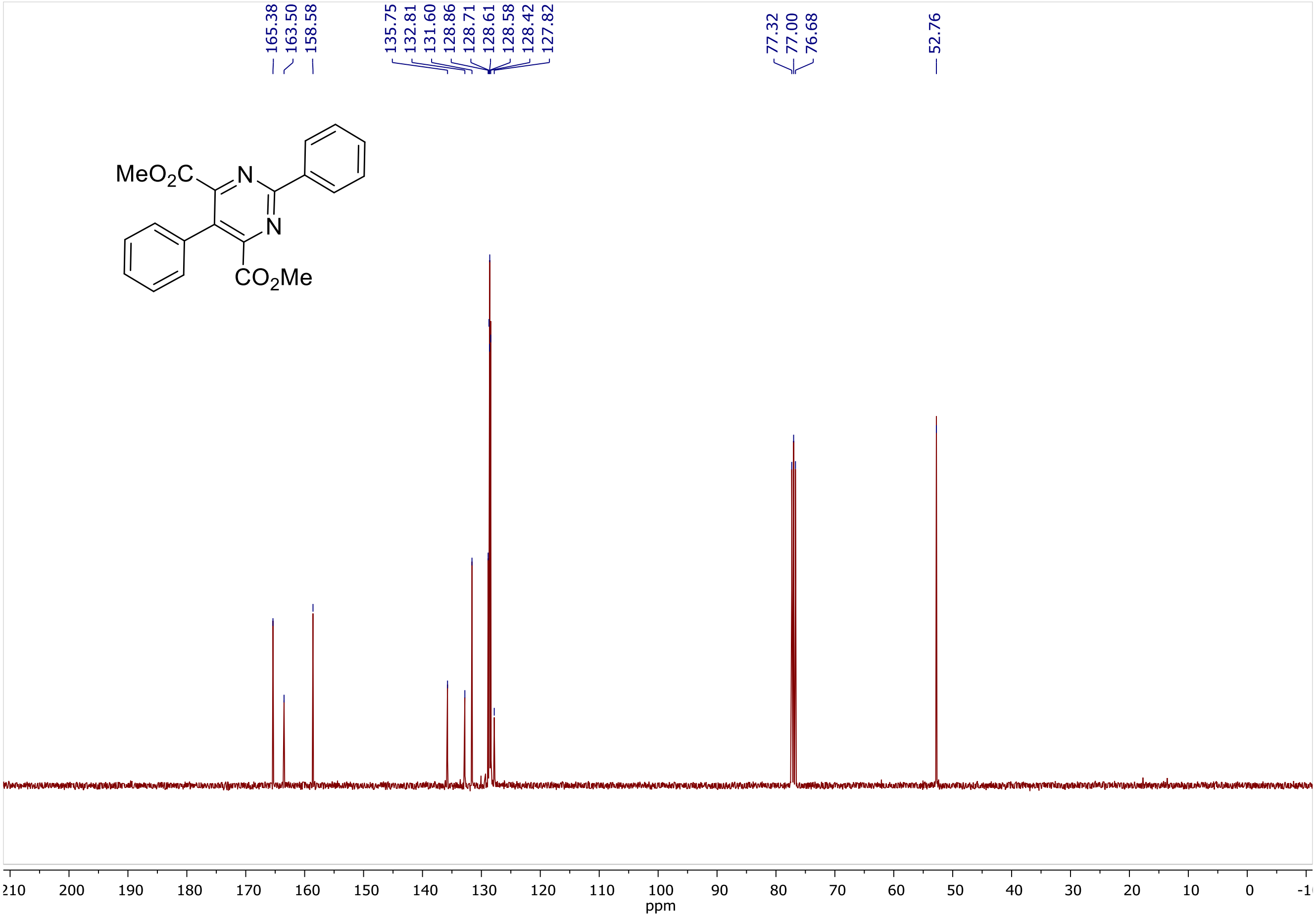

^{1}H–^{13}C HSQC spectrum (400 MHz, $CDCl_3$) of dimethyl 2,5-diphenylpyrimidine-4,6-dicarboxylate (**3a**)

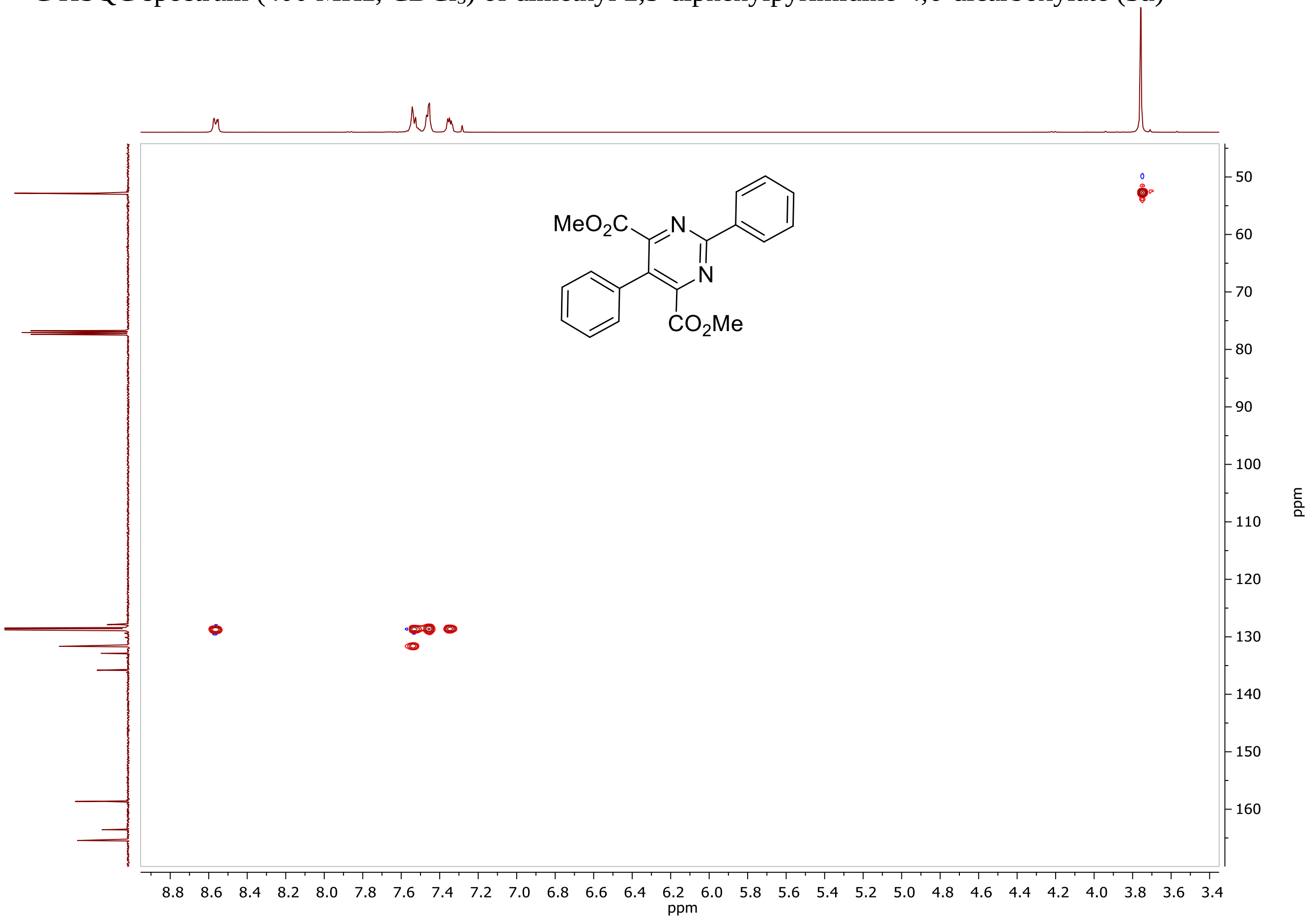

^{1}H–^{13}C HMBC spectrum (400 MHz, $CDCl_3$) of dimethyl 2,5-diphenylpyrimidine-4,6-dicarboxylate (**3a**)

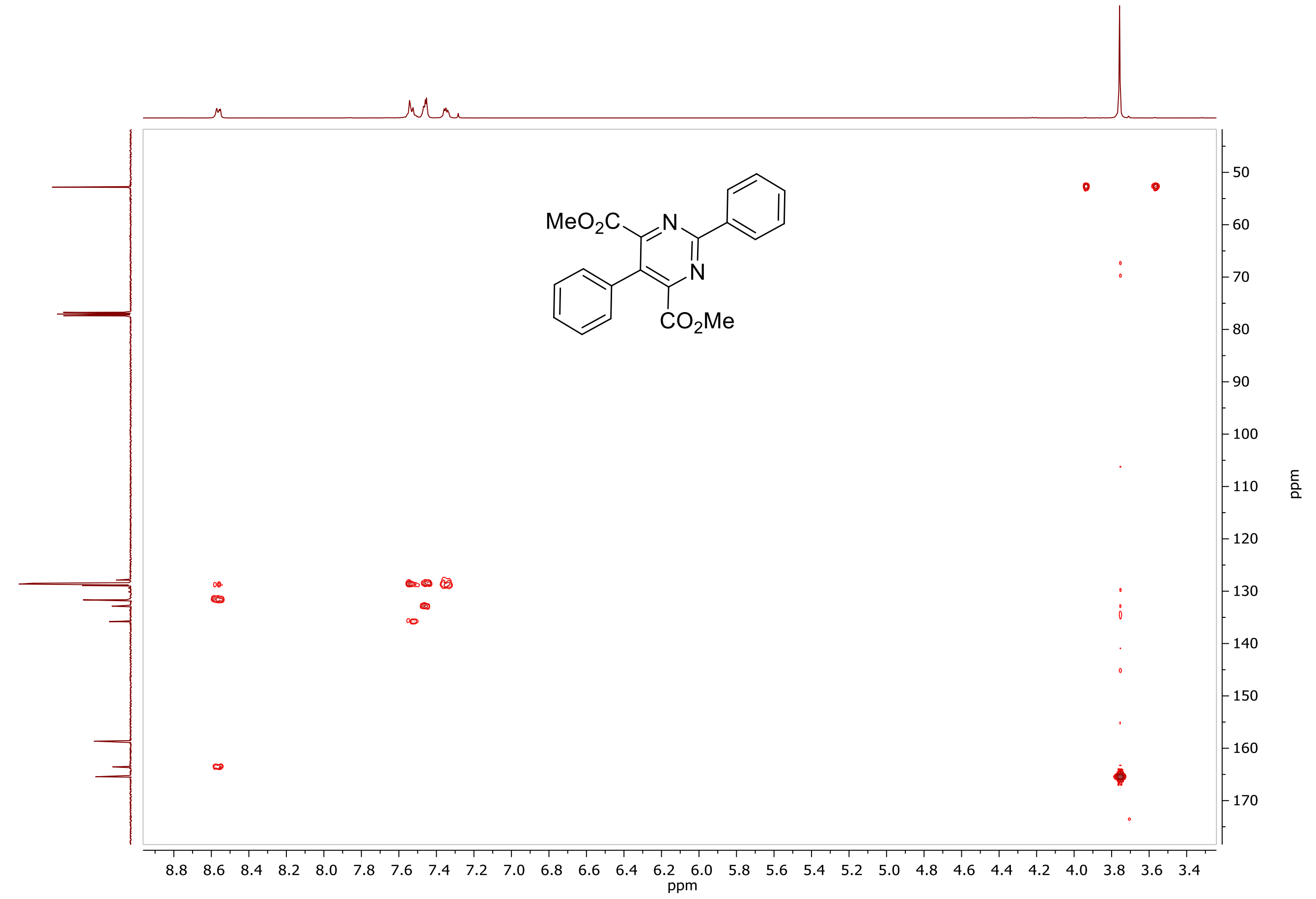

^{1}H NMR (400 MHz, $CDCl_3$) spectrum of dimethyl 2,5-di(*p*-tolyl)pyrimidine-4,6-dicarboxylate (**3b**)

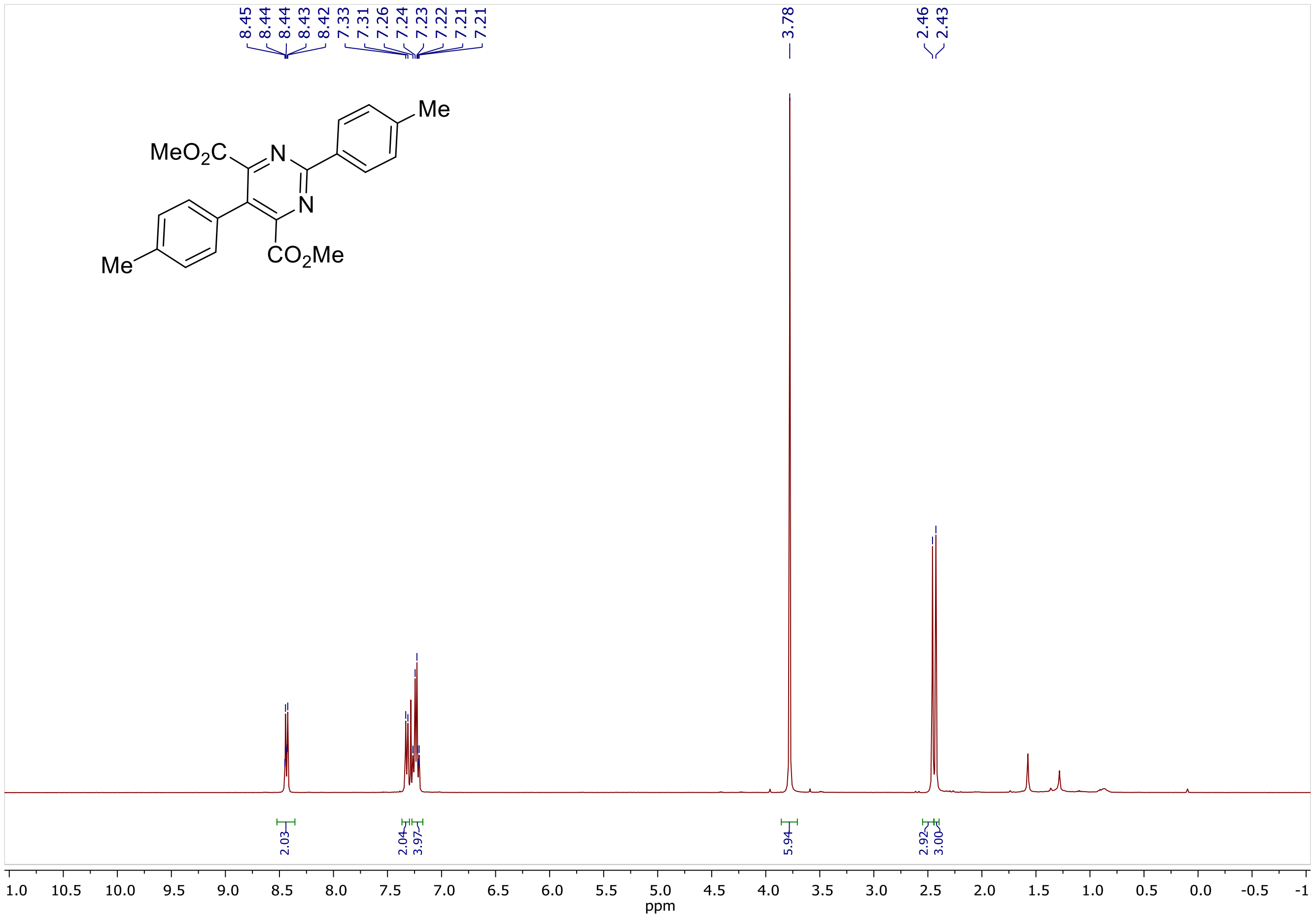

$^{13}C\{^{1}H\}$ NMR (100 MHz, $CDCl_3$) spectrum of dimethyl 2,5-di(*p*-tolyl)pyrimidine-4,6-dicarboxylate (**3b**)

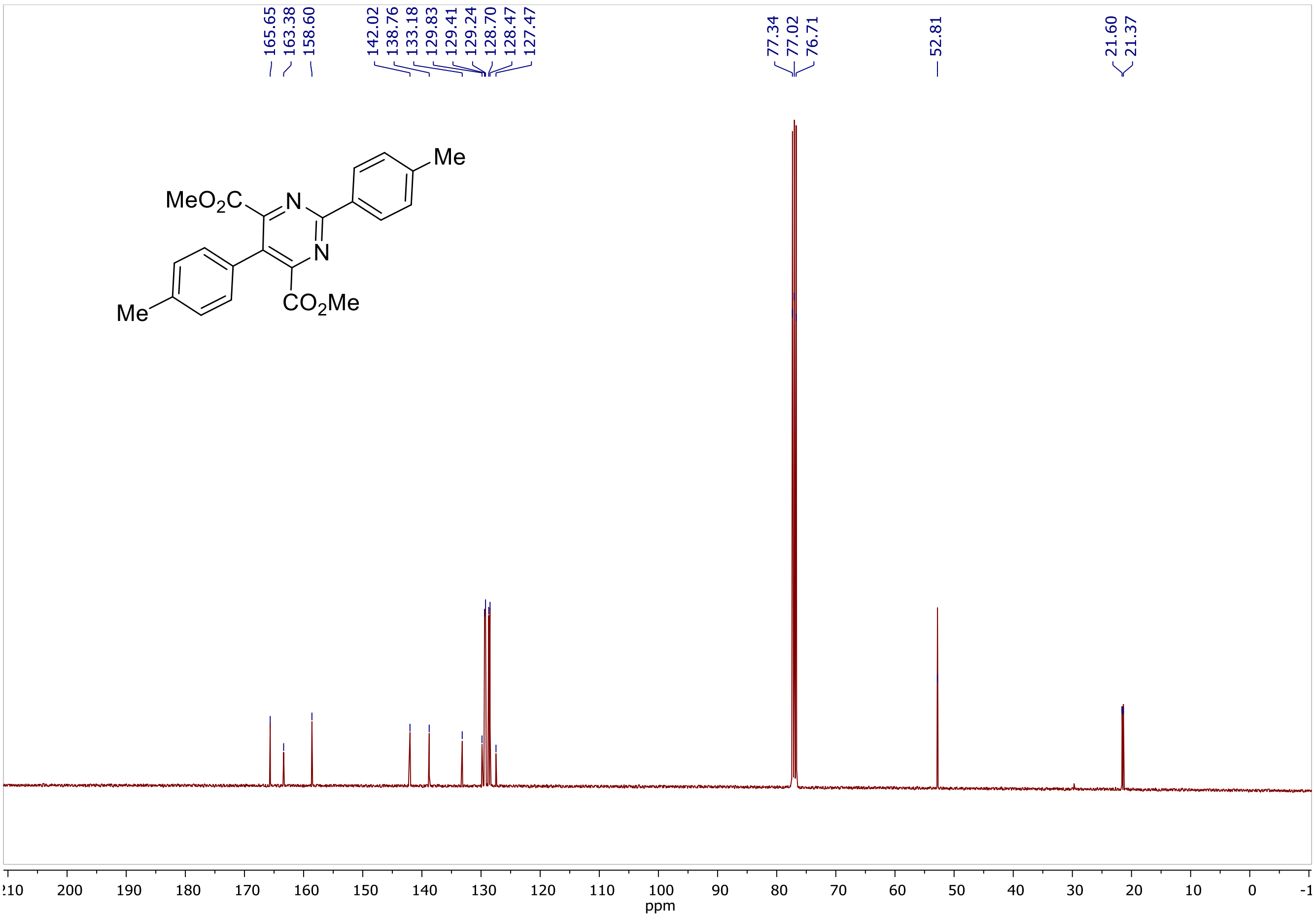

^{1}H NMR (400 MHz, $CDCl_3$) spectrum of dimethyl 2,5-di(3,4-dimethoxyphenyl)pyrimidine-4,6-dicarboxylate (**3c**)

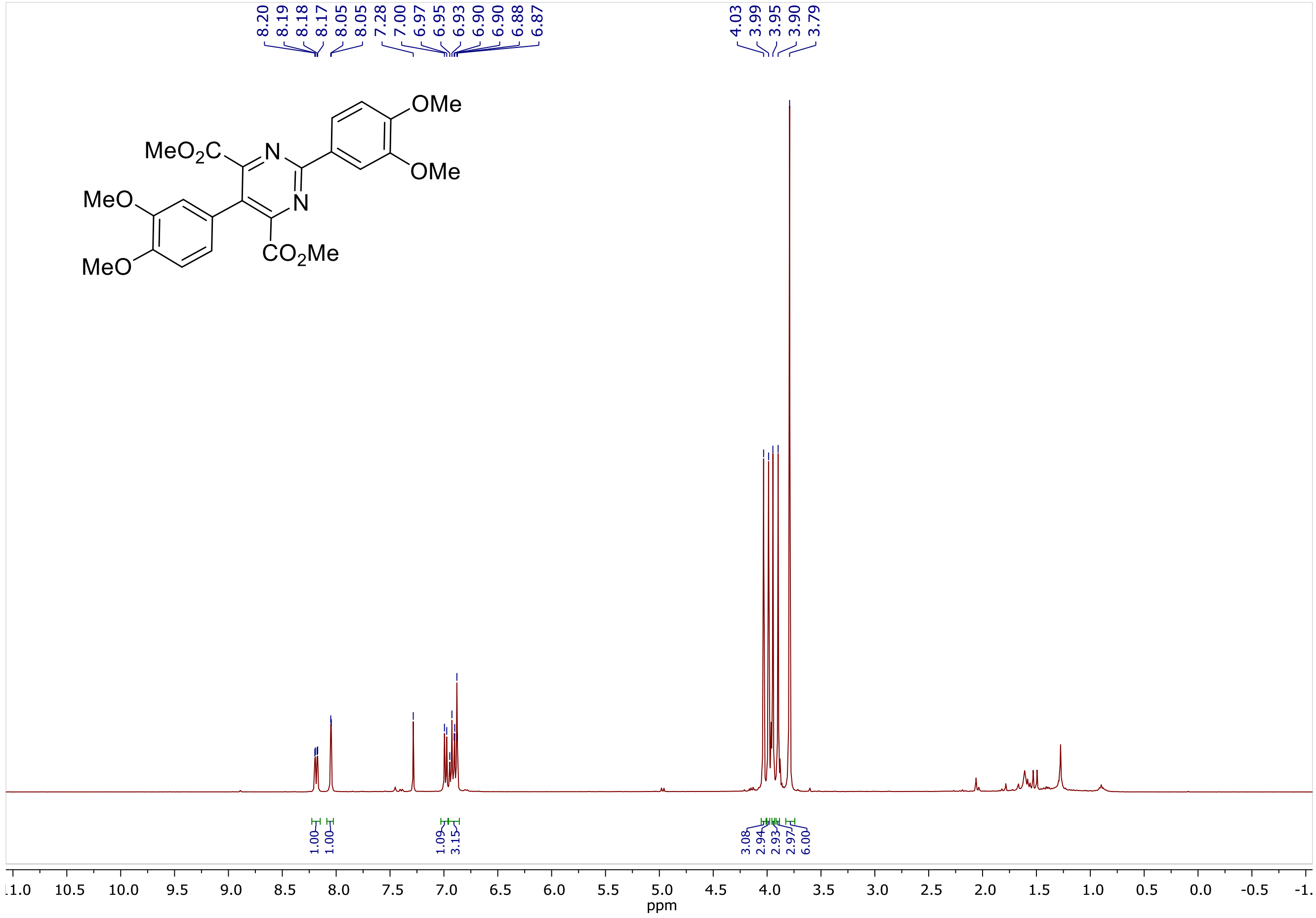

^{13}C{^{1}H} NMR (100 MHz, $CDCl_3$) spectrum of dimethyl 2,5-di(3,4-dimethoxyphenyl)pyrimidine-4,6-dicarboxylate (**3c**)

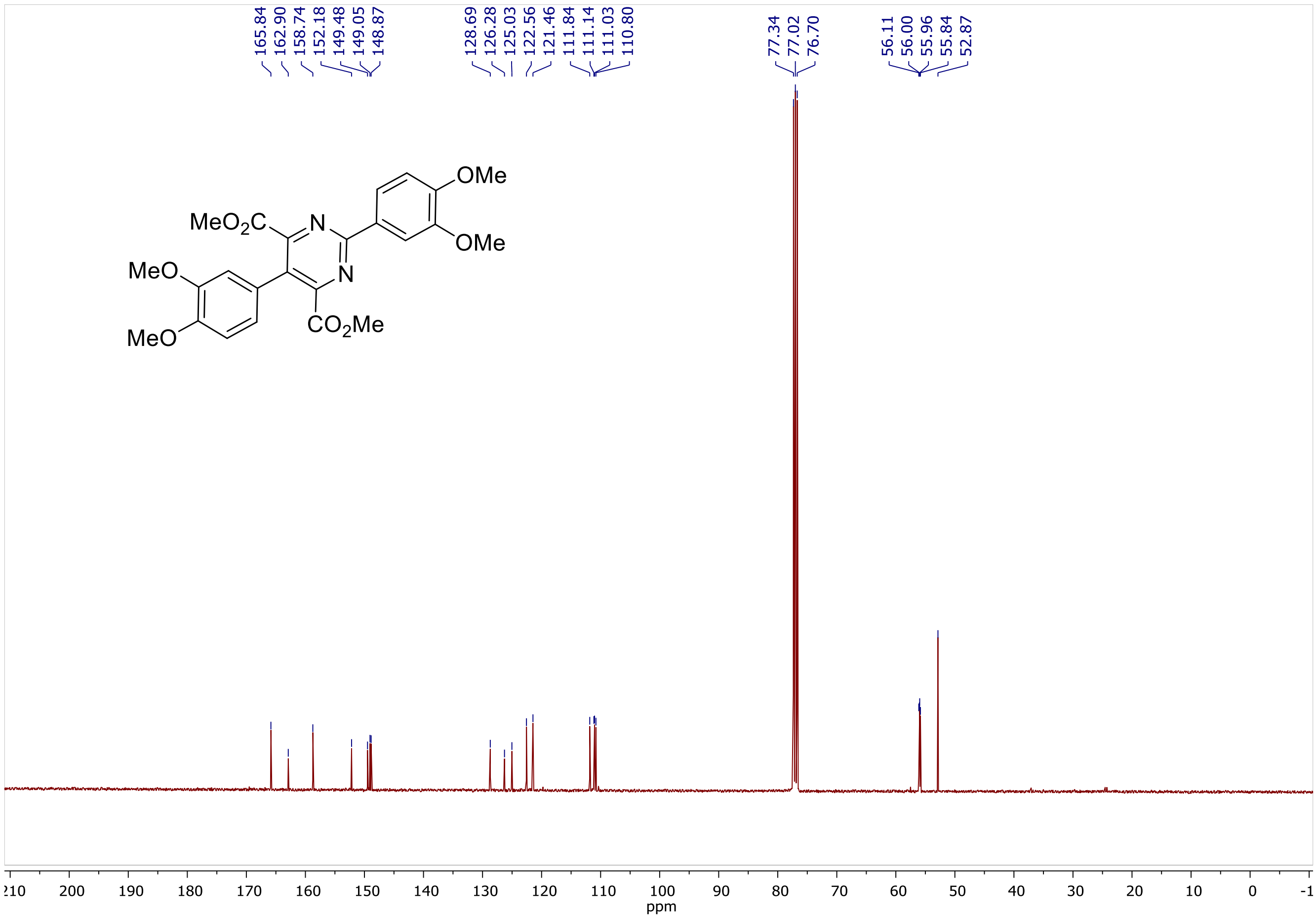

^{1}H NMR (400 MHz, $CDCl_3$) spectrum of dimethyl 2,5-di(3,4-dimethylphenyl)pyrimidine-4,6-dicarboxylate (**3d**)

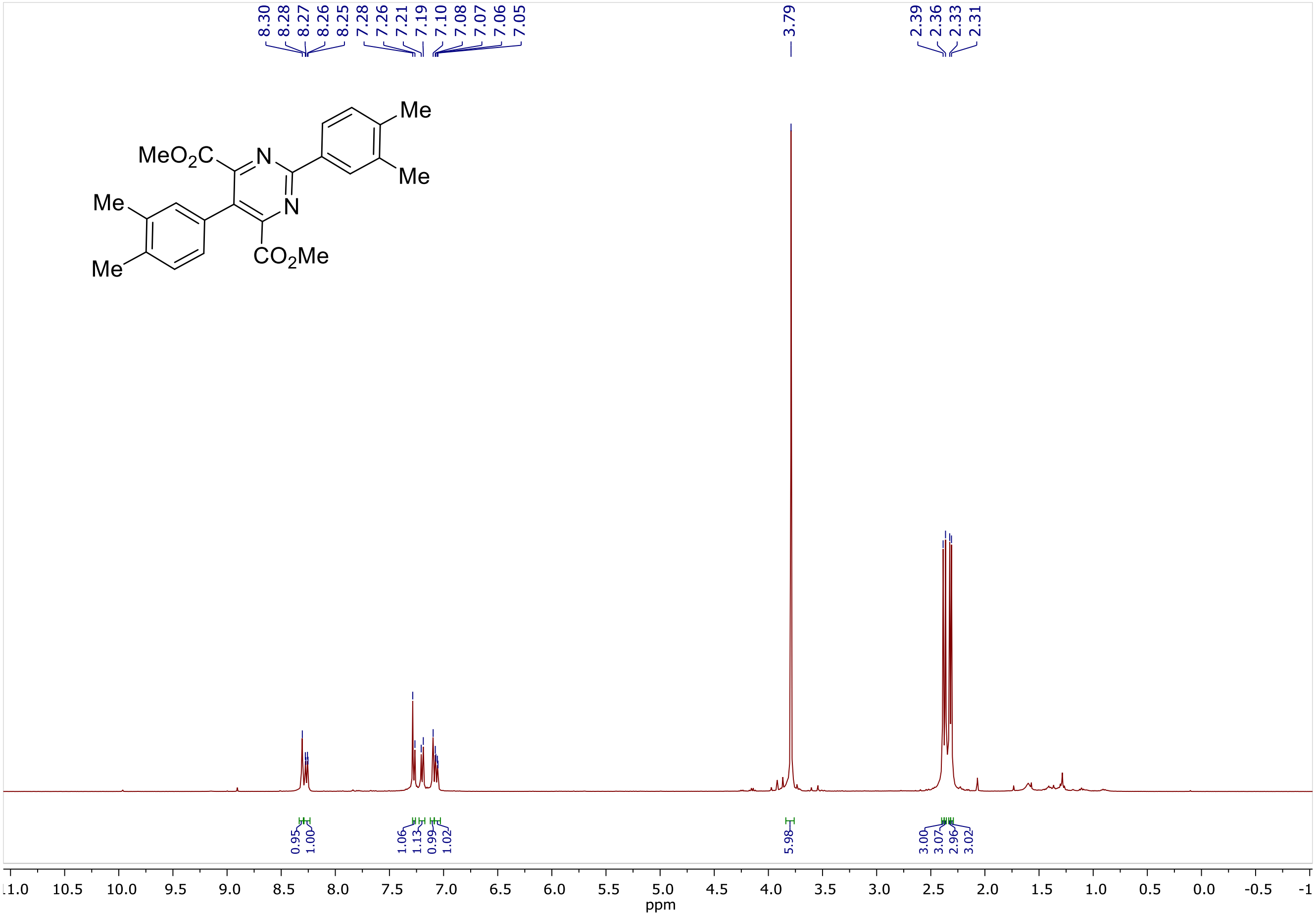

$^{13}C\{^{1}H\}$ NMR (125 MHz, $CDCl_3$) spectrum of dimethyl 2,5-di(3,4-dimethylphenyl)pyrimidine-4,6-dicarboxylate (**3d**)

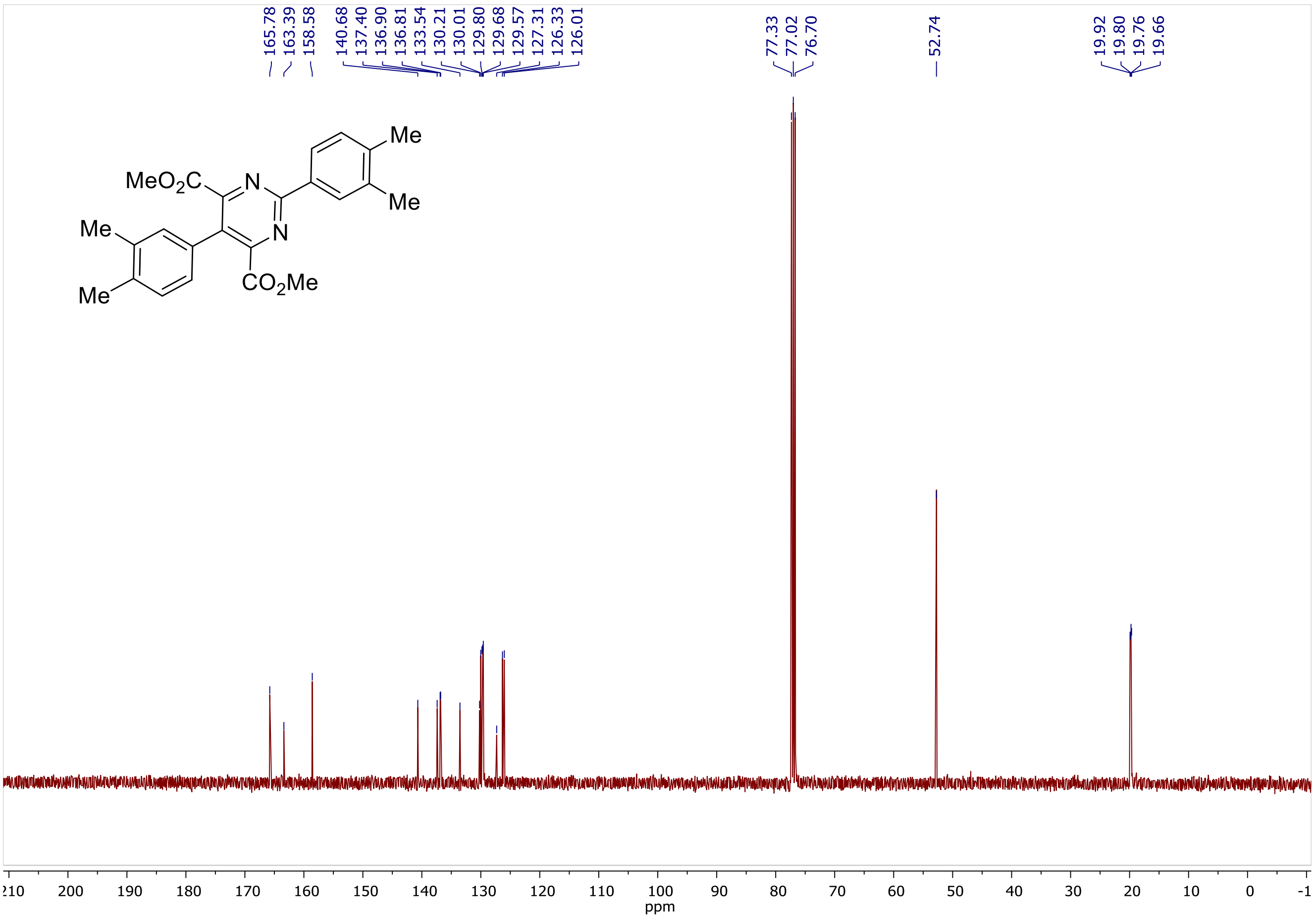

^{1}H NMR (400 MHz, $CDCl_3$) spectrum of dimethyl 2,5-di(4-chlorophenyl)pyrimidine-4,6-dicarboxylate (**3e**)

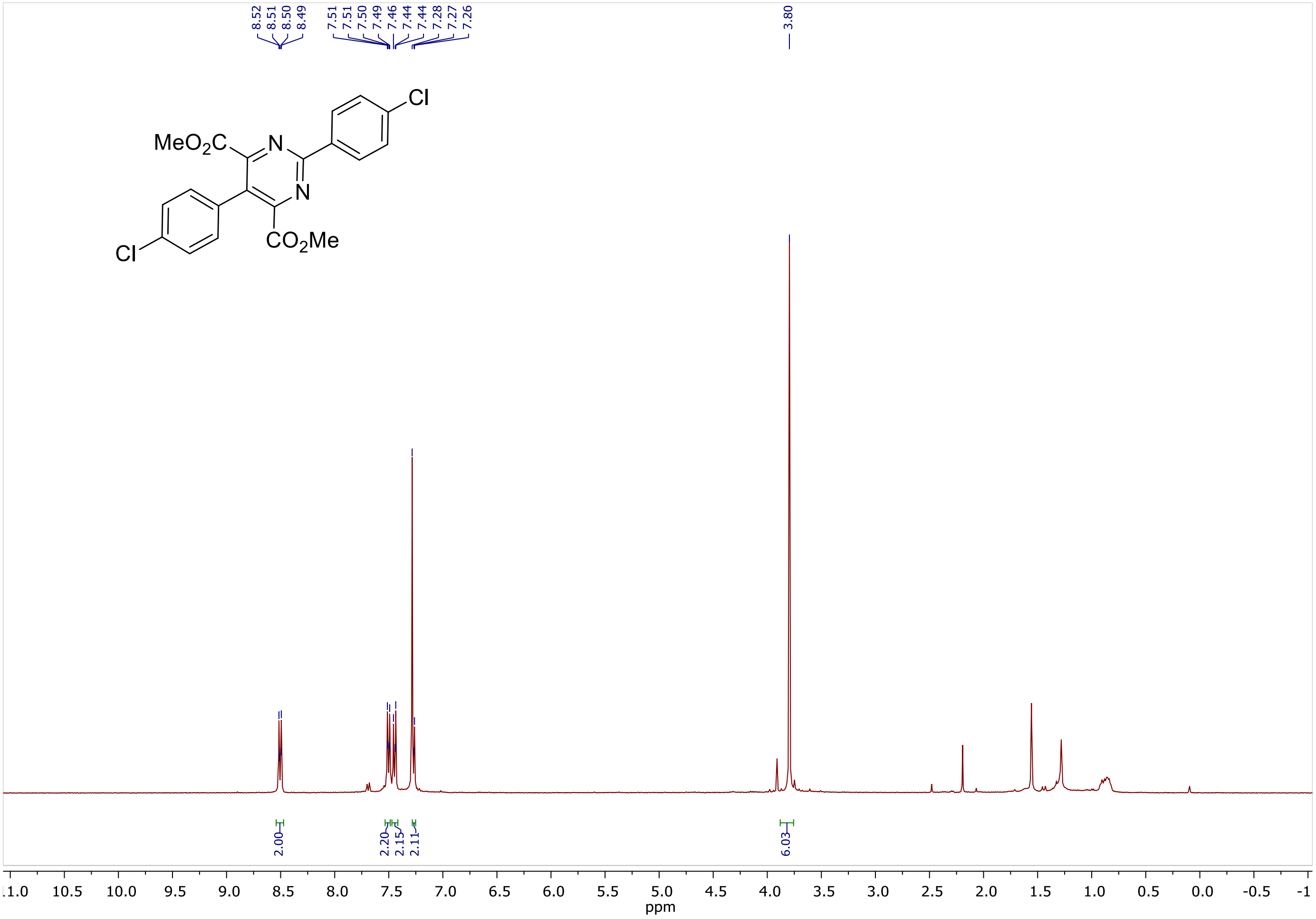

^{1}H NMR (400 MHz, $CDCl_3$) spectrum of dimethyl 2,5-di(4-dimethylaminophenyl)pyrimidine-4,6-dicarboxylate (**3f**)

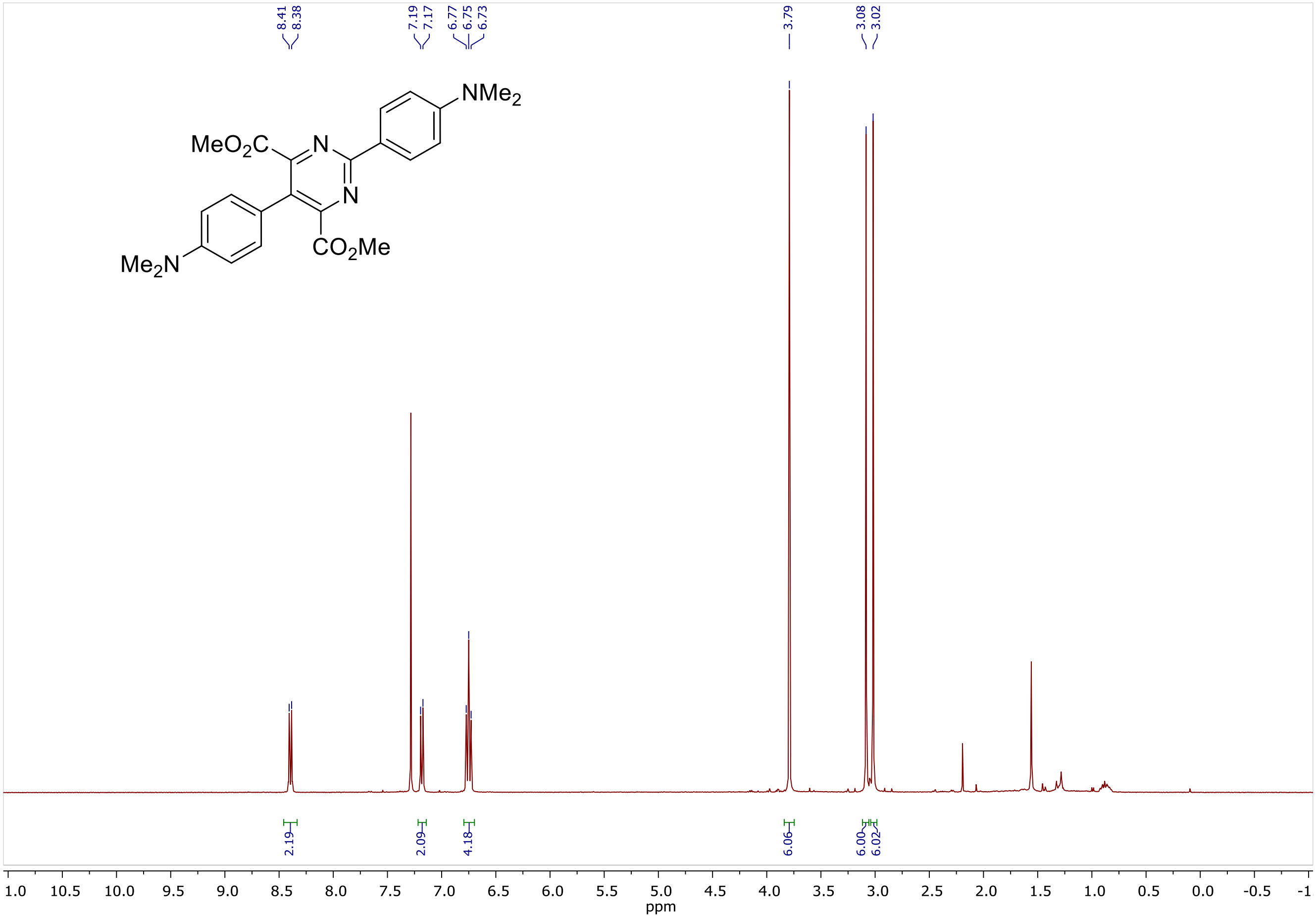

$^{13}C\{^{1}H\}$ NMR (125 MHz, $CDCl_3$) spectrum of dimethyl 2,5-di(4-dimethylaminophenyl)pyrimidine-4,6-dicarboxylate (**3f**)

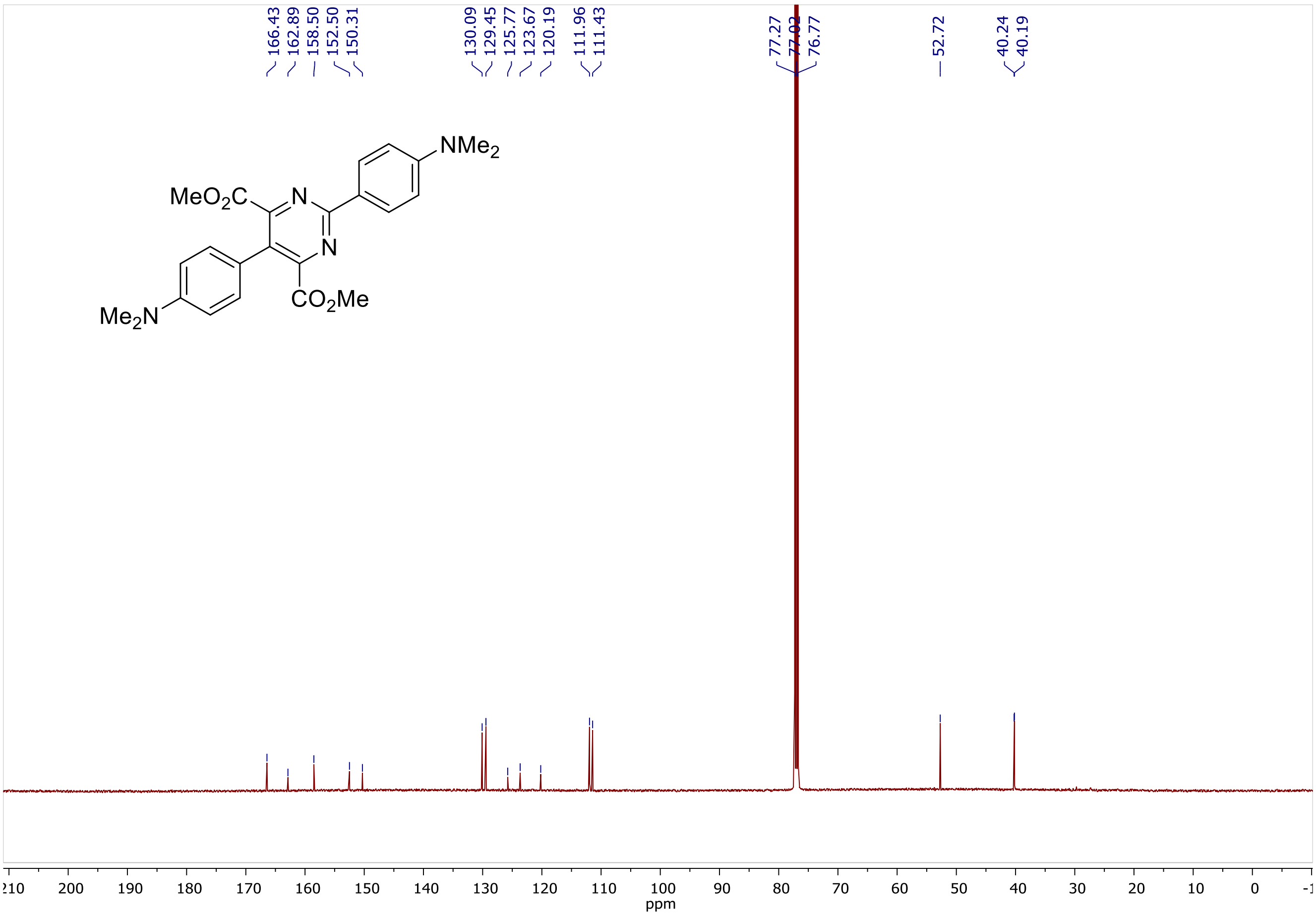

^{1}H NMR (400 MHz, $CDCl_3$) spectrum of dimethyl 2,5-di(naphthalen-2-yl)pyrimidine-4,6-dicarboxylate (**3g**)

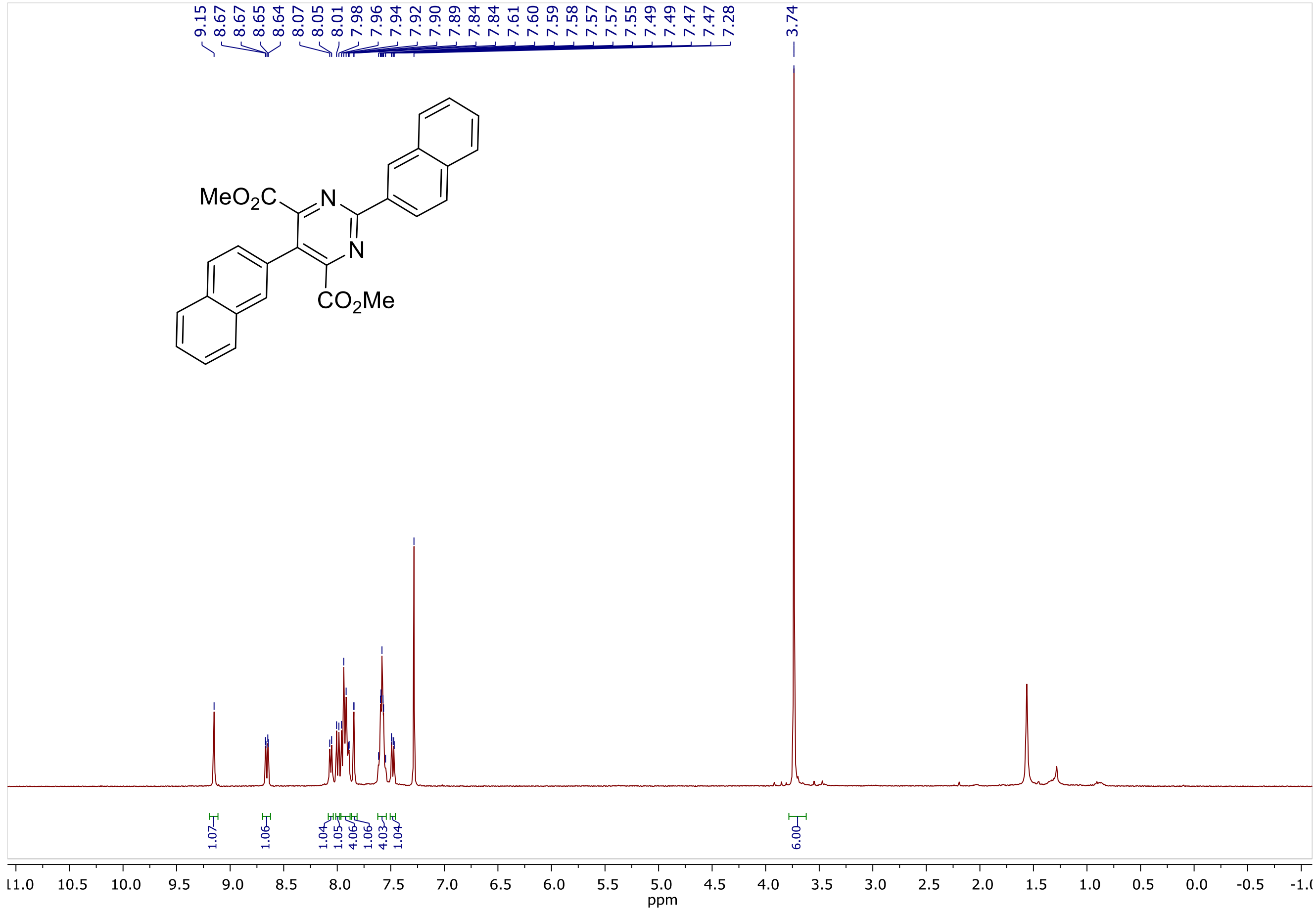

^{13}C{^{1}H} NMR (125 MHz, $CDCl_3$) spectrum of dimethyl 2,5-di(naphthalen-2-yl)pyrimidine-4,6-dicarboxylate (**3g**)

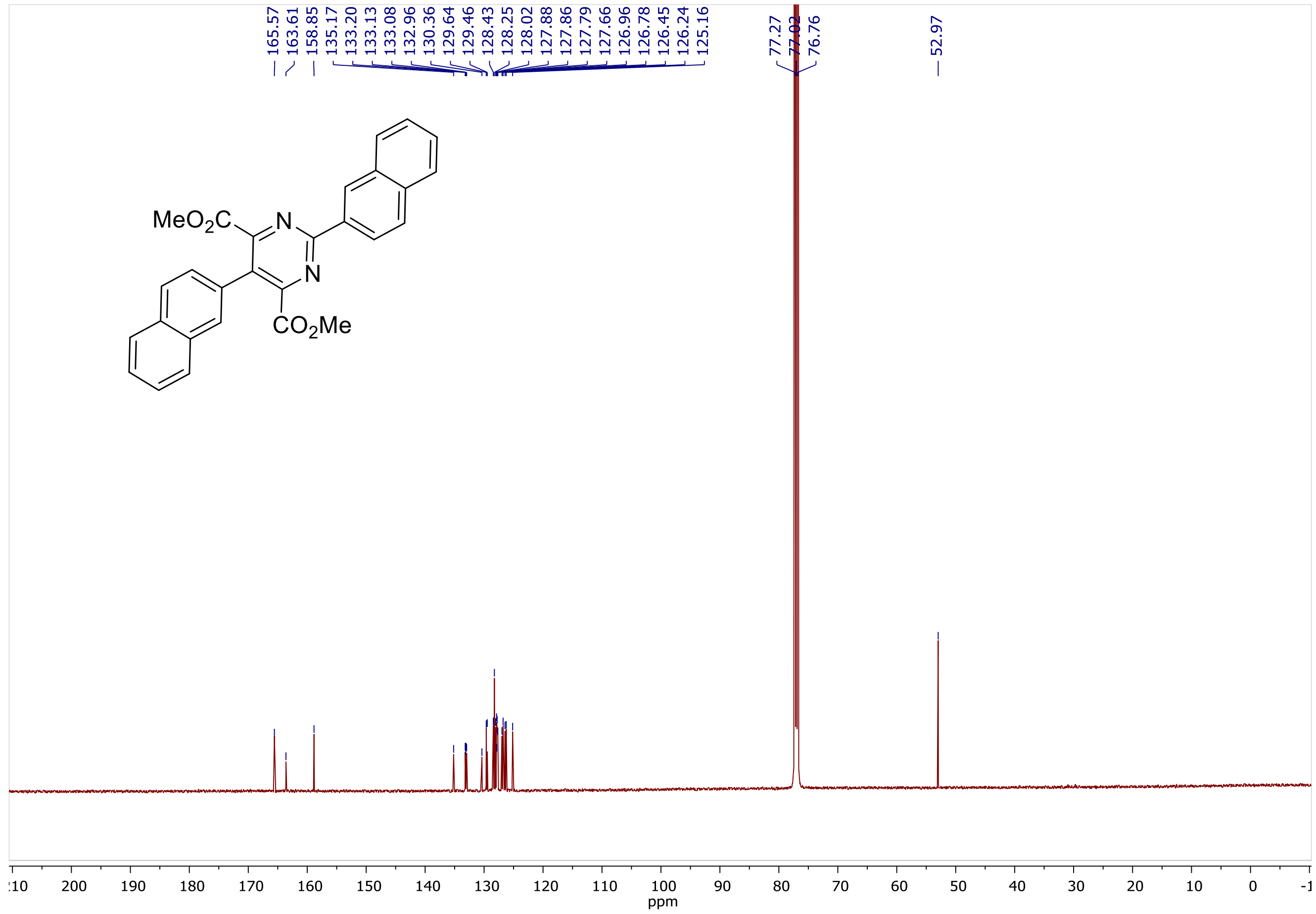

^{1}H NMR (400 MHz, $CDCl_3$) spectrum of dimethyl 2,5-di(4-biphenyl)pyrimidine-4,6-dicarboxylate (**3h**)

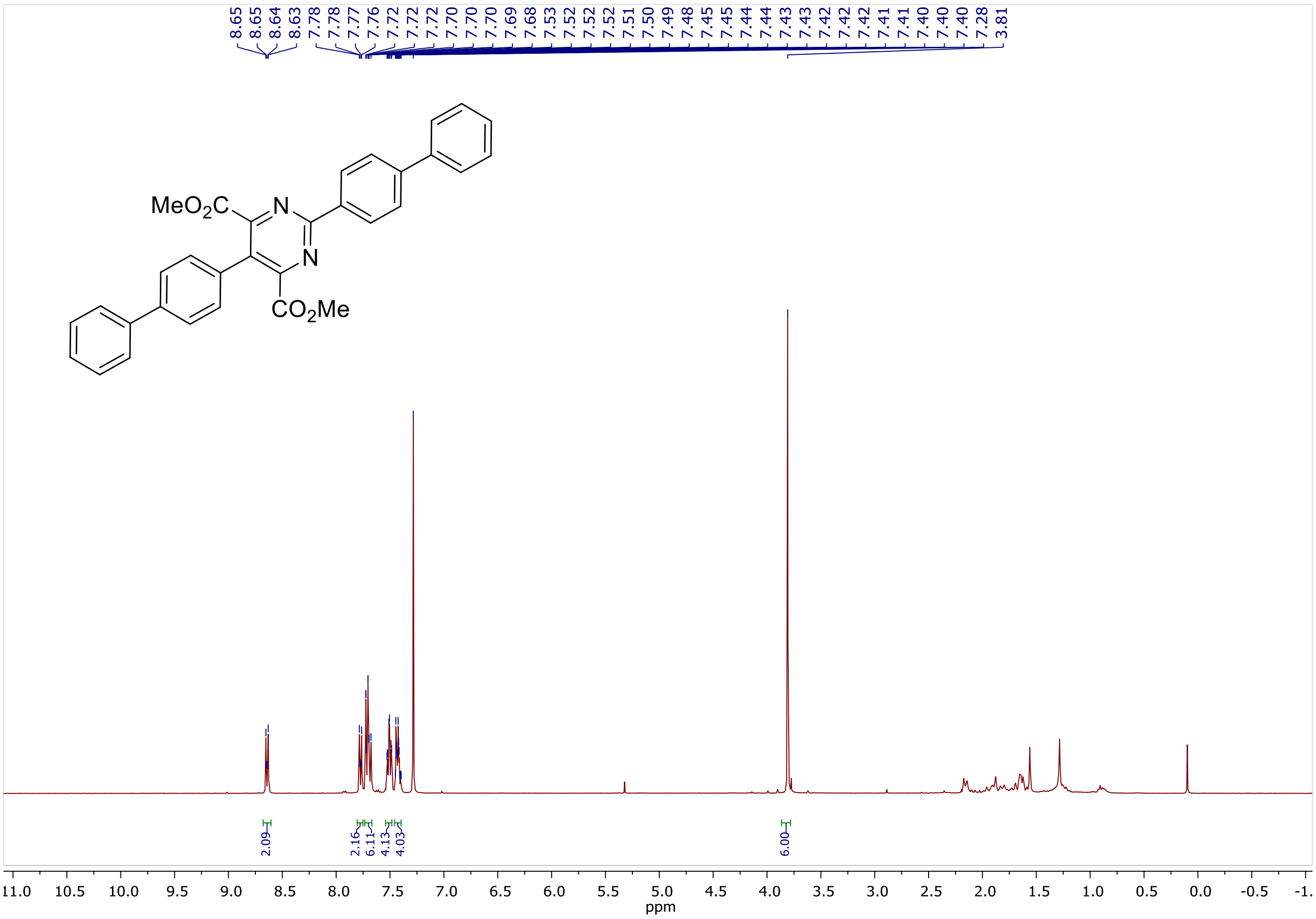

$^{13}C\{^{1}H\}$ NMR (125 MHz, $CDCl_3$) spectrum of dimethyl 2,5-di(4-biphenyl)pyrimidine-4,6-dicarboxylate (**3h**)

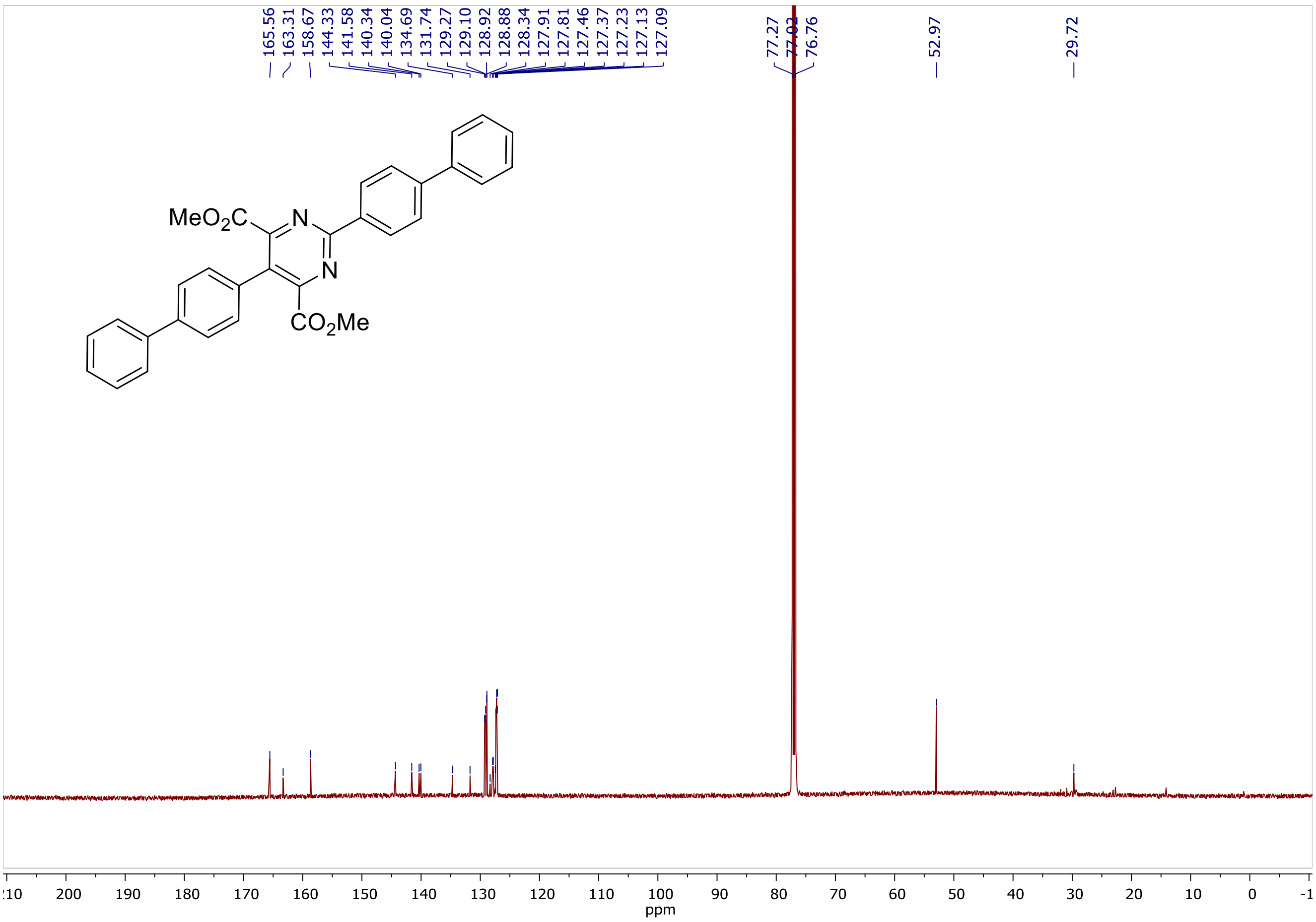

^{1}H NMR (400 MHz, $CDCl_3$) spectrum of di-*tert*-butyl 2,5-diphenylpyrimidine-4,6-dicarboxylate (**3i**)

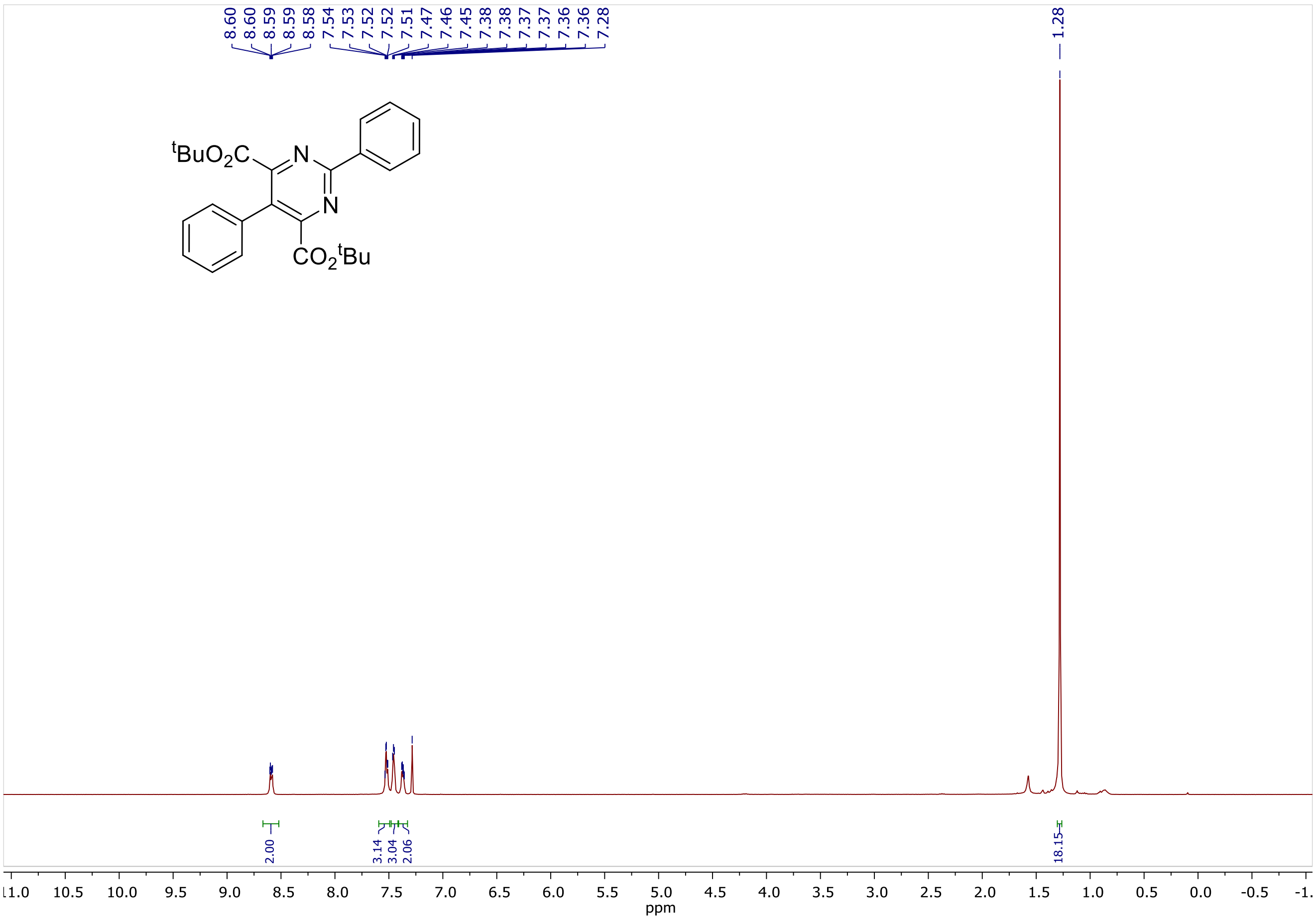

$^{13}C\{^{1}H\}$ NMR (100 MHz, $CDCl_3$) spectrum of di-*tert*-butyl 2,5-diphenylpyrimidine-4,6-dicarboxylate (**3i**)

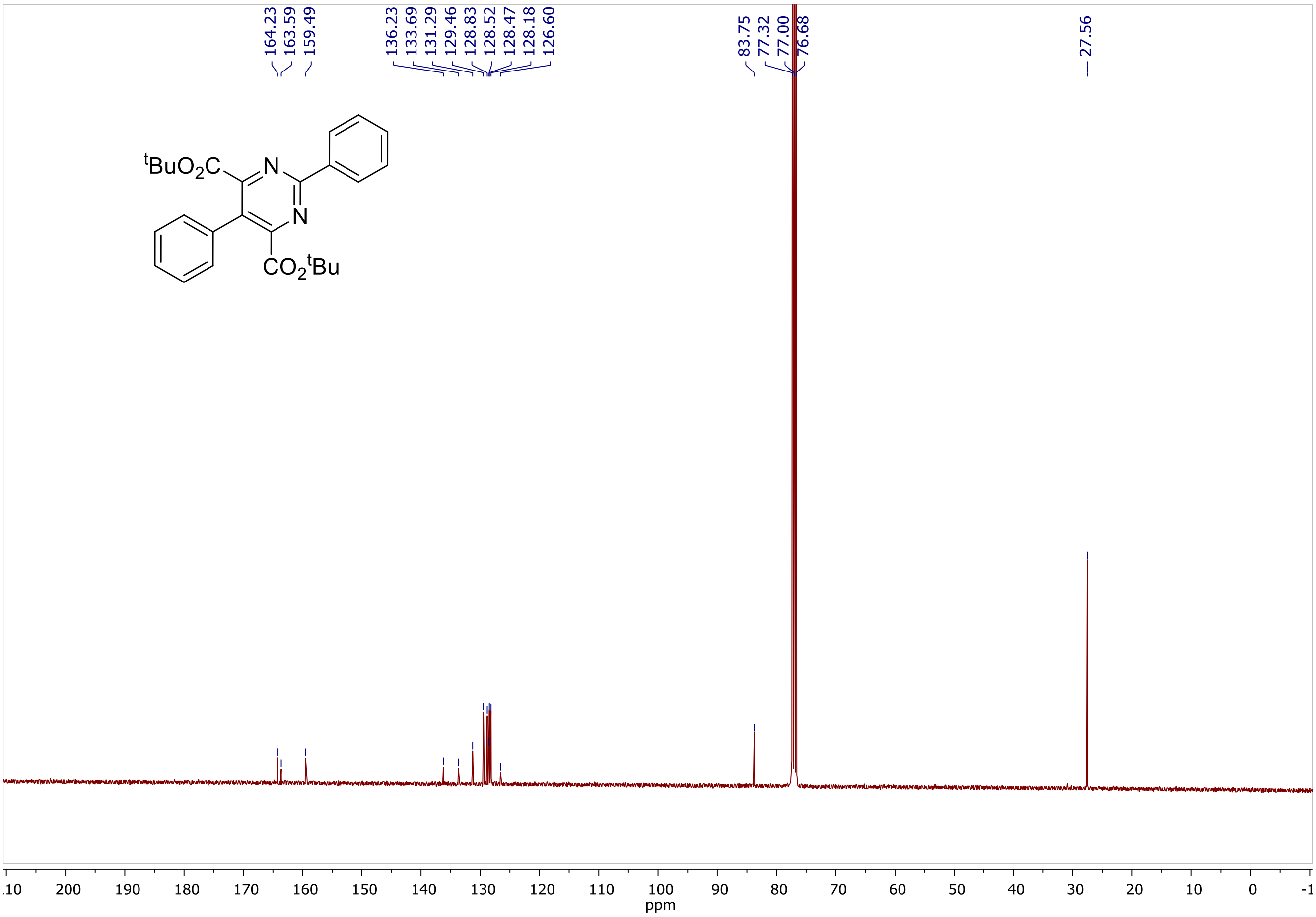

^{1}H NMR (400 MHz, $CDCl_3$) spectrum of methyl 2-benzamidoacetate (**4a**)

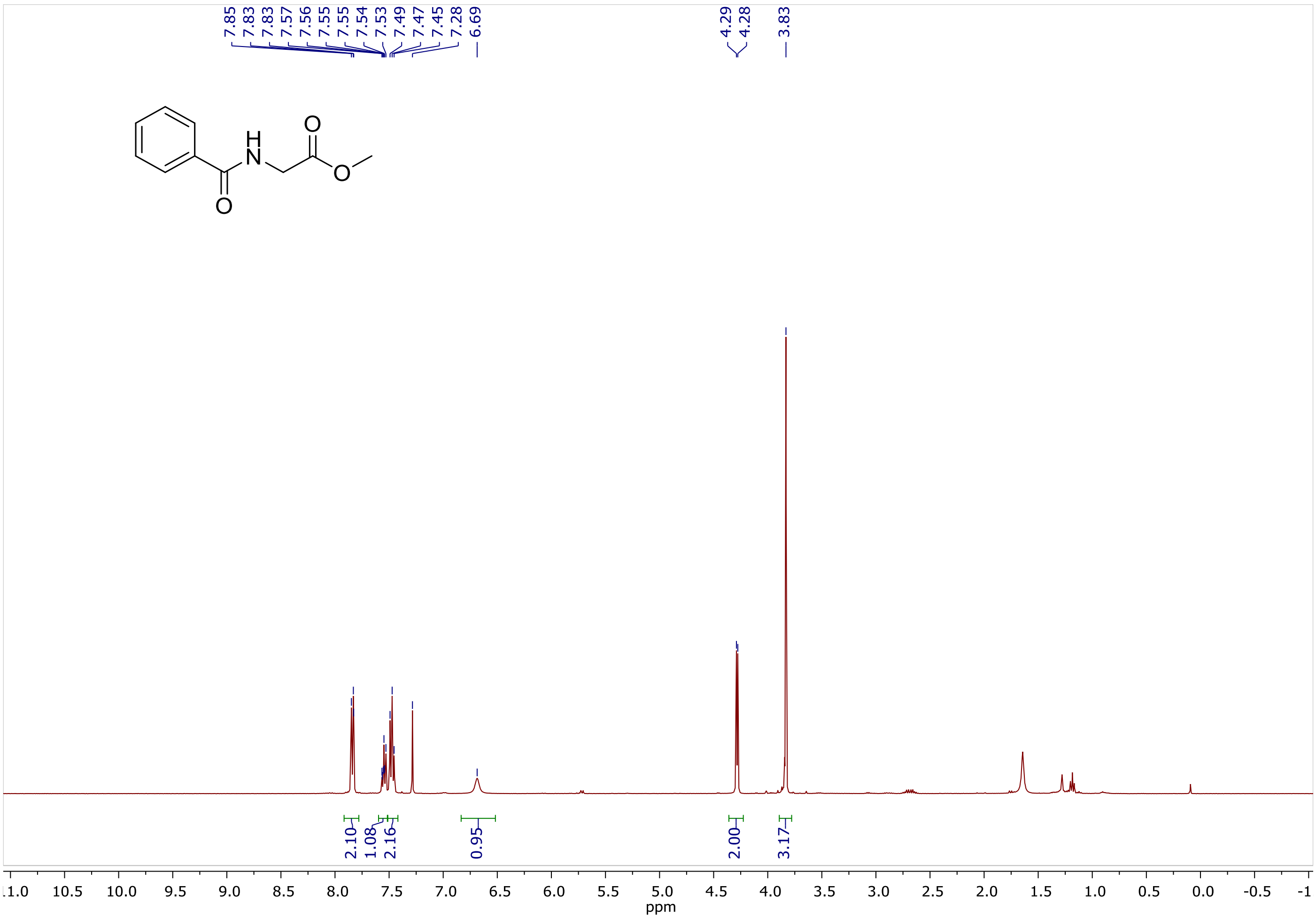

^{1}H NMR (400 MHz, $CDCl_3$) spectrum of methyl 2-benzamido-2-((diethylamino)oxy)acetate (**4b**)

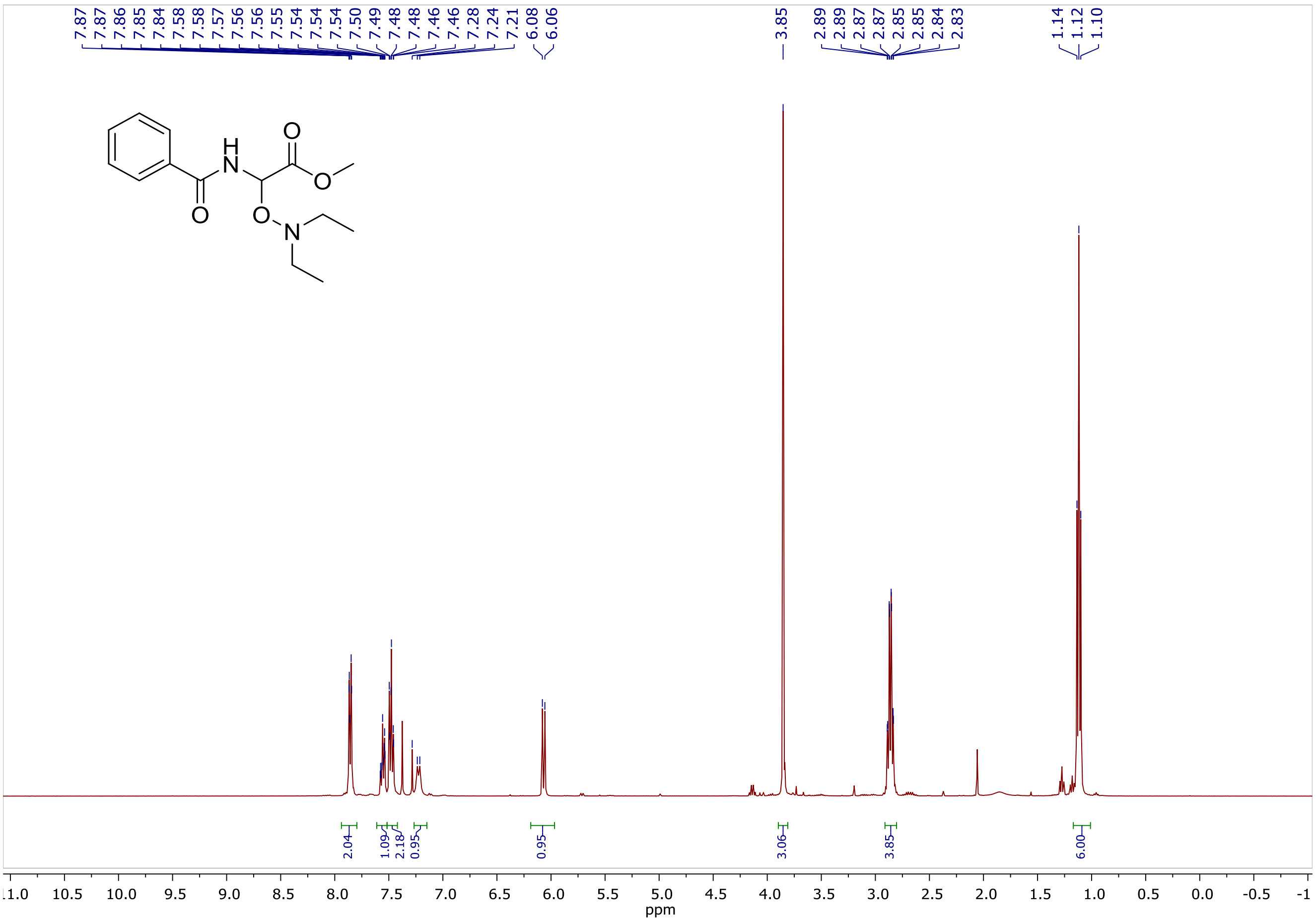

$^{13}C\{^{1}H\}$ NMR (100 MHz, $CDCl_3$) spectrum of methyl 2-benzamido-2-((diethylamino)oxy)acetate (**4b**)

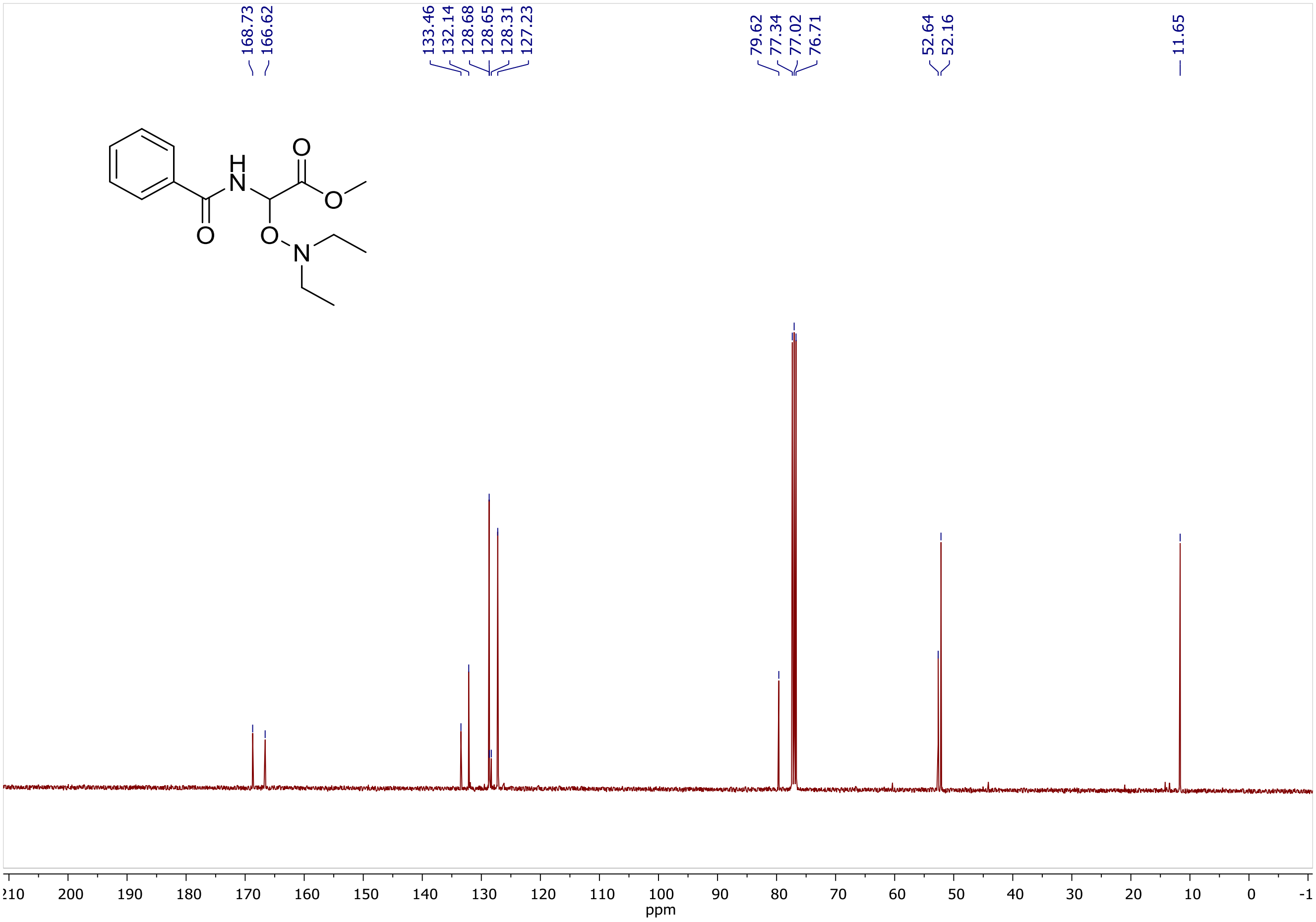

^{1}H–^{13}C HSQC spectrum (400 MHz, $CDCl_3$) of methyl 2-benzamido-2-((diethylamino)oxy)acetate (**4b**)

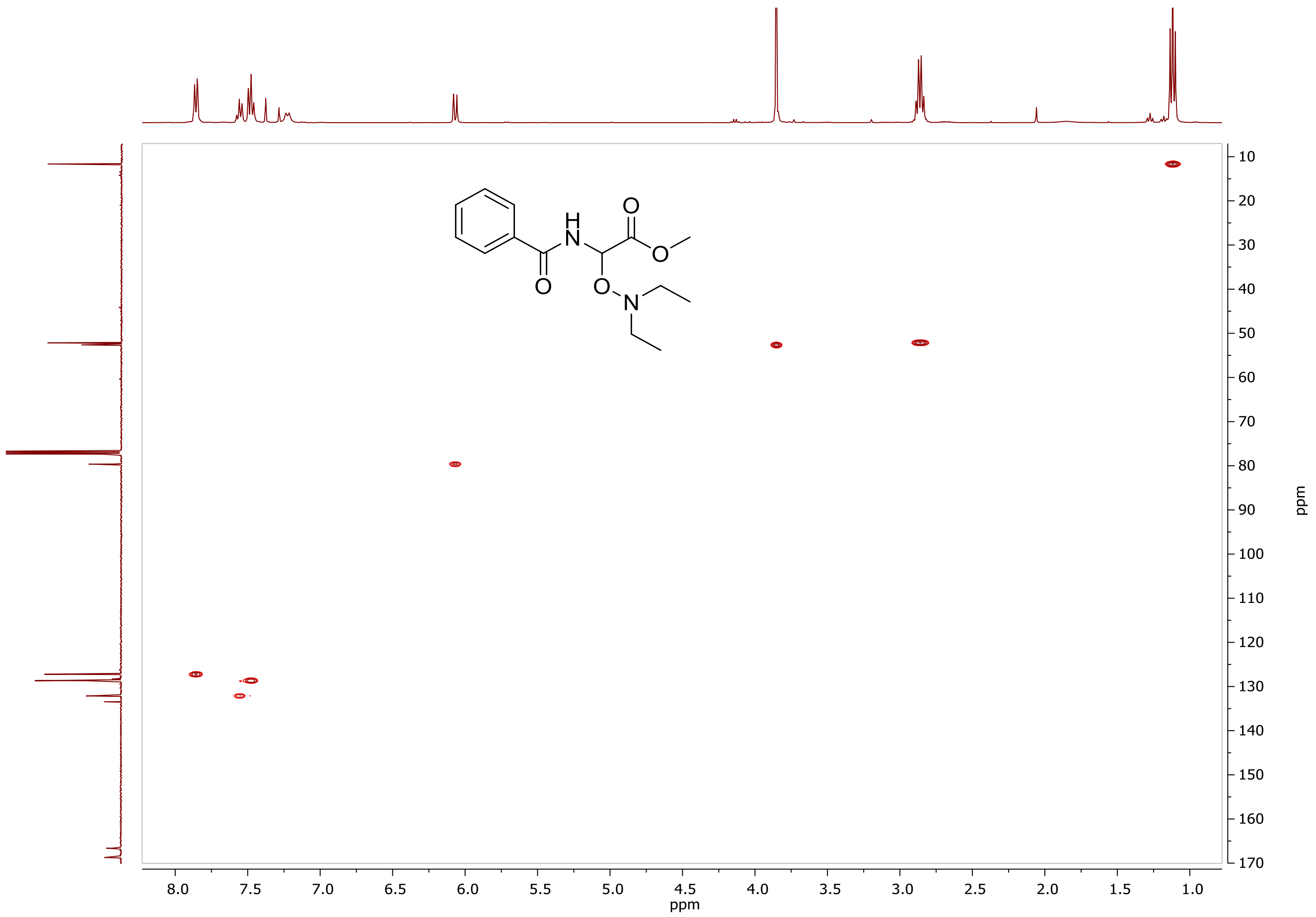

^{1}H–^{13}C HMBC spectrum (400 MHz, $CDCl_3$) of methyl 2-benzamido-2-((diethylamino)oxy)acetate (**4b**)

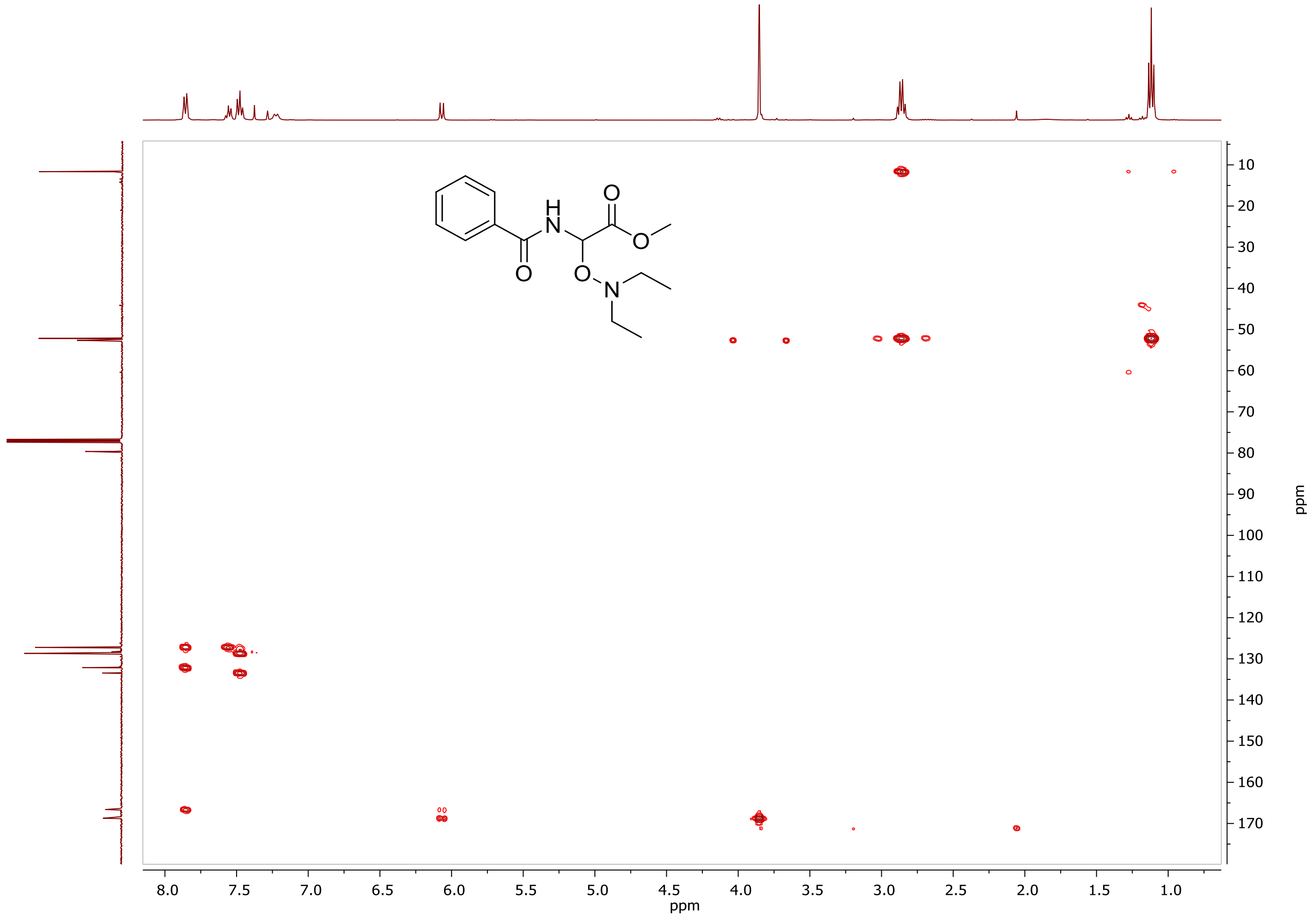

^{1}H NMR (400 MHz, CDCl$_3$) spectrum of methyl 2-benzamido-2-(diethylamino)acetate (**4c**)

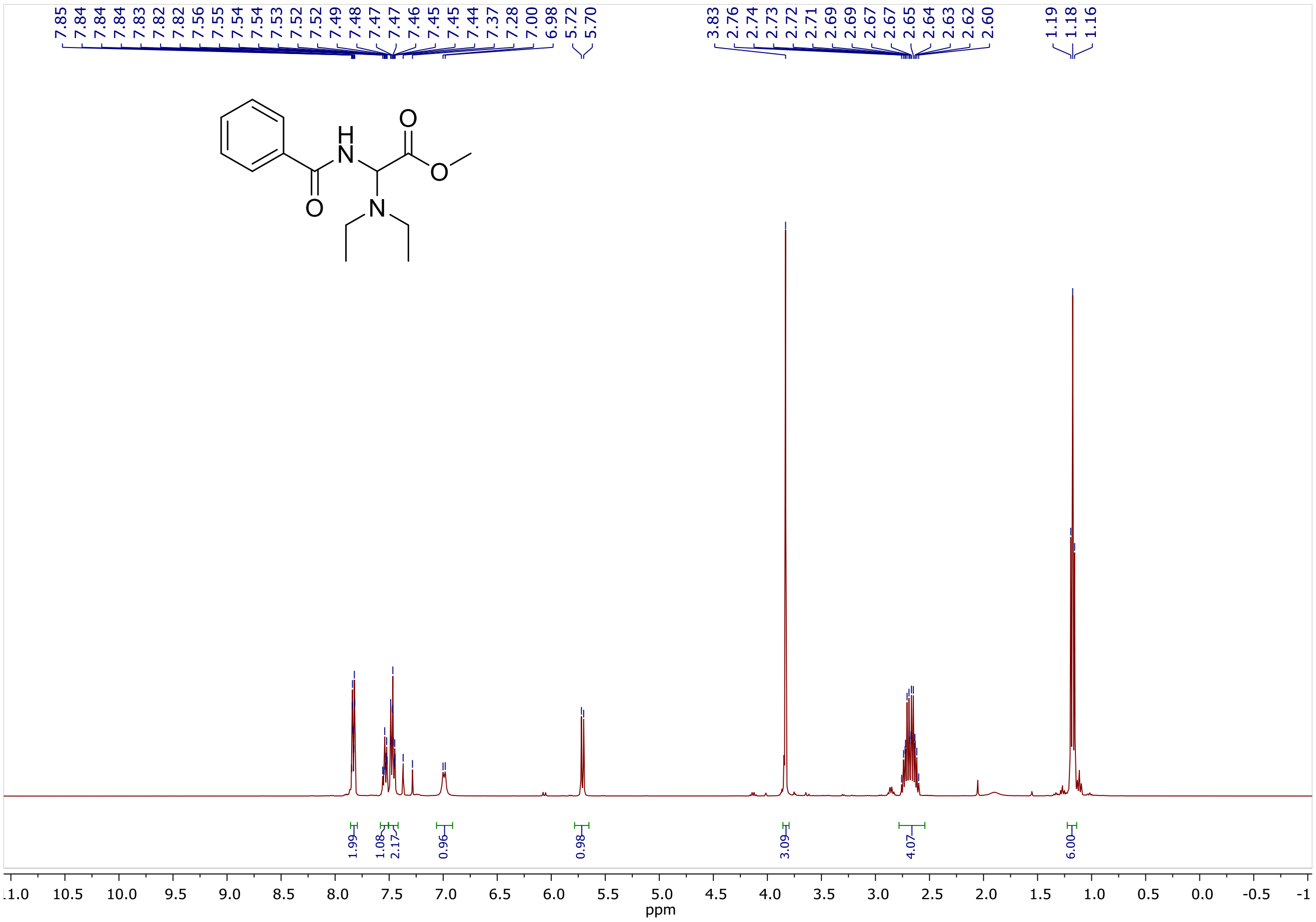

^{13}C{^{1}H} NMR (125 MHz, CDCl$_3$) spectrum of methyl 2-benzamido-2-(diethylamino)acetate (**4c**)

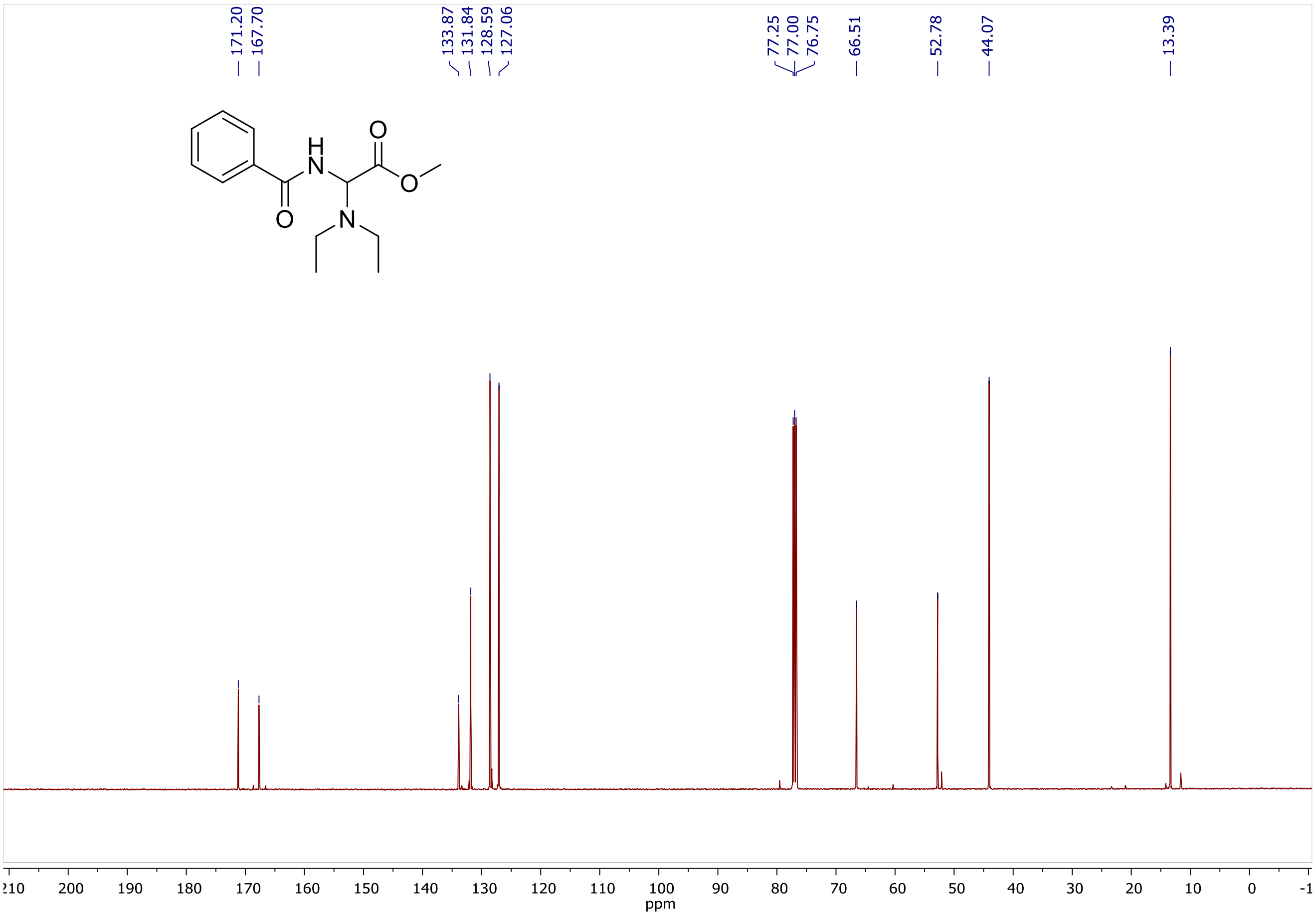

^{1}H–^{13}C HSQC spectrum (500 MHz, $CDCl_3$) of methyl 2-benzamido-2-(diethylamino)acetate (**4c**)

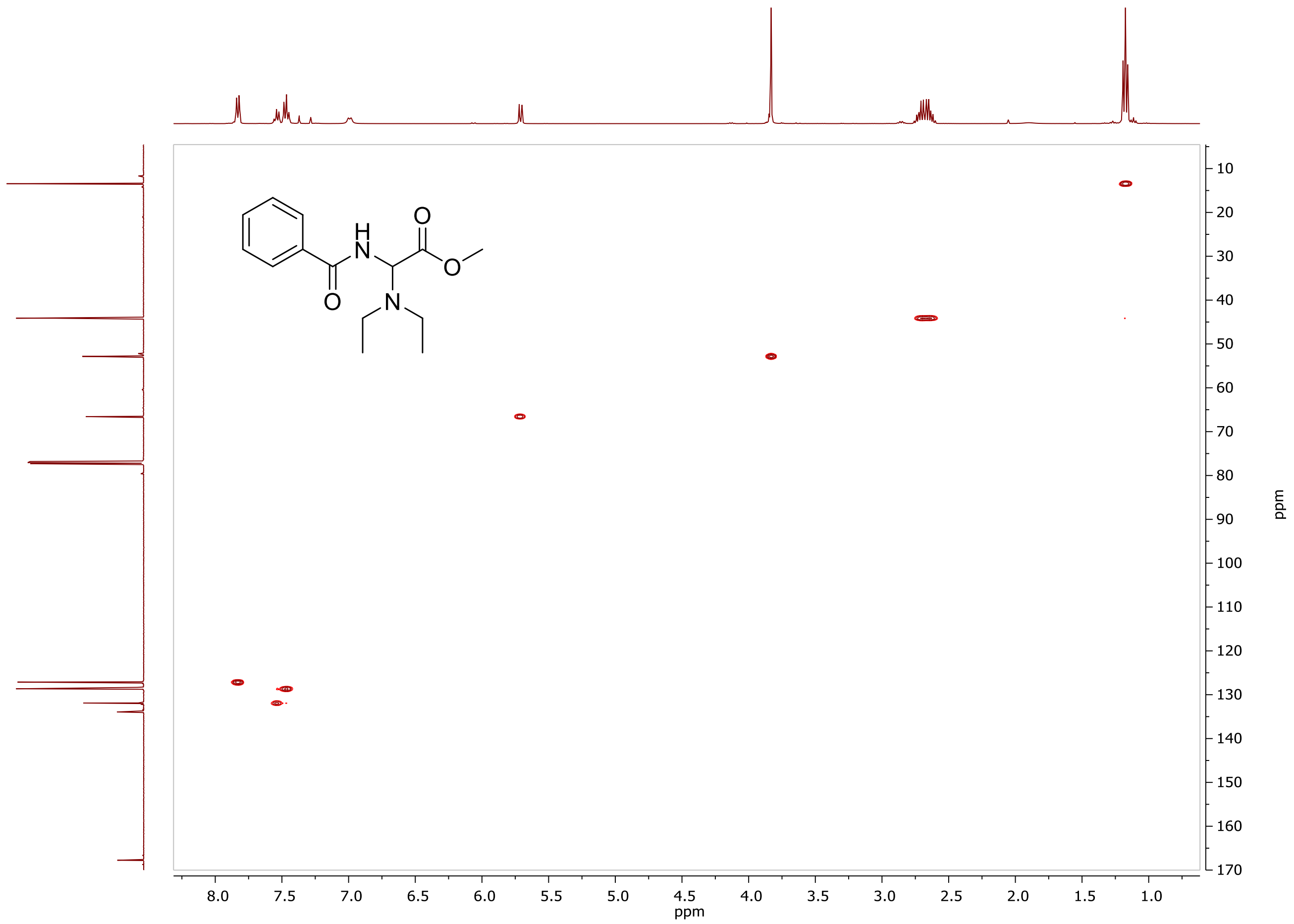

^{1}H–^{13}C HMBC spectrum (500 MHz, $CDCl_3$) of methyl 2-benzamido-2-(diethylamino)acetate (**4c**)

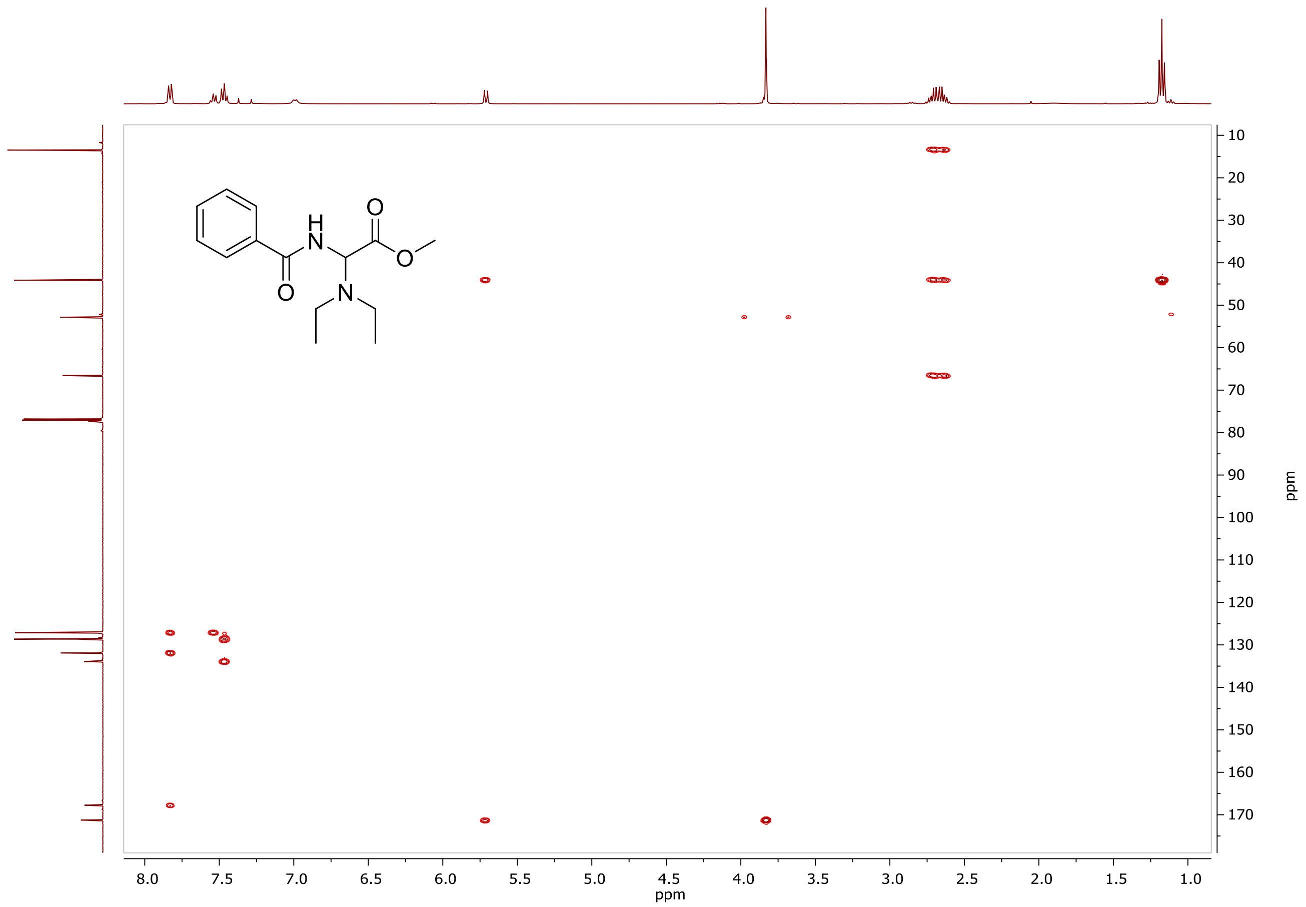

^{1}H NMR (500 MHz, $CDCl_3$, –40 °C) spectrum of crude methyl 3-(diethylaminooxy)-3-phenylaziridine-2-carboxylate (**8**)

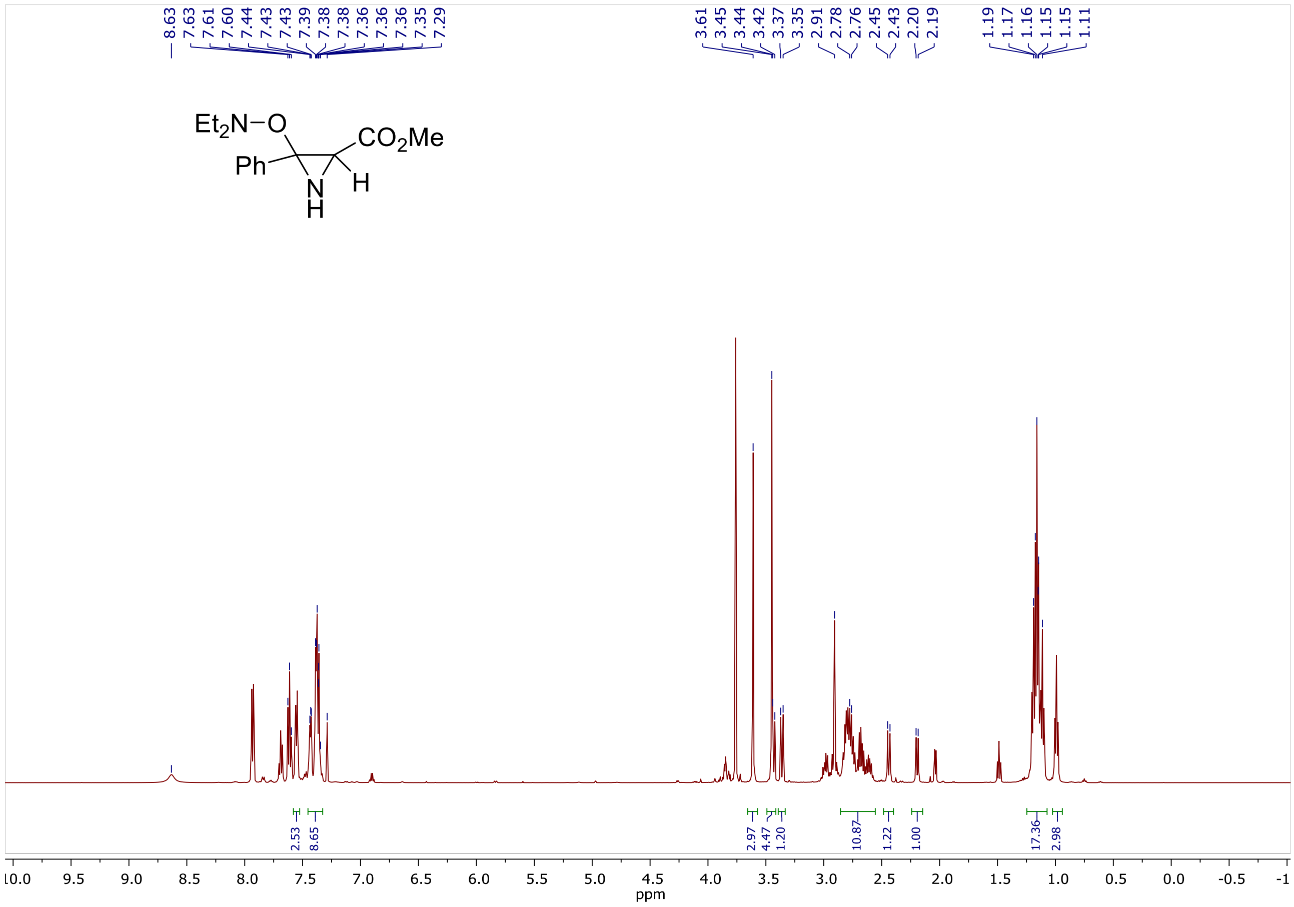

^{13}C{^{1}H} NMR (125 MHz, $CDCl_3$, –40 °C) spectrum of crude methyl 3-(diethylaminooxy)-3-phenylaziridine-2-carboxylate (**8**)

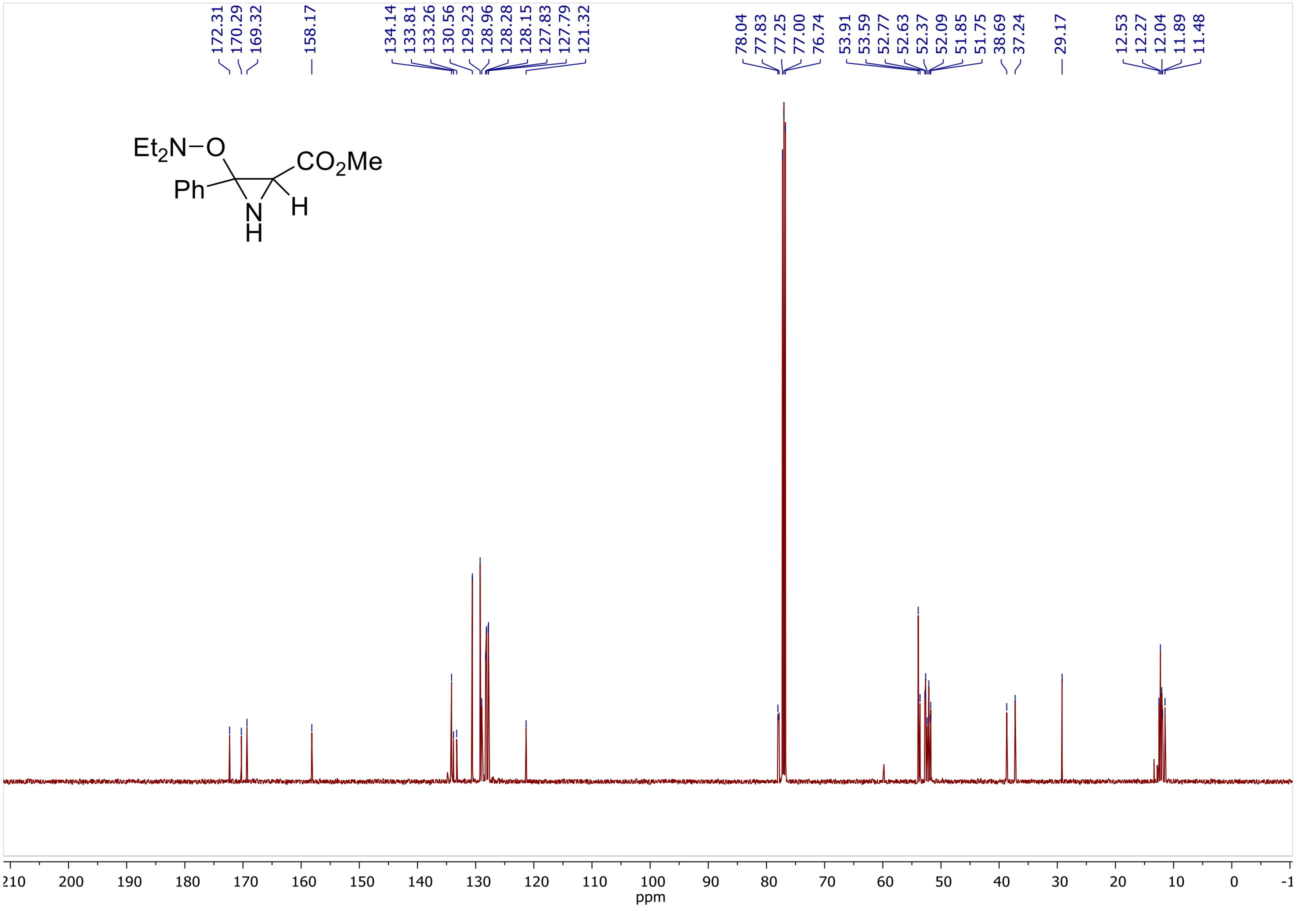

^{1}H–^{13}C HSQC spectrum (500 MHz, $CDCl_3$, –40 °C) of crude methyl 3-(diethylaminooxy)-3-phenylaziridine-2-carboxylate (**8**)

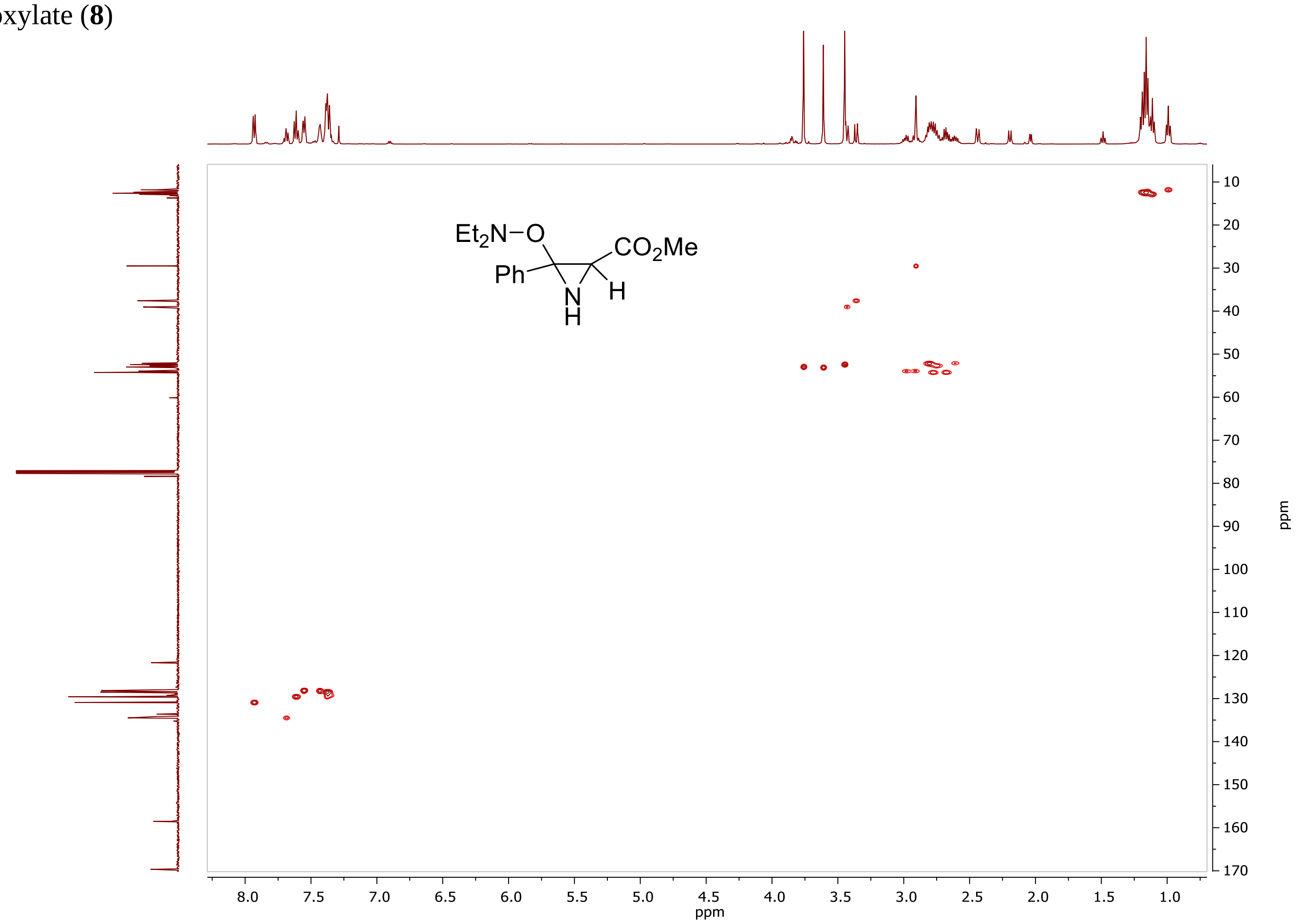

^{1}H–^{13}C HMBC spectrum (500 MHz, $CDCl_3$, –40 °C) of crude methyl 3-(diethylaminooxy)-3-phenylaziridine-2-carboxylate (**8**)

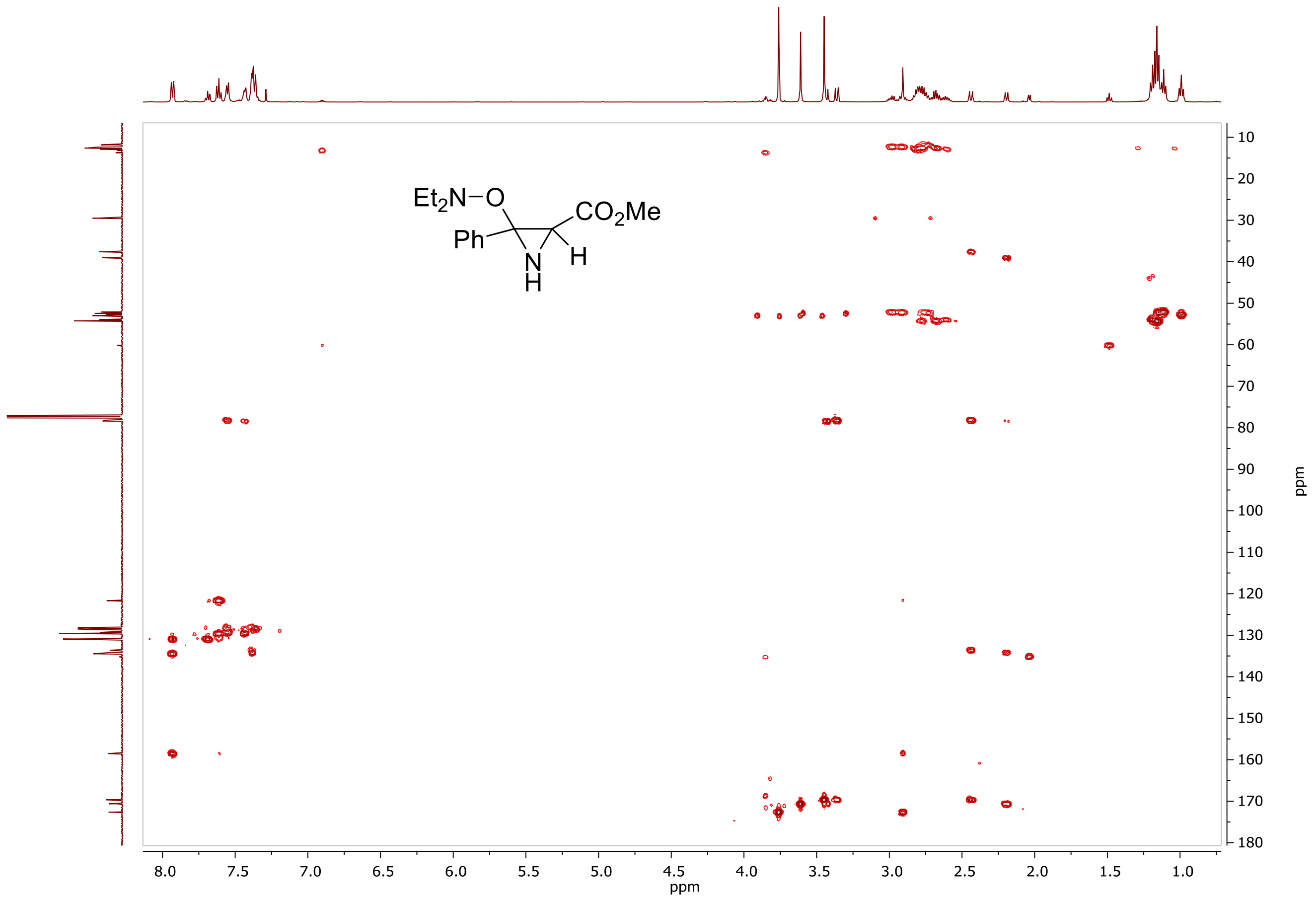

II. EPR spectroscopy

The reaction mixture of azirine **1a** (60 mg, 0.34 mmol) and triethylamine (90 mg, 0.88 mmol) in acetonitrile (1.5 mL) was stirred at 70 °C for 12 h. After cooling, a few drops of water were added to this reaction mixture to increase the signal resolution. The mixture was transferred into an EPR vial. The EPR experiment was carried out on a Bruker Elexsys E580 with a modulation amplitude of 1 G and at 323 K.

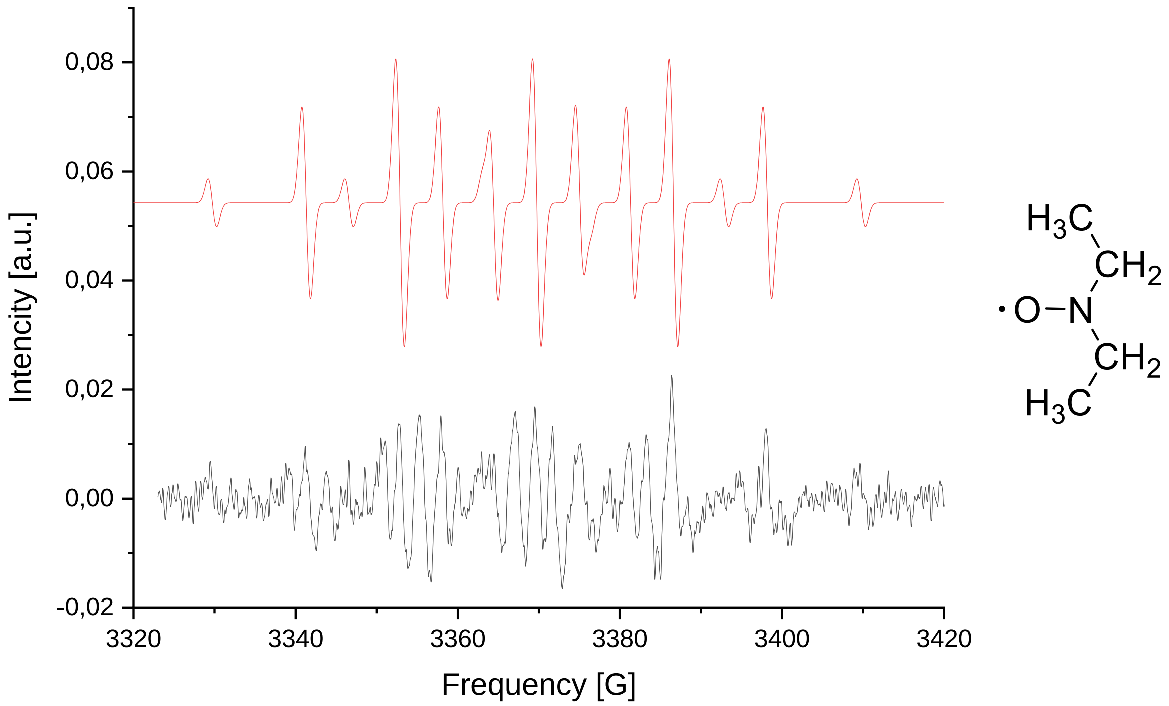

g-factor	2.00558
**a_N**	11.58 G
**a_H**	16.87 G

III. Calculations details

All calculations were performed by using the Gaussian 09 D.01 suit of quantum chemical programs.[1] The calculations were performed at the DFT rwb97xd/6-311+g(d,p) (Table S1) or ub3lyp/6-31g(d) (Table S2) level using PCM model for acetonitrile (343 K). The optimized structures were characterized by frequency analysis. Structures of the transition states were found by qst3 optimization model and optimized using Berny saddle point optimization algorithm.

Table S1. Energies (au) and cartesian coordinates of stationary points for compounds **1a**, **7**, **7'**, ***anti*-8**, ***syn*-8**, ***E*-9**, ***Z*-9**, ***anti*-10**, ***syn*-10**, **11** and transition states TS1, TS1a, TS1b, TS2a, TS2b, TS3a, TS3b, TS4a, TS4b (calculations in rwb97xd/6-311+g(d,p)).

azirine **1a**

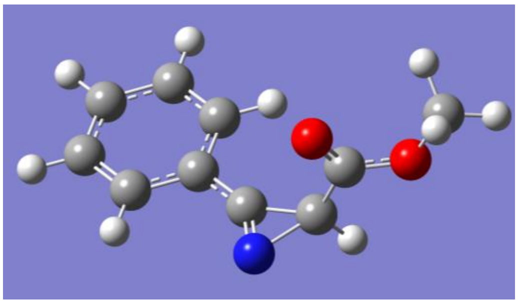

Zero-point correction = 0.171506 (Hartree/Particle)
Thermal correction to Energy = 0.186245
Thermal correction to Enthalpy = 0.187331
Thermal correction to Gibbs Free Energy = 0.124140
Sum of electronic and zero-point Energies = -591.422847
Sum of electronic and thermal Energies = -591.408107
Sum of electronic and thermal Enthalpies = -591.407021
Sum of electronic and thermal Free Energies = -591.470212
Imaginary frequencies = 0

C	-3.835753000	0.908175000	0.211051000
C	-3.682270000	-0.452503000	0.472147000
C	-2.453138000	-1.061626000	0.274946000
C	-1.374205000	-0.300975000	-0.186864000
C	-1.527313000	1.061186000	-0.448183000
C	-2.761655000	1.664330000	-0.247669000
C	-0.087146000	-0.921268000	-0.397132000
N	0.546686000	-1.995658000	-0.314714000
C	1.299771000	-0.762125000	-0.814846000
C	2.275140000	-0.159528000	0.134987000
O	3.265817000	0.444255000	-0.518332000
O	2.183086000	-0.203411000	1.339511000
C	4.261781000	1.094546000	0.282768000
H	-4.799023000	1.380199000	0.366695000

azirinopyrroline **11**

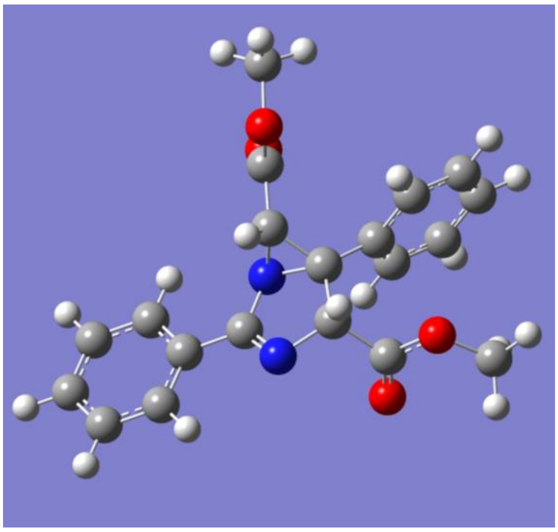

Zero-point correction = 0.349063 (Hartree/Particle)
Thermal correction to Energy = 0.378390
Thermal correction to Enthalpy = 0.379476
Thermal correction to Gibbs Free Energy = 0.281616
Sum of electronic and zero-point Energies = -1182.930276
Sum of electronic and thermal Energies = -1182.900950
Sum of electronic and thermal Enthalpies = -1182.899863
Sum of electronic and thermal Free Energies = -1182.997724
Imaginary frequencies = 0

N	1.204429000	-1.448845000	-1.076269000
C	1.663859000	-0.565347000	-0.280230000
N	0.723261000	0.365023000	0.273018000
C	-0.536477000	0.019363000	-0.379212000
C	-0.226775000	-1.243652000	-1.221571000
C	0.214157000	1.241234000	-0.796062000

[1] Gaussian 09, Revision D.01, Frisch, M. J.; Trucks, G.W.; Schlegel, H. B.; Scuseria, G. E.; Robb, M. A.; Cheeseman, J. R.; Scalmani, G.; Barone, V.; Mennucci, B.; Petersson, G. A.; Nakatsuji, H.; Caricato, M.; Li, X.; Hratchian, H. P.; Izmaylov, A. F.; Bloino, J.; Zheng, G.; Sonnenberg, J. L.; Hada, M.; Ehara, M.; Toyota, K.; Fukuda, R.; Hasegawa, J.; Ishida, M.; Nakajima, T.; Honda, Y.; Kitao, O.; Nakai, H.; Vreven, T.; Montgomery, J. A.; Peralta, J. E.; Ogliaro, F.; Bearpark, M.; Heyd, J. J.; Brothers, E.; Kudin, K. N.; Staroverov, V. N.; Kobayashi, R.; Normand, J.; Raghavachari, K.; Rendell, A.; Burant, J. C.; Iyengar, S. S.; Tomasi, J.; Cossi, M.; Rega, N.; Millam, N. J.; Klene, M.; Knox, J. E.; Cross, J. B.; Bakken, V.; Adamo, C.; Jaramillo, J.; Gomperts, R.; Stratmann, R. E.; Yazyev, O.; Austin, A. J.; Cammi, R.; Pomelli, C.; Ochterski, J. W.; Martin, R. L.; Morokuma, K.; Zakrzewski, V. G.; Voth, G. A.; Salvador, P.; Dannenberg, J. J.; Dapprich, S.; Daniels, A. D.; Farkas, Ő.; Foresman, J. B.; Ortiz, J. V.; Cioslowski, J.; Fox, D. J. Gaussian 09, Revision C.01; Gaussian: Wallingford CT, 2013.

H	-4.523124000	-1.034805000	0.829543000
H	-2.320382000	-2.118785000	0.474153000
H	-0.680708000	1.636681000	-0.805473000
H	-2.886902000	2.721595000	-0.447798000
H	1.566212000	-0.735285000	-1.865857000
H	3.810853000	1.898008000	0.866255000
H	4.738891000	0.376872000	0.950523000
H	4.984882000	1.497109000	-0.421704000

C	3.079281000	-0.425743000	0.091982000
C	-1.008554000	-2.435279000	-0.681471000
O	-2.207960000	-2.499009000	-1.246161000
O	-0.613330000	-3.186012000	0.173173000
C	-3.125230000	-3.453238000	-0.691951000
C	-0.267548000	2.594649000	-0.372111000
O	-0.614995000	3.298322000	-1.445780000
O	-0.353766000	2.987346000	0.762492000
C	-1.161937000	4.603706000	-1.206736000
C	-1.816537000	0.110592000	0.383115000
C	3.483359000	0.597577000	0.950750000
C	4.826282000	0.738293000	1.280749000
C	5.767143000	-0.142996000	0.760527000
C	5.366089000	-1.168118000	-0.094176000
C	4.028579000	-1.309924000	-0.429402000
C	-2.946666000	0.639578000	-0.236321000
C	-4.161152000	0.669499000	0.437153000
C	-4.251209000	0.167964000	1.732112000
C	-3.122969000	-0.358458000	2.352706000
C	-1.906666000	-0.387039000	1.680076000
H	0.720153000	1.215514000	-1.756174000
H	-4.048783000	-3.330018000	-1.250966000
H	-2.737387000	-4.465026000	-0.810642000
H	-3.287842000	-3.238015000	0.365269000
H	-1.347428000	5.026689000	-2.190291000
H	-2.092887000	4.521053000	-0.644907000
H	-0.449568000	5.217631000	-0.655588000
H	2.744549000	1.278183000	1.356083000
H	5.136608000	1.534908000	1.946788000
H	6.814357000	-0.031894000	1.018143000
H	6.099467000	-1.856162000	-0.498460000
H	3.706953000	-2.099967000	-1.097232000
H	-2.874684000	1.024889000	-1.248310000
H	-5.037303000	1.084421000	-0.047766000
H	-5.199162000	0.190604000	2.257611000
H	-3.189371000	-0.746982000	3.362545000
H	-1.022842000	-0.792027000	2.159965000
H	-0.490426000	-1.108181000	-2.272677000

diethylamine oxide **7'**

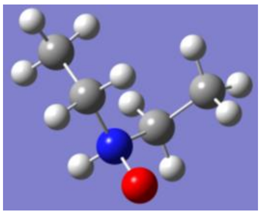

Zero-point correction = 0.156980 (Hartree/Particle)
Thermal correction to Energy = 0.166107
Thermal correction to Enthalpy = 0.167193
Thermal correction to Gibbs Free Energy = 0.120367
Sum of electronic and zero-point Energies = -288.801494
Sum of electronic and thermal Energies = -288.792367
Sum of electronic and thermal Enthalpies = -288.791281
Sum of electronic and thermal Free Energies = -288.838107
Imaginary frequencies = 0

N	0.084890000	0.691608000	0.358546000

N,*N*-diethylhydroxylamine **7**

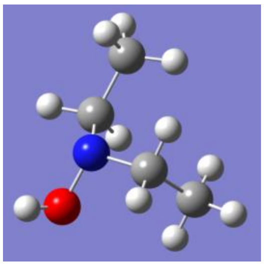

Zero-point correction = 0.154088 (Hartree/Particle)
Thermal correction to Energy = 0.163974
Thermal correction to Enthalpy = 0.165060
Thermal correction to Gibbs Free Energy = 0.116705
Sum of electronic and zero-point Energies = -288.814055
Sum of electronic and thermal Energies = -288.804170
Sum of electronic and thermal Enthalpies = -288.803083
Sum of electronic and thermal Free Energies = -288.851438
Imaginary frequencies = 0

C	0.914390000	-0.508200000	0.715643000
H	1.726004000	-0.106364000	1.321410000
H	0.305137000	-1.159166000	1.346907000
C	-1.106025000	0.370921000	-0.490082000
H	-1.615409000	1.325869000	-0.615879000
H	-0.712105000	0.070789000	-1.460344000
C	-2.030500000	-0.675383000	0.107286000
H	-2.936291000	-0.729566000	-0.499072000
H	-1.577441000	-1.668558000	0.119984000
H	-2.328998000	-0.414454000	1.126450000
C	1.448122000	-1.241939000	-0.497048000
H	2.163837000	-1.995534000	-0.163883000
H	0.657563000	-1.755630000	-1.048347000
H	1.961120000	-0.551942000	-1.167979000
O	0.836798000	1.679612000	-0.216694000
H	-0.287958000	1.034015000	1.249688000

C	1.083645000	0.425625000	-0.455585000
H	0.748324000	0.102561000	-1.451822000
H	1.563329000	1.399417000	-0.577141000
C	2.082408000	-0.570927000	0.114972000
H	1.685494000	-1.588925000	0.126552000
H	2.984886000	-0.574236000	-0.499877000
H	2.364273000	-0.299561000	1.135779000
C	-0.848152000	-0.565635000	0.715778000
H	-0.226783000	-1.212428000	1.341278000
H	-1.684788000	-0.241174000	1.338650000
C	-1.364220000	-1.336475000	-0.497527000
H	-0.551080000	-1.790499000	-1.069447000
H	-2.024410000	-2.140831000	-0.164064000
H	-1.934092000	-0.684159000	-1.162791000
N	-0.053521000	0.635159000	0.441676000
O	-0.912906000	1.570777000	-0.221540000
H	-0.969343000	2.301977000	0.397643000

aziridine ***syn*-8**

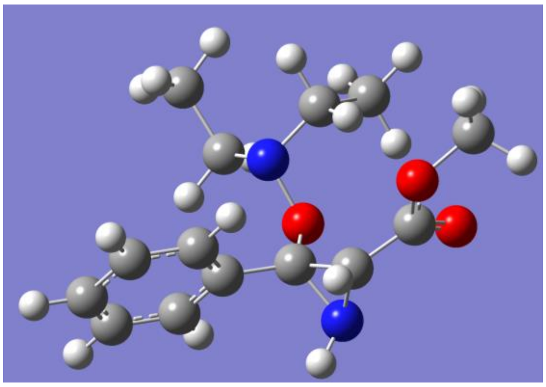

Zero-point correction = 0.330375 (Hartree/Particle)
Thermal correction to Energy = 0.355002
Thermal correction to Enthalpy = 0.356088
Thermal correction to Gibbs Free Energy = 0.270431
Sum of electronic and zero-point Energies = -880.262914
Sum of electronic and thermal Energies = -880.238287
Sum of electronic and thermal Enthalpies = -880.237201
Sum of electronic and thermal Free Energies = -880.322858
Imaginary frequencies = 0

C	0.837995000	-1.733995000	-0.394290000
C	-0.189783000	-0.690555000	-0.736986000
H	0.511486000	-2.552193000	0.238283000
C	-1.583076000	-0.807016000	-0.195078000
C	-2.653802000	-0.495146000	-1.032885000
C	-1.828675000	-1.200078000	1.116150000
C	-3.958332000	-0.583103000	-0.563361000
H	-2.462805000	-0.182306000	-2.054189000
C	-3.134608000	-1.288899000	1.586761000
H	-0.998249000	-1.426061000	1.775847000
C	-4.200504000	-0.980442000	0.748514000
H	-4.785510000	-0.341565000	-1.221198000
H	-3.318507000	-1.592369000	2.611106000
H	-5.217993000	-1.049661000	1.116528000
C	-0.742050000	2.595157000	-0.395586000
H	-0.244862000	3.075420000	-1.250205000
H	-1.683231000	2.178208000	-0.760495000

aziridine ***anti*-8**

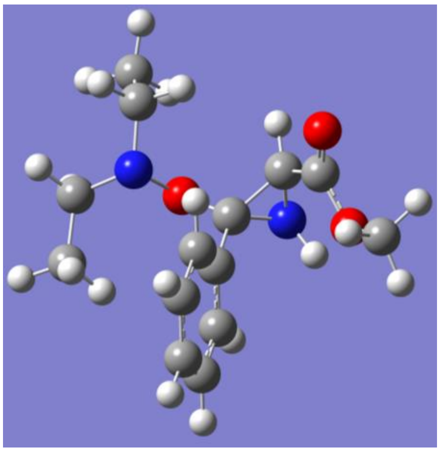

Zero-point correction = 0.330177 (Hartree/Particle)
Thermal correction to Energy = 0.354845
Thermal correction to Enthalpy = 0.355931
Thermal correction to Gibbs Free Energy = 0.270731
Sum of electronic and zero-point Energies = -880.265085
Sum of electronic and thermal Energies = -880.240417
Sum of electronic and thermal Enthalpies = -880.239330
Sum of electronic and thermal Free Energies = -880.324531
Imaginary frequencies = 0

C	-0.394883000	-1.796793000	-0.563255000
C	0.193253000	-0.427437000	-0.771874000
H	0.289053000	-2.622351000	-0.410879000
C	-0.494169000	0.811566000	-0.285042000
C	-0.835718000	1.806709000	-1.196682000
C	-0.751551000	0.999978000	1.069552000
C	-1.432294000	2.982664000	-0.757914000
H	-0.631372000	1.664962000	-2.253066000
C	-1.350058000	2.174507000	1.509817000
H	-0.475210000	0.228715000	1.779077000
C	-1.688607000	3.168397000	0.596880000
H	-1.697356000	3.752920000	-1.473157000
H	-1.548356000	2.316091000	2.566169000
H	-2.154339000	4.085121000	0.940815000
C	3.170921000	0.949495000	0.227116000

C	-1.021214000	3.614983000	0.697701000
H	-1.742085000	4.348721000	0.331245000
H	-1.444534000	3.131950000	1.582194000
H	-0.120615000	4.156718000	0.995912000
C	1.361444000	1.799159000	0.633042000
H	1.181635000	2.387065000	1.536735000
H	1.808287000	0.863522000	0.974353000
C	2.312775000	2.532706000	-0.306573000
H	1.939834000	3.526101000	-0.569039000
H	3.281313000	2.659993000	0.183289000
H	2.467642000	1.962096000	-1.223999000
C	2.278536000	-1.374411000	-0.238730000
C	4.009869000	-1.226357000	1.349464000
H	4.126932000	-1.449847000	2.406574000
H	4.703515000	-1.820878000	0.754339000
H	4.182996000	-0.165017000	1.164279000
N	0.272352000	-1.607734000	-1.737538000
H	-0.410287000	-2.336492000	-1.915005000
N	0.038562000	1.462350000	0.102253000
O	3.008250000	-0.952208000	-1.098778000
O	0.225192000	0.607589000	-1.034179000
O	2.656208000	-1.571349000	1.028155000

H	3.745532000	0.990606000	1.156845000
H	3.865980000	0.665354000	-0.575673000
C	2.560166000	2.309772000	-0.067164000
H	3.348132000	3.064619000	-0.127952000
H	2.023218000	2.299761000	-1.017214000
H	1.856211000	2.597615000	0.717397000
C	2.693351000	-1.338726000	0.965340000
H	3.100094000	-1.091246000	1.949991000
H	1.841545000	-1.999078000	1.143589000
C	3.741790000	-2.052392000	0.115935000
H	4.661309000	-1.469563000	0.025395000
H	3.996696000	-3.007752000	0.580772000
H	3.357004000	-2.252354000	-0.886182000
C	-1.684000000	-1.949726000	0.167337000
C	-3.944187000	-1.294522000	0.261003000
H	-4.612577000	-0.714879000	-0.369797000
H	-4.325973000	-2.306747000	0.394801000
H	-3.820989000	-0.811960000	1.231222000
N	-0.323955000	-1.165147000	-1.886728000
H	-1.238309000	-0.812448000	-2.155413000
N	2.157807000	-0.081231000	0.434932000
O	-1.786662000	-2.569053000	1.200398000
O	1.586355000	-0.348270000	-0.852513000
O	-2.689861000	-1.321889000	-0.432943000

azomethine-ylide ***Z*-9**

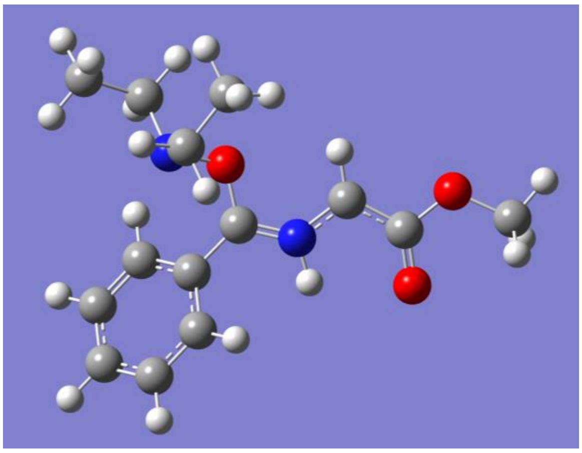

Zero-point correction = 0.329622 (Hartree/Particle)
Thermal correction to Energy = 0.354836
Thermal correction to Enthalpy = 0.355923
Thermal correction to Gibbs Free Energy = 0.269126
Sum of electronic and zero-point Energies = -880.257721
Sum of electronic and thermal Energies = -880.232506
Sum of electronic and thermal Enthalpies = -880.231420
Sum of electronic and thermal Free Energies = -880.318217
Imaginary frequencies = 0

C	2.226036000	0.320690000	-0.280002000
C	-0.126341000	-0.279022000	-0.262532000
H	2.060240000	1.367340000	-0.460781000
C	-1.137467000	-1.325829000	-0.087217000
C	-2.271867000	-1.350677000	-0.904945000
C	-0.964389000	-2.317069000	0.881416000
C	-3.206943000	-2.363220000	-0.762974000
H	-2.412367000	-0.583131000	-1.655779000

azomethine-ylide ***E*-9**

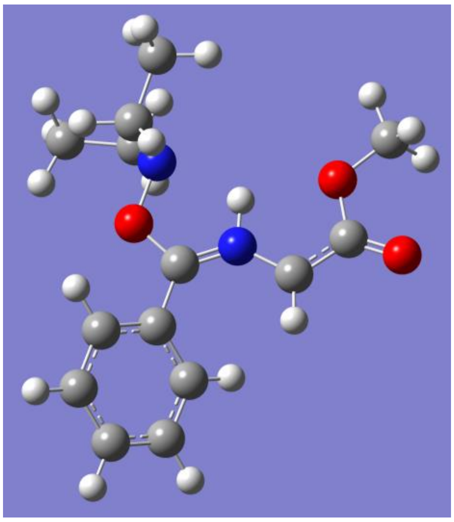

Zero-point correction = 0.329472 (Hartree/Particle)
Thermal correction to Energy = 0.354824
Thermal correction to Enthalpy = 0.355910
Thermal correction to Gibbs Free Energy = 0.267916
Sum of electronic and zero-point Energies = -880.257783
Sum of electronic and thermal Energies = -880.232430
Sum of electronic and thermal Enthalpies = -880.231344
Sum of electronic and thermal Free Energies = -880.319338
Imaginary frequencies = 0

C	2.226036000	0.320690000	-0.280002000
C	-0.126341000	-0.279022000	-0.262532000
H	2.060240000	1.367340000	-0.460781000
C	-1.137467000	-1.325829000	-0.087217000
C	-2.271867000	-1.350677000	-0.904945000
C	-0.964389000	-2.317069000	0.881416000

C	-1.904163000	-3.332158000	1.016468000
H	-0.111667000	-2.284417000	1.551199000
C	-3.025725000	-3.357381000	0.196607000
H	-4.080295000	-2.380124000	-1.404569000
H	-1.764381000	-4.094633000	1.773710000
H	-3.762134000	-4.145014000	0.306776000
C	-2.207389000	2.397171000	-0.925942000
H	-1.493217000	3.184884000	-1.199242000
H	-2.415044000	1.817512000	-1.827885000
C	-3.496992000	3.002560000	-0.393469000
H	-3.985553000	3.562756000	-1.192758000
H	-4.182148000	2.222859000	-0.052213000
H	-3.315371000	3.693485000	0.432775000
C	-1.215383000	2.055959000	1.321337000
H	-2.144645000	2.292552000	1.845238000
H	-0.742972000	1.250096000	1.890386000
C	-0.300329000	3.273826000	1.260968000
H	-0.792345000	4.133559000	0.800164000
H	-0.015739000	3.558127000	2.276353000
H	0.612480000	3.054337000	0.703403000
C	3.476592000	-0.304390000	-0.164219000
C	5.822989000	-0.005456000	-0.162255000
H	6.517579000	0.825395000	-0.273100000
H	5.964177000	-0.478765000	0.811687000
H	6.000100000	-0.743318000	-0.947509000
N	1.146347000	-0.530494000	-0.166472000
H	1.423475000	-1.503231000	-0.037787000
N	-1.629808000	1.466508000	0.042761000
O	3.655951000	-1.517039000	0.019773000
O	-0.426053000	0.982096000	-0.605130000
O	4.521638000	0.560893000	-0.273835000

C	-3.206943000	-2.363220000	-0.762974000
H	-2.412367000	-0.583131000	-1.655779000
C	-1.904163000	-3.332158000	1.016468000
H	-0.111667000	-2.284417000	1.551199000
C	-3.025725000	-3.357381000	0.196607000
H	-4.080295000	-2.380124000	-1.404569000
H	-1.764381000	-4.094633000	1.773710000
H	-3.762134000	-4.145014000	0.306776000
C	-2.207389000	2.397171000	-0.925942000
H	-1.493217000	3.184884000	-1.199242000
H	-2.415044000	1.817512000	-1.827885000
C	-3.496992000	3.002560000	-0.393469000
H	-3.985553000	3.562756000	-1.192758000
H	-4.182148000	2.222859000	-0.052213000
H	-3.315371000	3.693485000	0.432775000
C	-1.215383000	2.055959000	1.321337000
H	-2.144645000	2.292552000	1.845238000
H	-0.742972000	1.250096000	1.890386000
C	-0.300329000	3.273826000	1.260968000
H	-0.792345000	4.133559000	0.800164000
H	-0.015739000	3.558127000	2.276353000
H	0.612480000	3.054337000	0.703403000
C	3.476592000	-0.304390000	-0.164219000
C	5.822989000	-0.005456000	-0.162255000
H	6.517579000	0.825395000	-0.273100000
H	5.964177000	-0.478765000	0.811687000
H	6.000100000	-0.743318000	-0.947509000
N	1.146347000	-0.530494000	-0.166472000
H	1.423475000	-1.503231000	-0.037787000
N	-1.629808000	1.466508000	0.042761000
O	3.655951000	-1.517039000	0.019773000
O	-0.426053000	0.982096000	-0.605130000
O	4.521638000	0.560893000	-0.273835000

azirinopyrrolidine ***anti*-10**

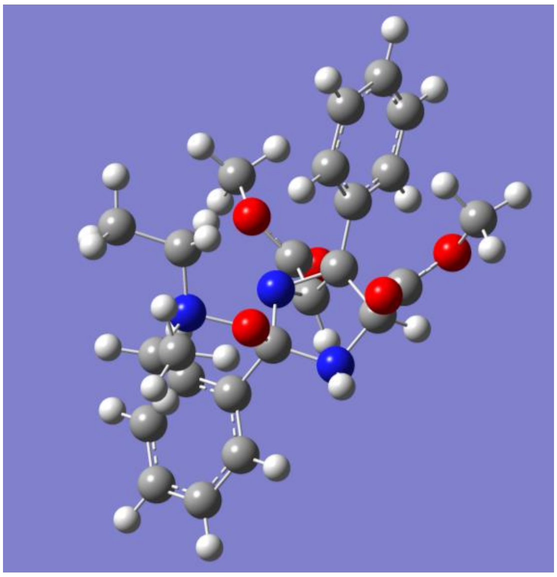

Zero-point correction = 0.506513 (Hartree/Particle)
Thermal correction to Energy = 0.546387
Thermal correction to Enthalpy = 0.547473
Thermal correction to Gibbs Free Energy = 0.427259
Sum of electronic and zero-point Energies = -1471.758865

azirinopyrrolidine ***syn*-10**

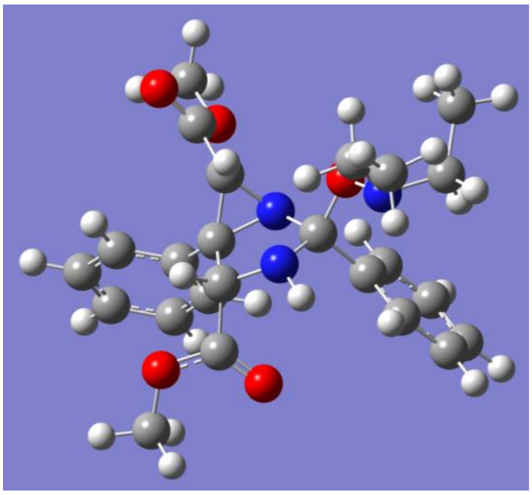

Zero-point correction = 0.506089 (Hartree/Particle)
Thermal correction to Energy = 0.546031
Thermal correction to Enthalpy = 0.547117
Thermal correction to Gibbs Free Energy = 0.425761
Sum of electronic and zero-point Energies = -1471.755864
Sum of electronic and thermal Energies = -1471.715922
Sum of electronic and thermal Enthalpies = -1471.714836

Sum of electronic and thermal Energies = -1471.718991
Sum of electronic and thermal Enthalpies = -1471.717905
Sum of electronic and thermal Free Energies = -1471.838119
Imaginary frequencies = 0

C	0.833320000	-0.606941000	-1.873546000
C	1.091988000	0.376573000	-0.716891000
C	0.327904000	1.656905000	-0.878047000
C	-1.178433000	-0.256190000	-0.642044000
H	-0.152252000	1.817597000	-1.837968000
H	1.222601000	-0.187114000	-2.806135000
C	-2.503056000	0.472101000	-0.817433000
C	-3.407803000	0.032371000	-1.780668000
C	-2.848524000	1.543900000	0.001065000
C	-4.643267000	0.652685000	-1.923288000
H	-3.152342000	-0.797891000	-2.429207000
C	-4.080274000	2.172217000	-0.146466000
H	-2.153861000	1.881822000	0.760114000
C	-4.982426000	1.726518000	-1.106541000
H	-5.339723000	0.299588000	-2.675484000
H	-4.337441000	3.007917000	0.494894000
H	-5.944146000	2.214327000	-1.219610000
C	2.367682000	0.288594000	0.053660000
C	3.450508000	1.096267000	-0.280185000
C	2.497076000	-0.652618000	1.072303000
C	4.655051000	0.967277000	0.402240000
H	3.353879000	1.825771000	-1.077766000
C	3.701347000	-0.784699000	1.751932000
H	1.653992000	-1.285835000	1.325751000
C	4.782865000	0.025298000	1.417618000
H	5.494008000	1.601298000	0.138844000
H	3.795923000	-1.520788000	2.542197000
H	5.723257000	-0.077963000	1.947049000
C	-3.215962000	-1.983633000	1.389624000
H	-3.801884000	-1.673666000	0.521235000
H	-3.790316000	-1.691508000	2.272790000
C	-1.052224000	-1.429110000	2.454846000
H	-0.241105000	-0.711978000	2.322090000
H	-0.625451000	-2.438157000	2.357799000
C	-1.671484000	-1.245155000	3.832343000
H	-0.888006000	-1.299390000	4.591165000
H	-2.158345000	-0.269721000	3.913388000
H	-2.408302000	-2.018835000	4.060950000
C	-2.999022000	-3.494802000	1.368767000
H	-2.507955000	-3.848815000	2.279062000
H	-3.963837000	-4.002133000	1.292281000
H	-2.389677000	-3.788879000	0.511460000
C	1.545330000	-1.943878000	-1.675377000
C	3.661069000	-2.969249000	-1.736540000
H	3.290107000	-3.842616000	-2.272929000
H	4.651077000	-2.695244000	-2.091285000
H	3.684811000	-3.172128000	-0.664522000
N	-0.613664000	-0.691897000	-1.908208000
H	-0.948924000	-1.608354000	-2.176053000
N	-0.163597000	0.586955000	-0.017803000
O	-1.330999000	-1.423585000	0.157824000
O	2.829577000	-1.830361000	-1.994580000

Sum of electronic and thermal Free Energies = -1471.836192
Imaginary frequencies = 0

C	0.575152000	-0.470327000	-1.686415000
C	1.138861000	0.417212000	-0.562023000
C	0.491103000	1.763998000	-0.517695000
C	-1.137882000	0.006800000	-0.096690000
H	-0.149531000	2.027325000	-1.351333000
H	0.858002000	-0.066231000	-2.662715000
C	-1.494920000	-1.136565000	0.852667000
C	-1.082872000	-1.090489000	2.182420000
C	-2.297126000	-2.196247000	0.432425000
C	-1.458148000	-2.089795000	3.074987000
H	-0.462170000	-0.268318000	2.515618000
C	-2.679557000	-3.190238000	1.323526000
H	-2.650136000	-2.246639000	-0.590283000
C	-2.259141000	-3.142403000	2.649418000
H	-1.123615000	-2.041381000	4.105441000
H	-3.308328000	-4.004521000	0.981009000
H	-2.555124000	-3.920057000	3.344641000
C	2.513413000	0.161093000	-0.040128000
C	3.614107000	0.763422000	-0.642632000
C	2.700582000	-0.723384000	1.018846000
C	4.897371000	0.483000000	-0.188376000
H	3.466479000	1.452489000	-1.467967000
C	3.984191000	-1.007375000	1.469832000
H	1.837533000	-1.181537000	1.490267000
C	5.083965000	-0.405625000	0.865918000
H	5.751888000	0.957014000	-0.657696000
H	4.125775000	-1.697637000	2.293750000
H	6.085067000	-0.624635000	1.219864000
C	-4.390085000	0.543922000	0.395976000
H	-4.039418000	-0.113981000	1.193656000
H	-5.270767000	0.063843000	-0.039258000
C	-3.608335000	1.387994000	-1.792662000
H	-2.733674000	1.292474000	-2.437579000
H	-3.679131000	2.441744000	-1.488027000
C	-4.860766000	0.968113000	-2.547216000
H	-4.925877000	1.526950000	-3.483209000
H	-4.830308000	-0.098262000	-2.786419000
H	-5.772226000	1.167735000	-1.978499000
C	-4.754418000	1.913265000	0.963592000
H	-5.209007000	2.562518000	0.210882000
H	-5.476829000	1.790205000	1.774199000
H	-3.872551000	2.415729000	1.366546000
C	1.154951000	-1.880986000	-1.594145000
C	3.074573000	-3.155056000	-2.060463000
H	2.525956000	-3.991916000	-2.493117000
H	4.007866000	-2.994678000	-2.593588000
H	3.268095000	-3.343414000	-1.003356000
N	-0.851978000	-0.382073000	-1.471759000
H	-1.363411000	-1.191525000	-1.789084000
N	0.062409000	0.689367000	0.375748000
O	-2.151371000	1.004613000	-0.046345000
O	2.331530000	-1.937672000	-2.207919000
N	-3.354244000	0.519505000	-0.641388000
C	1.208132000	2.940749000	0.059775000
C	2.315907000	3.852962000	1.913472000

N	-2.011006000	-1.152407000	1.385479000
C	0.797063000	2.929627000	-0.249582000
C	1.379243000	4.042315000	1.730321000
H	1.305270000	3.831631000	2.793850000
H	2.421023000	4.190873000	1.443195000
H	0.795609000	4.927087000	1.475138000
O	1.116346000	3.889104000	-0.913882000
O	0.848080000	2.884539000	1.072180000
O	1.022254000	-2.965160000	-1.308699000

H	2.476289000	3.561882000	2.948148000
H	3.269155000	3.943546000	1.391211000
H	1.776054000	4.798795000	1.863002000
O	1.480718000	3.910430000	-0.610899000
O	1.535081000	2.799154000	1.334496000
O	0.629827000	-2.812695000	-1.039431000

TS1a

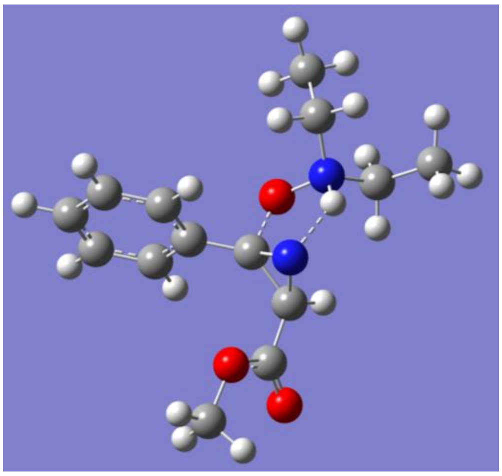

Zero-point correction = 0.328547 (Hartree/Particle)
Thermal correction to Energy = 0.353297
Thermal correction to Enthalpy = 0.354384
Thermal correction to Gibbs Free Energy = 0.267724
Sum of electronic and zero-point Energies = -880.227722
Sum of electronic and thermal Energies = -880.202972
Sum of electronic and thermal Enthalpies = -880.201885
Sum of electronic and thermal Free Energies = -880.288545
Imaginary frequencies = 1

C	2.348443000	3.436725000	0.296413000
C	2.290808000	2.530750000	1.350672000
C	1.582650000	1.342745000	1.208644000
C	0.933628000	1.058714000	0.010191000
C	0.999114000	1.961835000	-1.049060000
C	1.702878000	3.150335000	-0.904109000
C	0.179427000	-0.193455000	-0.140492000
N	-0.116709000	-0.986129000	-1.131028000
C	0.384997000	-1.618946000	0.137415000
C	1.739090000	-2.230199000	0.174353000
O	2.588692000	-1.692640000	-0.699404000
O	2.043599000	-3.120086000	0.940822000
C	3.924727000	-2.205373000	-0.691654000
O	-1.385834000	0.318999000	0.739258000
N	-2.370154000	-0.218917000	-0.040282000
C	-3.252800000	-1.111277000	0.765204000
C	-4.295928000	-1.824272000	-0.075334000
C	-3.069341000	0.839406000	-0.831412000
C	-3.881057000	1.785739000	0.028591000
H	2.898005000	4.364620000	0.407967000
H	2.797538000	2.748789000	2.283896000

TS1b

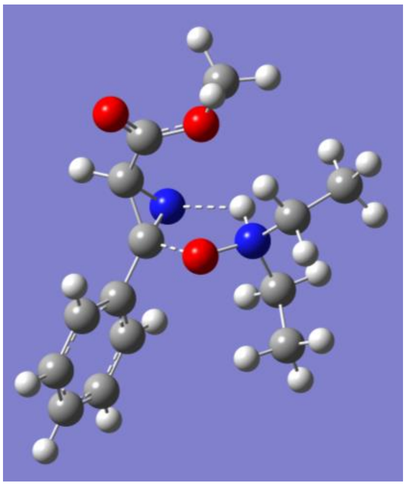

Zero-point correction = 0.330106 (Hartree/Particle)
Thermal correction to Energy = 0.354351
Thermal correction to Enthalpy = 0.355437
Thermal correction to Gibbs Free Energy = 0.271823
Sum of electronic and zero-point Energies = -880.223548
Sum of electronic and thermal Energies = -880.199303
Sum of electronic and thermal Enthalpies = -880.198216
Sum of electronic and thermal Free Energies = -880.281830
Imaginary frequencies = 1

C	-4.597455000	-0.226519000	-0.451774000
C	-3.753777000	-0.948883000	-1.291447000
C	-2.414726000	-1.114022000	-0.959171000
C	-1.915163000	-0.558321000	0.216707000
C	-2.763800000	0.150750000	1.064106000
C	-4.101352000	0.320808000	0.727679000
C	-0.496569000	-0.722412000	0.580025000
N	0.169423000	-0.663876000	1.696159000
C	0.395092000	-1.859342000	0.829414000
C	1.673123000	-2.090153000	0.100261000
O	2.597753000	-1.149578000	0.314251000
O	1.878889000	-3.073403000	-0.576866000
C	3.865404000	-1.346290000	-0.324257000
O	0.211299000	0.428919000	-0.725038000
N	0.995338000	1.287699000	-0.000366000
C	2.183380000	1.696457000	-0.801901000
C	3.256861000	2.359594000	0.040783000
C	0.192824000	2.426614000	0.555293000
C	-0.524576000	3.229153000	-0.509028000
H	-5.641835000	-0.096370000	-0.712138000
H	-4.141111000	-1.384160000	-2.205712000
H	-1.751342000	-1.672894000	-1.610284000

H	1.532626000	0.630369000	2.025234000
H	0.502646000	1.727292000	-1.984491000
H	1.751644000	3.853945000	-1.727457000
H	-0.314695000	-2.135525000	0.790124000
H	4.453787000	-1.655409000	-1.465969000
H	4.391637000	-2.036507000	0.279639000
H	3.925626000	-3.272626000	-0.916704000
H	-2.577971000	-1.819309000	1.244096000
H	-3.702555000	-0.496105000	1.544105000
H	-4.800531000	-2.562598000	0.549540000
H	-5.054081000	-1.139131000	-0.458950000
H	-3.839181000	-2.351636000	-0.916575000
H	-2.269507000	1.364233000	-1.354035000
H	-3.685569000	0.330582000	-1.574908000
H	-4.258464000	2.593652000	-0.600304000
H	-4.740455000	1.291142000	0.485855000
H	-3.260790000	2.220661000	0.813373000
H	-1.833149000	-0.807669000	-0.747213000

H	-2.374413000	0.559889000	1.990214000
H	-4.757647000	0.876784000	1.387593000
H	-0.047073000	-2.806069000	1.141115000
H	4.467306000	-0.480862000	-0.056320000
H	4.334353000	-2.259774000	0.042021000
H	3.743465000	-1.406901000	-1.406570000
H	2.542953000	0.777155000	-1.255475000
H	1.825205000	2.346645000	-1.598985000
H	4.144334000	2.505066000	-0.577170000
H	2.950041000	3.337319000	0.415723000
H	3.538237000	1.731139000	0.890209000
H	-0.516924000	1.963885000	1.239675000
H	0.874163000	3.039898000	1.147075000
H	-1.143382000	2.577906000	-1.126831000
H	-1.172653000	3.954840000	-0.014500000
H	0.164811000	3.783373000	-1.148734000
H	1.309051000	0.734255000	0.829626000

TS2a

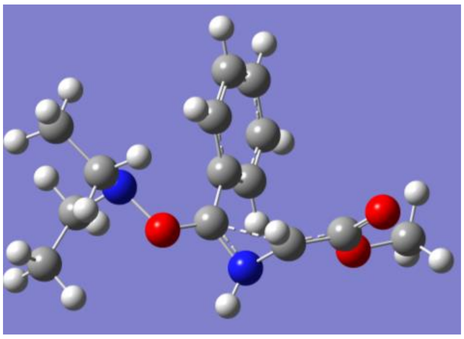

Zero-point correction = 0.327842 (Hartree/Particle)
Thermal correction to Energy = 0.352446
Thermal correction to Enthalpy = 0.353532
Thermal correction to Gibbs Free Energy = 0.268483
Sum of electronic and zero-point Energies = -880.219514
Sum of electronic and thermal Energies = -880.194910
Sum of electronic and thermal Enthalpies = -880.193824
Sum of electronic and thermal Free Energies = -880.278873
Imaginary frequencies = 1

C	1.092989000	1.178366000	-1.164384000
C	0.216324000	0.705942000	-0.184874000
C	-0.229393000	1.569511000	0.821416000
C	0.202491000	2.885241000	0.842837000
C	1.071021000	3.357506000	-0.140243000
C	1.513490000	2.502647000	-1.141797000
H	1.447526000	0.500270000	-1.929373000
H	-0.893080000	1.208747000	1.595113000
H	-0.136103000	3.547443000	1.631174000
H	1.402620000	4.389464000	-0.119889000
H	2.191288000	2.862452000	-1.907166000
C	-0.178643000	-0.712820000	-0.254801000
C	1.425664000	-1.702484000	0.613233000
H	0.907379000	-2.021280000	1.507954000
C	2.789048000	-1.339972000	0.695443000
C	4.683350000	-0.531514000	-0.468061000
H	4.793480000	0.353715000	0.162221000
H	4.934111000	-0.284232000	-1.498421000

TS2b

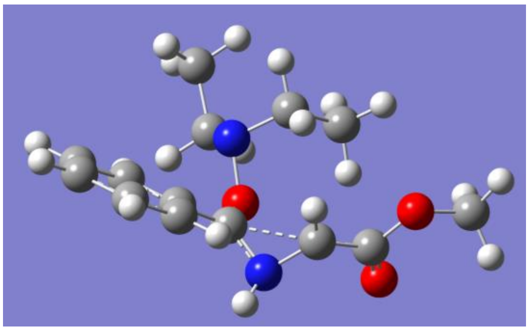

Zero-point correction = 0.327914 (Hartree/Particle)
Thermal correction to Energy = 0.352455
Thermal correction to Enthalpy = 0.353541
Thermal correction to Gibbs Free Energy = 0.269252
Sum of electronic and zero-point Energies = -880.216309
Sum of electronic and thermal Energies = -880.191768
Sum of electronic and thermal Enthalpies = -880.190682
Sum of electronic and thermal Free Energies = -880.274972
Imaginary frequencies = 1

C	-1.221948000	-1.671975000	0.088930000
C	0.486143000	-0.560767000	0.730743000
H	-0.851425000	-1.911296000	-0.899414000
C	1.844211000	-0.765184000	0.233161000
C	2.846230000	0.204507000	0.380538000
C	2.161008000	-2.001098000	-0.344794000
C	4.136709000	-0.066541000	-0.039088000
H	2.621631000	1.154864000	0.842871000
C	3.452909000	-2.257942000	-0.782417000
H	1.393001000	-2.754717000	-0.475864000
C	4.440537000	-1.292764000	-0.628810000
H	4.910938000	0.679937000	0.091480000
H	3.685419000	-3.210837000	-1.241987000
H	5.451128000	-1.493978000	-0.965120000
C	0.401723000	2.882368000	0.684170000
H	-0.526872000	3.101503000	1.225912000
H	1.183954000	2.731134000	1.431396000
C	0.779429000	4.037496000	-0.229896000

H	5.349496000	-1.315588000	-0.101974000
O	3.331571000	-0.971853000	-0.496889000
O	3.460078000	-1.326335000	1.730761000
N	0.696492000	-1.661302000	-0.624110000
H	0.313359000	-2.515971000	-1.012892000
O	-1.419232000	-1.145192000	-0.105321000
N	-2.448265000	-0.136515000	0.044519000
C	-3.930508000	-1.379400000	-1.616756000
H	-4.740295000	-1.604875000	-0.919104000
H	-3.258550000	-2.239159000	-1.659322000
H	-4.372588000	-1.244168000	-2.606098000
C	-4.326007000	0.429936000	1.517417000
H	-5.108900000	0.408455000	0.756510000
H	-3.930774000	1.446378000	1.584032000
H	-4.785198000	0.175757000	2.474230000
C	-3.180365000	-0.110409000	-1.226369000
C	-3.213737000	-0.563137000	1.218051000
H	-2.451277000	0.160437000	-1.993755000
H	-3.866705000	0.735664000	-1.143880000
H	-3.618155000	-1.575069000	1.090562000
H	-2.508902000	-0.593148000	2.052143000

H	0.992903000	4.919354000	0.376729000
H	1.671975000	3.795995000	-0.812142000
H	-0.026914000	4.295830000	-0.919584000
C	-0.796138000	1.598597000	-1.059192000
H	-0.461958000	2.248154000	-1.871783000
H	-0.815169000	0.583538000	-1.459606000
C	-2.184773000	1.999045000	-0.573120000
H	-2.242419000	3.059850000	-0.317628000
H	-2.907843000	1.810611000	-1.369986000
H	-2.482609000	1.410364000	0.297039000
C	-2.574208000	-1.328255000	0.298870000
C	-4.663000000	-1.034482000	-0.780902000
H	-5.065359000	-1.151912000	-1.785969000
H	-5.207592000	-1.680057000	-0.088344000
H	-4.770821000	0.004107000	-0.458872000
N	-0.282542000	-1.582958000	1.164283000
H	0.191838000	-2.415270000	1.491326000
N	0.293939000	1.635001000	-0.074563000
O	-3.112335000	-0.986853000	1.351326000
O	-0.029187000	0.639691000	0.939962000
O	-3.293051000	-1.402776000	-0.864744000

TS3a

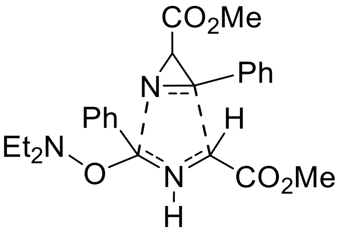

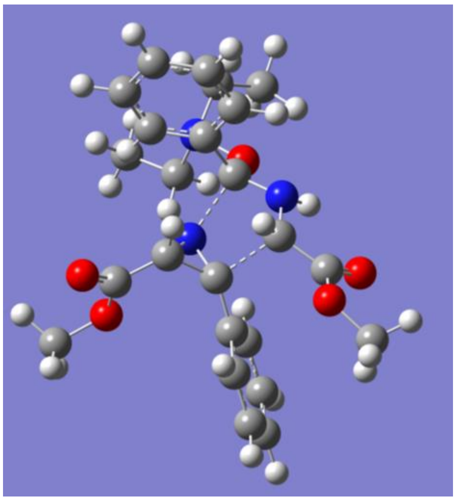

Zero-point correction = 0.496989 (Hartree/Particle)
Thermal correction to Energy = 0.536593
Thermal correction to Enthalpy = 0.537680
Thermal correction to Gibbs Free Energy = 0.422861
Sum of electronic and zero-point Energies = -1471.680757
Sum of electronic and thermal Energies = -1471.641153
Sum of electronic and thermal Enthalpies = -1471.640067
Sum of electronic and thermal Free Energies = -1471.754885
Imaginary frequencies = 1

C	0.804745000	-1.737929000	-0.707951000
C	1.177313000	0.233358000	-0.285842000

TS3b

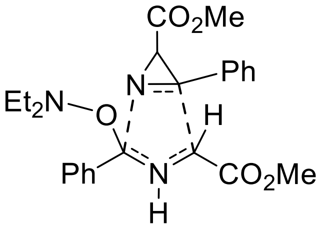

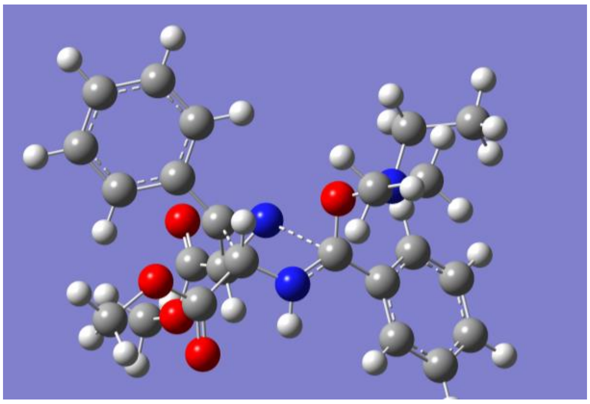

Zero-point correction = 0.500088 (Hartree/Particle)
Thermal correction to Energy = 0.540923
Thermal correction to Enthalpy = 0.542009
Thermal correction to Gibbs Free Energy = 0.420230
Sum of electronic and zero-point Energies = -1471.679379
Sum of electronic and thermal Energies = -1471.638544
Sum of electronic and thermal Enthalpies = -1471.637458
Sum of electronic and thermal Free Energies = -1471.759237
Imaginary frequencies = 1

C	-2.894009000	1.933867000	1.428598000
C	-2.490562000	1.220280000	0.290522000
C	-3.035397000	1.557141000	-0.957570000
C	-3.953815000	2.594549000	-1.064019000
C	-4.346959000	3.306333000	0.071474000

C	0.451014000	1.029452000	-1.319175000
C	-1.283507000	-0.868830000	0.119096000
H	0.025498000	0.502849000	-2.167898000
H	0.638604000	-1.688800000	-1.778884000
C	-2.169280000	-0.704998000	-1.075739000
C	-2.434958000	-1.826977000	-1.882008000
C	-2.772541000	0.525047000	-1.391246000
C	-3.288621000	-1.720539000	-2.985293000
H	-1.978027000	-2.778057000	-1.631546000
C	-3.626057000	0.626246000	-2.491858000
H	-2.558914000	1.389374000	-0.778880000
C	-3.887585000	-0.494334000	-3.291442000
H	-3.484916000	-2.593418000	-3.599687000
H	-4.080073000	1.582089000	-2.731707000
H	-4.548430000	-0.409218000	-4.148318000
C	2.452803000	0.416598000	0.421301000
C	3.664644000	0.486740000	-0.289710000
C	2.476680000	0.484618000	1.825465000
C	4.876522000	0.624264000	0.393051000
H	3.650558000	0.431259000	-1.373464000
C	3.690155000	0.615765000	2.505673000
H	1.539242000	0.446645000	2.369993000
C	4.893541000	0.686378000	1.792890000
H	5.805611000	0.688004000	-0.165335000
H	3.697211000	0.666530000	3.590033000
H	5.835409000	0.792826000	2.322077000
C	-4.122151000	-0.621004000	2.053463000
H	-4.368596000	-1.301006000	1.233046000
H	-4.937759000	0.108462000	2.118749000
C	-2.367474000	1.144398000	2.548858000
H	-1.450419000	1.499737000	2.073813000
H	-2.097278000	0.677104000	3.507402000
C	-3.343142000	2.305467000	2.765337000
H	-2.851919000	3.089476000	3.351636000
H	-3.649988000	2.736345000	1.806675000
H	-4.243393000	1.999660000	3.310246000
C	-3.977086000	-1.398289000	3.369167000
H	-3.819969000	-0.734022000	4.226056000
H	-4.889135000	-1.975378000	3.560105000
H	-3.136035000	-2.095534000	3.315534000
C	1.934885000	-2.560126000	-0.260588000
C	4.167309000	-3.308431000	-0.811966000
H	3.947926000	-4.305773000	-0.426469000
H	4.757759000	-3.357805000	-1.724683000
H	4.677301000	-2.721207000	-0.045599000
N	-0.334347000	-1.882586000	0.110981000
H	-0.120234000	-2.265706000	1.031680000
N	-0.092252000	0.701442000	0.085133000
O	-1.849348000	-0.861978000	1.409506000
O	2.922557000	-2.632330000	-1.208508000
N	-2.939038000	0.152606000	1.599059000
C	0.730174000	2.447967000	-1.645471000
C	1.522026000	4.581448000	-0.834581000
H	1.892942000	4.958180000	0.116441000
H	2.280579000	4.667564000	-1.615911000
H	0.622559000	5.116581000	-1.147748000
O	0.565346000	2.942905000	-2.770514000
O	1.210708000	3.169719000	-0.581116000
O	2.024100000	-3.083908000	0.864113000
C	-3.816173000	2.971490000	1.316231000
H	-2.519649000	1.665765000	2.410109000
H	-2.717241000	1.018284000	-1.840210000
H	-4.360834000	2.852492000	-2.034797000
H	-5.065203000	4.113464000	-0.013978000
H	-4.126551000	3.509561000	2.204193000
C	-1.481174000	0.154443000	0.365157000
C	0.553446000	-0.653726000	1.346225000
H	0.431079000	-1.677892000	1.027291000
C	1.440271000	-0.375068000	2.472982000
C	3.349205000	-1.116143000	3.671573000
H	2.868514000	-1.035950000	4.646819000
H	3.999578000	-1.986582000	3.639697000
H	3.917258000	-0.208690000	3.465312000
C	-3.224543000	-2.096796000	-1.637437000
H	-2.895687000	-3.140060000	-1.736787000
H	-2.624708000	-1.505187000	-2.328152000
C	-4.702395000	-1.949466000	-1.999282000
H	-5.351649000	-2.524656000	-1.338206000
H	-5.007324000	-0.902008000	-1.952240000
H	-4.860299000	-2.308316000	-3.020123000
C	-2.769034000	-2.646649000	0.735967000
H	-2.403064000	-2.163859000	1.644139000
H	-2.012670000	-3.373164000	0.407484000
C	-4.086365000	-3.355855000	1.044888000
H	-4.443484000	-3.951245000	0.203045000
H	-3.928289000	-4.035633000	1.885473000
H	-4.864236000	-2.641692000	1.324700000
N	-2.935225000	-1.586214000	-0.277168000
N	-0.590640000	0.130383000	1.355689000
H	-0.505502000	0.971470000	1.921939000
O	-1.578097000	-0.962232000	-0.386900000
O	1.383380000	0.636485000	3.161062000
O	2.362441000	-1.338626000	2.641823000
C	2.300685000	-0.998462000	-0.879826000
C	1.844431000	-2.031684000	-1.709405000
C	2.715845000	-3.035256000	-2.124480000
C	4.052510000	-3.016244000	-1.720449000
C	4.511593000	-1.987817000	-0.897373000
C	3.639397000	-0.986216000	-0.473693000
H	0.809046000	-2.038719000	-2.030223000
H	2.354435000	-3.829830000	-2.767811000
H	4.731060000	-3.795960000	-2.047399000
H	5.549302000	-1.964880000	-0.583938000
H	3.998651000	-0.189885000	0.168139000
C	1.369344000	0.063587000	-0.456631000
C	1.392115000	1.533239000	-0.324424000
H	1.089464000	1.977068000	0.621915000
C	2.310965000	2.384171000	-1.126259000
C	3.627023000	4.363305000	-1.061101000
H	3.169780000	4.786264000	-1.956029000
H	3.828251000	5.144576000	-0.332280000
H	4.548951000	3.846511000	-1.329471000
N	0.400961000	0.695318000	-1.084806000
O	2.713112000	3.455805000	-0.410374000
O	2.672532000	2.172843000	-2.266188000

TS4a

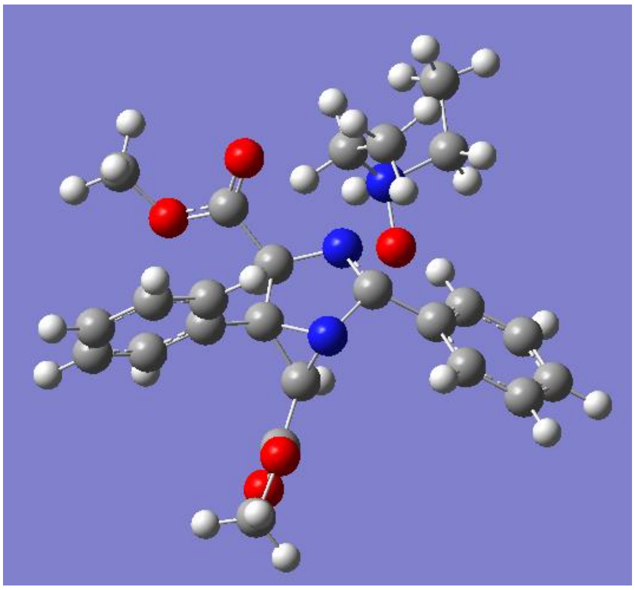

Zero-point correction = 0.503884 (Hartree/Particle)
Thermal correction to Energy = 0.542955
Thermal correction to Enthalpy = 0.544041
Thermal correction to Gibbs Free Energy = 0.425685
Sum of electronic and zero-point Energies = -1471.729582
Sum of electronic and thermal Energies = -1471.690511
Sum of electronic and thermal Enthalpies = -1471.689425
Sum of electronic and thermal Free Energies = -1471.807782
Imaginary frequencies = 1

C	0.386840000	-0.583815000	-1.544726000
C	0.994465000	0.418052000	-0.524441000
C	0.435731000	1.798569000	-0.700754000
C	-1.293461000	0.193692000	-0.177270000
H	-0.141381000	1.973166000	-1.604112000
H	0.558484000	-0.203679000	-2.560001000
C	-2.484285000	1.122289000	-0.276060000
C	-3.330971000	1.069483000	-1.377525000
C	-2.734201000	2.048932000	0.737283000
C	-4.427127000	1.923867000	-1.462096000
H	-3.114218000	0.356790000	-2.164428000
C	-3.821764000	2.907847000	0.649234000
H	-2.069846000	2.097921000	1.592750000
C	-4.674446000	2.844956000	-0.450703000
H	-5.083892000	1.874522000	-2.323769000
H	-4.005289000	3.629375000	1.437740000
H	-5.523554000	3.515969000	-0.519234000
C	2.325818000	0.170145000	0.102064000
C	3.494874000	0.547806000	-0.554614000
C	2.406035000	-0.475335000	1.332596000
C	4.733827000	0.279895000	0.014231000
H	3.432488000	1.049951000	-1.514362000
C	3.645178000	-0.748047000	1.900739000
H	1.491642000	-0.743075000	1.850671000
C	4.811139000	-0.371712000	1.241757000
H	5.639906000	0.579764000	-0.500060000

TS4b

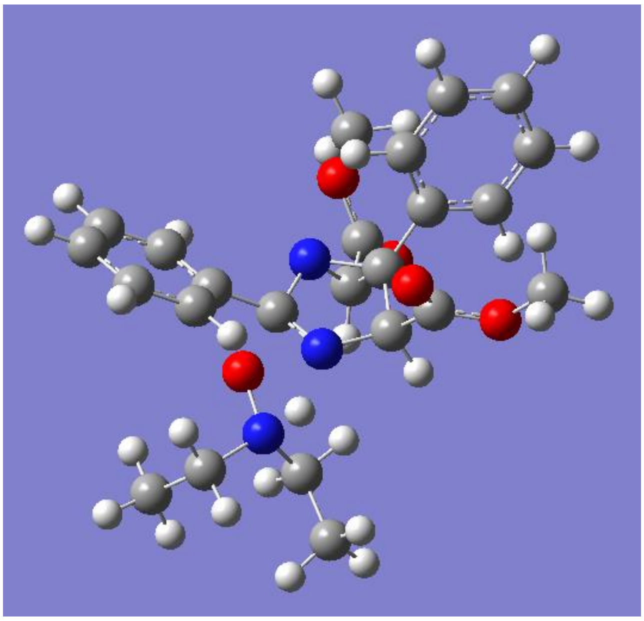

Zero-point correction = 0.502817 (Hartree/Particle)
Thermal correction to Energy = 0.542170
Thermal correction to Enthalpy = 0.543256
Thermal correction to Gibbs Free Energy = 0.424047
Sum of electronic and zero-point Energies = -1471.723603
Sum of electronic and thermal Energies = -1471.684249
Sum of electronic and thermal Enthalpies = -1471.683163
Sum of electronic and thermal Free Energies = -1471.802373
Imaginary frequencies = 1

C	0.393791000	1.448066000	-0.430208000
C	1.087910000	0.237716000	0.254157000
C	0.503790000	-0.138924000	1.570458000
C	-1.120663000	-0.253948000	-0.268675000
H	-0.182570000	0.555820000	2.032490000
H	0.370739000	2.307227000	0.249550000
C	-1.915804000	-1.273349000	-1.029416000
C	-2.102299000	-2.551856000	-0.503243000
C	-2.482939000	-0.948965000	-2.257747000
C	-2.860191000	-3.488569000	-1.192084000
H	-1.649258000	-2.808021000	0.447551000
C	-3.245687000	-1.887219000	-2.947368000
H	-2.315621000	0.039976000	-2.667741000
C	-3.437898000	-3.157271000	-2.415930000
H	-3.000977000	-4.479667000	-0.775249000
H	-3.684759000	-1.626404000	-3.903949000
H	-4.029257000	-3.889205000	-2.954814000
C	2.486580000	-0.135645000	-0.118089000
C	3.561952000	0.479636000	0.518875000
C	2.721905000	-1.066057000	-1.126508000
C	4.864904000	0.166636000	0.149842000
H	3.377203000	1.207237000	1.302365000
C	4.024601000	-1.379716000	-1.495506000
H	1.879858000	-1.546535000	-1.612066000
C	5.097430000	-0.763241000	-0.858774000

H	3.700575000	-1.248228000	2.861051000
H	5.777791000	-0.579149000	1.686734000
C	-1.006611000	-2.913895000	1.512665000
H	0.001017000	-2.504820000	1.498824000
H	-0.970530000	-3.874951000	0.999980000
C	-3.245262000	-2.351765000	0.500894000
H	-3.645699000	-1.594657000	-0.174234000
H	-3.735265000	-2.234129000	1.468298000
C	-3.436549000	-3.743285000	-0.075075000
H	-4.493671000	-3.886084000	-0.304609000
H	-2.869048000	-3.868948000	-1.000499000
H	-3.140024000	-4.524065000	0.627825000
C	-1.537905000	-3.044828000	2.927867000
H	-2.511020000	-3.539979000	2.957120000
H	-0.841637000	-3.650068000	3.511339000
H	-1.625843000	-2.067452000	3.405434000
C	1.098876000	-1.929182000	-1.496442000
C	3.087765000	-3.036054000	-2.091558000
H	2.592917000	-3.910092000	-2.515941000
H	3.978046000	-2.788304000	-2.663910000
H	3.347627000	-3.227654000	-1.049363000
N	-1.022468000	-0.650384000	-1.229583000
H	-1.359574000	-1.840991000	-0.376064000
N	-0.099457000	0.883170000	0.304355000
O	-1.688347000	-0.705365000	1.110060000
O	2.238803000	-1.885801000	-2.180331000
N	-1.810351000	-2.005253000	0.662631000
C	1.195890000	3.006724000	-0.264992000
C	2.294162000	4.108958000	1.489282000
H	2.427559000	3.940330000	2.554592000
H	3.257402000	4.093479000	0.978053000
H	1.798073000	5.063939000	1.313817000
O	1.544640000	3.859815000	-1.050089000
O	1.474119000	3.026965000	1.029543000
O	0.698883000	-2.913289000	-0.922885000

H	5.698308000	0.646901000	0.649868000
H	4.202728000	-2.107841000	-2.278797000
H	6.113841000	-1.008715000	-1.145591000
C	-4.100427000	1.264125000	0.284951000
H	-4.058127000	0.589240000	-0.569822000
H	-4.319441000	2.264406000	-0.093088000
C	-2.503081000	2.166269000	2.002603000
H	-1.439246000	2.105495000	2.230484000
H	-3.048689000	1.744937000	2.848904000
C	-2.894652000	3.609791000	1.737860000
H	-2.578583000	4.219373000	2.585976000
H	-2.401026000	3.995533000	0.842295000
H	-3.973255000	3.732762000	1.623973000
C	-5.137831000	0.802419000	1.291533000
H	-5.264163000	1.518290000	2.107012000
H	-6.101245000	0.697958000	0.789254000
H	-4.862623000	-0.166481000	1.711650000
C	1.202369000	1.872751000	-1.652021000
C	3.102021000	3.100691000	-2.299262000
H	2.619595000	3.545724000	-3.169791000
H	3.770545000	3.816553000	-1.827941000
H	3.652174000	2.206349000	-2.597173000
N	-0.925206000	0.975431000	-0.797629000
H	-1.958697000	1.553003000	-0.049279000
N	0.076944000	-0.813489000	0.344655000
O	-2.256053000	0.023528000	1.064343000
O	2.130262000	2.763043000	-1.302318000
N	-2.717654000	1.293645000	0.825565000
C	1.303759000	-0.898634000	2.579618000
C	2.507147000	-2.849876000	3.073140000
H	2.678057000	-3.804602000	2.582929000
H	3.450515000	-2.322184000	3.218437000
H	2.014030000	-2.997613000	4.034190000
O	1.607408000	-0.410086000	3.644477000
O	1.659685000	-2.109925000	2.184370000
O	1.066353000	1.444986000	-2.769495000

TS1

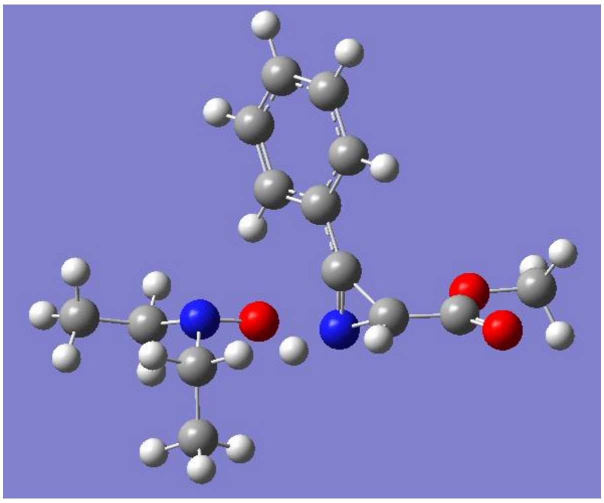

Zero-point correction = 0.322641 (Hartree/Particle)
Thermal correction to Energy = 0.348182
Thermal correction to Enthalpy = 0.349268
Thermal correction to Gibbs Free Energy = 0.259824
Sum of electronic and zero-point Energies = -880.178147
Sum of electronic and thermal Energies = -880.152606

Sum of electronic and thermal Enthalpies = -880.151520
Sum of electronic and thermal Free Energies = -880.240964
Imaginary frequencies = 1

C	0.854573000	4.049693000	0.402547000
C	1.346775000	3.198968000	1.388500000
C	1.309060000	1.825582000	1.199586000
C	0.776056000	1.303841000	0.015792000
C	0.281646000	2.161303000	-0.976335000
C	0.324044000	3.530814000	-0.778471000
C	0.729875000	-0.109832000	-0.172244000
N	0.550957000	-1.036466000	-1.088453000
C	1.189135000	-1.431568000	0.220126000
C	2.637739000	-1.806124000	0.211972000
O	3.324613000	-1.176792000	-0.731527000
O	3.113636000	-2.582532000	1.005347000
C	4.730053000	-1.457081000	-0.801177000
O	-1.397385000	-0.692774000	0.057607000
N	-2.677600000	-0.378631000	0.375754000
C	-3.368799000	0.138249000	-0.805025000
C	-4.779005000	0.625628000	-0.509937000
C	-3.333846000	-1.486544000	1.073251000
C	-3.587252000	-2.733225000	0.226257000
H	0.885936000	5.122738000	0.552585000
H	1.759971000	3.608082000	2.302621000
H	1.687227000	1.150581000	1.959042000
H	-0.130146000	1.744013000	-1.887871000
H	-0.055076000	4.199891000	-1.541745000
H	0.581076000	-1.997001000	0.917248000
H	5.104348000	-0.859678000	-1.628005000
H	5.217547000	-1.168328000	0.130317000
H	4.894413000	-2.517872000	-0.991320000
H	-2.757566000	0.965624000	-1.171512000
H	-3.387541000	-0.624707000	-1.600579000
H	-5.190347000	1.113706000	-1.396435000
H	-5.450753000	-0.194364000	-0.244014000
H	-4.777929000	1.349457000	0.309383000
H	-2.687761000	-1.733929000	1.918775000
H	-4.273129000	-1.111541000	1.489189000
H	-3.971125000	-3.539137000	0.856766000
H	-4.322727000	-2.546977000	-0.561087000
H	-2.661351000	-3.077936000	-0.241156000
H	-0.565815000	-1.218385000	-1.003773000

Table S2. Energies (au) and cartesian coordinates of stationary points for compounds **1a** and Et_2N-O· and transition state TS1^{rad} (calculations in ub3lyp/6-31g(d)).

nitroxyl radical Et_2N-O·

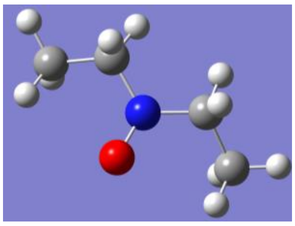

Zero-point correction = 0.141765 (Hartree/Particle)
Thermal correction to Energy = 0.151635
Thermal correction to Enthalpy = 0.152721
Thermal correction to Gibbs Free Energy = 0.101605
Sum of electronic and zero-point Energies = -288.216750
Sum of electronic and thermal Energies = -288.206879
Sum of electronic and thermal Enthalpies = -288.205793
Sum of electronic and thermal Free Energies = -288.256909
Imaginary frequencies = 0

C	0.273864000	0.662649000	1.269491000
N	-0.106237000	0.018409000	0.000000000
C	0.273864000	0.662649000	-1.269491000
O	-1.144763000	-0.740507000	0.000000000
C	0.273864000	-0.326314000	2.429648000
C	0.273864000	-0.326314000	-2.429648000
H	1.265613000	1.101956000	1.127064000
H	-0.428483000	1.487113000	1.466252000
H	-0.428483000	1.487113000	-1.466252000
H	1.265613000	1.101956000	-1.127064000
H	0.536470000	0.194398000	3.355895000
H	-0.713158000	-0.778644000	2.549982000
H	1.004066000	-1.125230000	2.262643000
H	0.536470000	0.194398000	-3.355895000
H	1.004066000	-1.125230000	-2.262643000
H	-0.713158000	-0.778644000	-2.549982000

TS1^{rad}

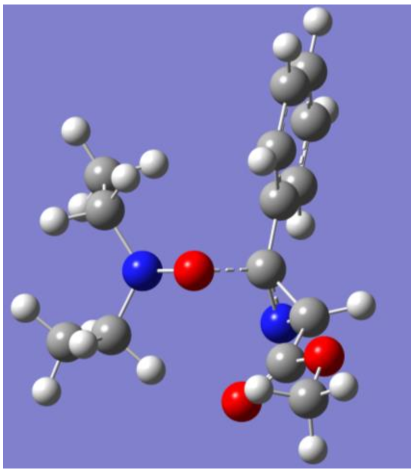

Zero-point correction = 0.312280 (Hartree/Particle)
Thermal correction to Energy = 0.337673
Thermal correction to Enthalpy = 0.338759
Thermal correction to Gibbs Free Energy = 0.249729
Sum of electronic and zero-point Energies = -879.639735
Sum of electronic and thermal Energies = -879.614341
Sum of electronic and thermal Enthalpies = -879.613255
Sum of electronic and thermal Free Energies = -879.702285
Imaginary frequencies = 1

C	-3.728395000	-2.228520000	-0.187310000
C	-3.408642000	-1.715282000	1.071373000
C	-2.147307000	-1.161531000	1.302125000
C	-1.201681000	-1.109543000	0.270034000
C	-1.525173000	-1.628432000	-0.991891000
C	-2.782738000	-2.186389000	-1.217332000
C	0.141970000	-0.527530000	0.536013000
N	0.691735000	-0.262888000	1.739420000
C	1.412345000	-1.217075000	0.857816000
C	2.733035000	-0.797509000	0.282637000
O	3.243106000	0.302608000	0.361356000
O	3.320207000	-1.851054000	-0.326048000
C	4.616890000	-1.601169000	-0.900214000
N	-0.151340000	1.811134000	-0.391650000
C	0.808955000	2.825807000	0.048989000
C	-1.280623000	2.143309000	-1.273299000
C	0.150826000	4.039641000	0.699957000
C	-2.613554000	2.315369000	-0.539482000
H	-4.707812000	-2.663658000	-0.365731000
H	-4.137879000	-1.749057000	1.876125000
H	-1.884166000	-0.773928000	2.281597000
H	-0.791659000	-1.589022000	-1.791813000
H	-3.026069000	-2.589006000	-2.196709000
H	1.367176000	-2.276245000	1.117851000
H	4.932809000	-2.549999000	-1.333236000
H	4.551428000	-0.831617000	-1.673462000
H	5.321319000	-1.279931000	-0.128796000
H	1.470565000	2.311406000	0.750870000

H	1.426446000	3.138732000	-0.808769000
H	-1.348296000	1.334712000	-2.004316000
H	-1.024631000	3.061261000	-1.818805000
H	0.932711000	4.729547000	1.033614000
H	-0.494373000	4.585509000	0.004140000
H	-0.443767000	3.748135000	1.571633000
H	-3.401112000	2.544924000	-1.266073000
H	-2.571360000	3.133551000	0.185386000
H	-2.887011000	1.398124000	-0.010324000
O	0.421371000	0.619941000	-0.612067000

azirine **1a**

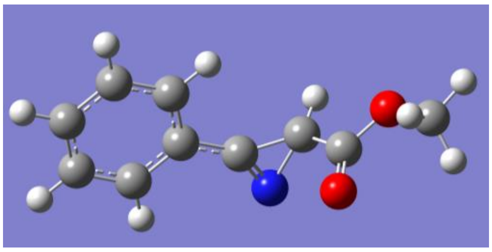

Zero-point correction = 0.170668 (Hartree/Particle)
Thermal correction to Energy = 0.185582
Thermal correction to Enthalpy = 0.186668
Thermal correction to Gibbs Free Energy = 0.123117
Sum of electronic and zero-point Energies = -591.469160
Sum of electronic and thermal Energies = -591.454246
Sum of electronic and thermal Enthalpies = -591.453160
Sum of electronic and thermal Free Energies = -591.516711
Imaginary frequencies = 0

C	3.872165000	0.891656000	-0.220373000
C	3.702029000	-0.475254000	-0.470888000
C	2.461196000	-1.070937000	-0.268590000
C	1.382240000	-0.292542000	0.188249000
C	1.555249000	1.077617000	0.439368000
C	2.801156000	1.666522000	0.233790000
C	0.089095000	-0.901502000	0.401807000
N	-0.546310000	-1.989042000	0.335579000
C	-1.298893000	-0.725135000	0.820753000
C	-2.291077000	-0.151825000	-0.134052000
O	-3.294039000	0.453459000	0.528515000
O	-2.211279000	-0.215200000	-1.347002000
C	-4.310554000	1.065103000	-0.289985000
H	4.842785000	1.352382000	-0.380035000
H	4.537808000	-1.072043000	-0.823505000
H	2.315877000	-2.129966000	-0.459686000
H	0.714453000	1.667306000	0.792324000
H	2.937736000	2.726165000	0.426855000
H	-1.561891000	-0.684082000	1.875068000
H	-3.874264000	1.842562000	-0.921834000
H	-4.795291000	0.314867000	-0.919216000
H	-5.026128000	1.497811000	0.408391000
